# Cold-pressed extraction of perilla seed oil enriched with alpha-linolenic acid mitigates tumour progression and restores gut microbial homeostasis in the AOM/DSS mice model of colitis-associated colorectal cancer

**DOI:** 10.1371/journal.pone.0315172

**Published:** 2024-12-09

**Authors:** Chawin Korsirikoon, Peerapa Techaniyom, Aikkarach Kettawan, Thanaporn Rungruang, Chanatip Metheetrairut, Pinidphon Prombutara, Aurawan Kringkasemsee Kettawan

**Affiliations:** 1 Doctor of Philosophy Program in Nutrition, Faculty of Medicine Ramathibodi Hospital and Institute of Nutrition, Mahidol University, Bangkok, Thailand; 2 Institute of Nutrition, Mahidol University, Nakhon Pathom, Thailand; 3 Department of Anatomy, Faculty of Medicine Siriraj Hospital, Mahidol University, Bangkok, Thailand; 4 Department of Biochemistry, Faculty of Medicine Siriraj Hospital, Mahidol University, Bangkok, Thailand; 5 OMICS Sciences and Bioinformatics Center, Faculty of Science, Chulalongkorn University, Bangkok, Thailand; Museu Paraense Emilio Goeldi, BRAZIL

## Abstract

The present investigation explores into the influence of dietary nutrients, particularly alpha-linolenic acid (ALA), a plant-derived omega-3 fatty acid abundant in perilla seed oil (PSO), on the development of colitis-associated colorectal cancer (CRC). The study employs a mouse model to scrutinize the effects of ALA-rich PSO in the context of inflammation-driven CRC. Perilla seeds were subjected to oil extraction, and the nutritional composition of the obtained oil was analysed. Male ICR mice, initiated at four weeks of age, were subjected to diets comprising 5%, 10%, or 20% PSO, 10% fish oil, or 5% soybean oil. All groups, with the exception of the control group (5% soybean oil), underwent induction with azoxymethane (AOM) and dextran sulphate sodium (DSS) to instigate CRC. Disease development, colon samples, preneoplastic lesions, dysplasia, and biomarkers were meticulously evaluated. Furthermore, gut microbiota composition was elucidated through 16S rRNA sequencing. The analysis revealed that PSO contained 61.32% ALA and 783.90 mg/kg tocopherols. Mice subjected to diets comprising 5% soybean or 10% fish oil exhibited higher tumour incidence, burden, multiplicity, and aberrant crypt counts. Remarkably, these parameters were significantly reduced in mice fed a 5% PSO diet. Additionally, 5% PSO-fed mice displayed reduced proliferative and pro-inflammatory markers in colon tissues, coupled with an alleviation of AOM/DSS-induced gut dysbiosis. Notably, PSO demonstrated inhibitory effects on colitis-associated CRC in the AOM/DSS mice model, achieved through the suppression of proliferative and pro-inflammatory protein levels, and mitigation of gut dysbiosis, with discernible efficacy observed at a 5% dietary concentration.

## Introduction

Colorectal cancer (CRC) emerges as a formidable adversary in the global public health domain, manifesting a substantial challenge characterized by high incidence and mortality rates. Globally, CRC accounted for approximately 10% of newly diagnosed cancer cases in 2020, with over 1.9 million cases. It was the second leading cause of cancer-related mortality, contributing to 9.4% of cancer deaths [1]. In Thailand, CRC represented 11.1% of newly diagnosed cancers and was the third leading cause of cancer-related deaths, accounting for 9.2% of the total cancer deaths [2]. These statistical insights underscore the imminent concern that CRC poses within the broader spectrum of non-communicable diseases.

CRC arises due to multiple factors, with environmental and lifestyle choices contributing to up to 75% of cases. Meanwhile, 15–20% of cases are linked to a family history of CRC, and 5–10% stem from hereditary conditions like familial adenomatous polyposis (FAP) and hereditary nonpolyposis colorectal cancer (HNPCC), or Lynch syndrome. Risk factors for CRC can be classified into non-modifiable (genetic) and modifiable (environmental) categories. Notably, dietary habits and nutritional practices play a pivotal role in either preventing or promoting colorectal carcinogenesis [3–5].

CRC pathogenesis involves intricate molecular pathways, including the Wnt/β-catenin, receptor tyrosine kinase (RTK), and transforming growth factor-β (TGF-β) signalling pathways, all of which play critical roles in regulating cell proliferation and survival. The development of CRC is fundamentally rooted in the progressive accumulation of aberrant genomic and epigenomic changes, including chromosomal instability, microsatellite instability, and CpG island methylator phenotype [6–8]. These deviations perturb the normal regulatory mechanisms of genes associated with crucial cellular processes such as cell growth, proliferation, DNA repair machinery, and apoptosis.

Contemporary findings posit a substantial involvement of the intestinal microbiome, a heterogeneous assembly of microorganisms residing in the gastrointestinal tract, in the pathogenesis of CRC. Although the precise mechanisms through which these intestinal bacteria participate in CRC development remain incompletely elucidated, numerous investigations have signified distinctions in the composition of the intestinal microbiome between individuals afflicted with colonic adenomas/CRC and those exhibiting normal intestinal function [9, 10]. Furthermore, persistent low-grade inflammation within the intestinal milieu, incited by diverse environmental factors, has been postulated to potentially modulate the composition of the intestinal microbiome antecedent to the initiation of CRC.

Among various risk factors, dietary components have been recognised as both risk factors and protective agents against CRC. Among these components, lipids have garnered particular attention due to their strong correlation with CRC risk [11]. High-fat diets, particularly those abundant in saturated fats, are associated with an increased susceptibility to CRC incidence and heightened mortality rates [12, 13]. Conversely, diets rich in polyunsaturated fatty acids (PUFAs) demonstrates an inverse relationship with CRC. Notably, omega-3 (n-3) PUFAs, encompassing α-linolenic acid (ALA), eicosapentaenoic acid (EPA), and docosahexaenoic acid (DHA), exhibit chemopreventive effects against various solid tumours, including CRC [14–16]. Specifically, ALA, an n-3 PUFA derived from various plant sources, holds significant promise in mitigating CRC [17–20].

Perilla seed oil (PSO), sourced from the seeds of *Perilla frutescens*, emerges as a commendable source of n-3 PUFA, particularly in the form of ALA [21, 22]. ALA exerts anticancer effects through anti-inflammatory mechanisms, such as the suppression of NF-κB and COX-2 expression [23], and by inhibiting the Wnt/β-catenin signalling pathway, which regulates cell cycle progression via cyclin D1 [24]. Additionally, ALA contributes to maintaining gut microbiota homeostasis by promoting eubiosis, which enhances intestinal epithelial integrity and reduces local inflammation, both of which are critical in CRC prevention [25]. Besides ALA, PSO contains other lipophilic compounds, such as tocopherols and phytosterols [26], which have antioxidant and anti-inflammatory properties that may further inhibit CRC development by reducing oxidative stress, aberrant cell proliferation, and inflammation in the colon [27, 28]. These bioactive compounds contribute to the overall efficacy of perilla oil in mitigating the progression of CRC, making it a promising candidate for dietary intervention in cancer prevention.

Notably, various investigations consistently affirm that even small amounts of PSO are associated with reduced tumour incidence, fewer intestinal preneoplastic lesions, and decreased levels of specific biological markers linked to CRC in rodent models [29–33]. These findings collectively indicate that ALA derived from PSO holds significant promise as a chemopreventive agent for CRC.

The method of oil extraction plays a crucial role in preserving the bioactive compounds of PSO, which may impact its efficacy in CRC prevention. Conventional methods, such as hot-pressing or solvent extraction, often require refining to remove impurities introduced by chemical solvents or extreme heat. In contrast, cold-pressed extraction relies solely on mechanical force, without the use of external heat or chemical solvents. Consequently, cold-pressed oils retain higher levels of heat-sensitive nutritive compounds and do not require post-extraction refinement [34, 35].

This study aims to examine the fatty acid composition, bioactive components, and antioxidant potential of PSO, while evaluating its tumour-suppressive effects and its impact on the intestinal microbiota in a mouse model of colitis-associated CRC induced by azoxymethane (AOM) and dextran sulphate sodium (DSS).

## Materials and methods

### PSO preparation

Perilla seeds were procured from the indigenous populations and nearby residents of Pa Daet subdistrict, Mae Suai district, Chiang Rai province, Thailand (specimen number: PBM 005745, deposited at Mahidol University’s Herbarium). Subsequently, the seeds were cleaned, dried, and cold-pressed to extract perilla oil. The obtained oil was subjected to filtration using Whatman filter paper (10 µm pore size, 145 µm thickness), followed by storage in 150 mL plastic bottles in a dark at room-temperature, approximately 25±2°C.

### Analysis of fatty acid composition of PSO

The fatty acid composition of the oil samples was determined employing gas chromatography (GC) in accordance with the methodology outlined by Kang and Wang [36]. For sample preparation and saponification, approximately 5 drops of PSO were mixed with 1 mL of methanolic potassium hydroxide (KOH∙CH_3_OH) in a glass test tube. The mixture was subsequently heated in a hot water bath at 95–100°C for 5 min. The saponified fatty acids were then transformed into fatty acid methyl esters (FAMEs) by mixing with 14% (v/v) boron trifluoride-methanol solution (BF_3_∙CH_3_OH) and subjecting the mixture to heating in a hot water bath at 95–100°C for 15 min. Following cooling and phase separation, FAMEs were extracted using petroleum ether. After vaporization in a hot air oven at 60°C overnight, 1 mL of heptadecanoic acid (C17:0) was added as an internal standard before analysis. The FAMEs underwent analyses utilizing the Agilent 6890N Network Gas Chromatograph system equipped with a Restek Rt-2560 capillary column. Fatty acid peaks were identified by comparison with the heptadecanoic acid (C17:0) internal standard, and the data were expressed as the percentage of all fatty acids.

### Determination of tocopherol content of PSO

The quantification of total tocopherol content was executed employing high-performance liquid chromatography (HPLC) in accordance with the methodology outlined by Fromm et al [37]. This analytical approach facilitated the separation of distinct tocopherol forms, namely α-, β-, γ-, and δ-tocopherol, present within the sample. The mobile phase composed of methanol/water (96:4; v/v, eluent A) and methanol/methyl tert-butyl ether (MTBE)/water (4:92:4; v/v, eluent B). A gradient elution scheme was applied, commencing at 0% B and increasing to 58% B within a duration of 40 min. Subsequently, followed by a rapid transition to 100% B was affected over 1 min, sustained for 5 min, and ending with a return to 0% B over 9 min. The entire chromatographic analysis transpired over a period of 55 min, employing a fixed injection volume of 10 µL. Detection of tocopherol isomers occurred at specific wavelengths, and their respective concentrations were ascertained via the utilization of calibration curves.

### PSO preparation for FRAP, total phenolic, and total flavonoid assays

To determine the ferric reducing antioxidant power (FRAP), total phenolic content, and total flavonoid content, a methanolic extract of cold-pressed PSO was prepared [38]. Approximately 10 g of PSO was mixed with 20 mL of 80% (v/v) methanol in water. The mixture was vortexed for 10 min and then centrifuged at 3000 × g for 10 minutes to separate the phases. The methanolic phase was carefully collected and stored at -20°C until further analysis. This extraction was performed in triplicate.

### Total phenolic content and total flavonoid content of PSO

The total phenolic content in PSO was determined employing the Folin–Ciocalteu (F-C) method. In this process, 0.2 mL of the PSO methanolic extract was mixed with F-C reagent and sodium carbonate, subjected to incubation, and the absorbance was spectrophotometrically determined at 760 nm [39]. The total phenolic content was subsequently calculated and expressed as milligrams of gallic acid equivalents per gram (GAE/g) of the oil. For total flavonoid content, the PSO methanolic extract was mixed with a methanolic solution of aluminum chloride, incubated, and measured at 430 nm. The quantification of the flavonoid content was then determined and reported as milligrams of rutin equivalents (RE) per gram of the oil [40].

### Determination of antioxidant capacity of PSO

The antioxidant potential of PSO was assessed through the application of two distinct methodologies, namely the oxygen radical absorbance capacity (ORAC) and FRAP assays, following established methods [41, 42]. In the ORAC assay, PSO extract was resuspended in acetone and diluted with 7% randomly methylated β-cyclodextrin (RMCD) [43]. The mixture, along with a Trolox standard, was combined with a fluorescein reagent and an AAPH solution. Fluorescence was monitored over a period of 2.5 h, and the outcomes were quantified as micromoles of Trolox equivalent per gram of oil (µmol TE/g). For the FRAP assay, PSO methanolic extract was mixed with a freshly prepared FRAP reagent and subsequently diluted. Following a 10 min incubation and subsequent centrifugation, the absorbance at 593 nm was recorded against a reagent blank.

### Ethics statement

This study was approved by the Siriraj Animal Care and Use Committee (SiACUC), Faculty of Medicine Siriraj Hospital, Mahidol University (Bangkok, Thailand), under COA number 015/2563 and Protocol number 004/2563. The experiments were conducted in compliance with institutional, national, and international guidelines, including the International Guiding Principles for Biomedical Research Involving Animals, and UKCCCR guidelines for the welfare of animals in experimental neoplasia. Informed consent was not applicable, as this study involved animals. Throughout the study, all animals were housed under controlled conditions and monitored daily by veterinary officers and staff at the Siriraj Laboratory Animal Research and Care Centre. Humane endpoints, such as weight loss exceeding 15%, sustained rectal haemorrhage, rectal prolapse, and severe lethargy with poor grooming, were established to minimize suffering and ensure animal welfare.

### Experimental animals

In this investigation, 108 Jcl:ICR male mice, aged four weeks with an average weight of 18–22 g, were procured from Nomura Siam International Co., Ltd. (Bangkok, Thailand). These mice were accommodated at the Siriraj Laboratory Animal Research and Care Centre, Faculty of Medicine Siriraj Hospital, Mahidol University (Bangkok, Thailand), under controlled conditions: 22±2°C, 50–70% relative humidity, a 12:12 h light/dark cycle, and *ad libitum* access to food and water.

### Experimental diet formulation

The animal diet employed in this study was a modified iteration of the AIN-93M rodent diet designed for maintenance according to Reeves [44] (Table 1). The standard control diet utilized 5% (w/w) soybean oil as the primary fat source instead of the original 4%. The experimental diet, centred on PSO, manifested three distinct formulations: a low dose variant involving the substitution of 5% (w/w) soybean oil with an equivalent quantity of PSO, a middle dose configuration, entailing the 5% (w/w) soybean oil and 5% (w/w) cornstarch with 10% (w/w) PSO, and a high dose replacing 5% (w/w) soybean oil and 15% (w/w) cornstarch with 20% (w/w) PSO. Furthermore, a diet incorporating fish oil was implemented, replacing 5% (w/w) soybean oil and 5% (w/w) cornstarch with 10% (w/w) fish oil. This incorporation of fish oil was based on prior reports indicating its inhibitory effect on colorectal carcinogenesis in a mouse model [45, 46].

**Table 1:** The composition of the experimental diets modified based on the AIN-93M diet.

Ingredients (g/kg)	Control	PSO	PSO	PSO	Fish oil
		(5%)	(10%)	(20%)	(10%)
Cornstarch	456	456	406	306	406
Casein (>85% protein)	140	140	140	140	140
Dextrinized cornstarch	155	155	155	155	155
Sucrose	100	100	100	100	100
**Soybean oil**	**50**	**-**	**-**	**-**	**-**
**Perilla seed oil**	**-**	**50**	**100**	**200**	**-**
**Fish oil**	**-**	**-**	**-**	**-**	**100**
Fiber	50	50	50	50	50
Mineral mix	35	35	35	35	35
Vitamin mix	10	10	10	10	10
L-Cystine	2	2	2	2	2
Choline bitartrate	3	3	3	3	3
Tert-butylhydroquinone	0.008	0.008	0.008	0.008	0.008
**Total**	**1000**	**1000**	**1000**	**1000**	**1000**
**Total energy (kcal)**	**3853**	**3853**	**4103**	**4603**	**4103**
%Energy from carb	73.79	73.79	64.41	48.73	64.41
%Energy from protein	14.53	14.53	13.65	12.16	13.65
%Energy from fat	11.68	11.68	21.94	39.11	21.94
**%Total**	**100**	**100**	**100**	**100**	**100**

### Carcinogen preparation

In order to induce colon carcinogenesis in mice, a combination of AOM and DSS was employed. The AOM stock solution (10 mg/mL) was prepared by diluting 100 mg of AOM (Merck KGaA, Darmstadt, Germany) in 10 mL of sterile water and subsequently stored at -20°C. The AOM working solution was then made by resuspending 1 mL of the stock solution in 9 mL of sterile 0.9% normal saline, resulting in a final concentration of 1 mg/mL. Subsequently, the injection volume for the mice was adjusted to 10 mg/kg body weight based on their individual weights. Concurrently, the DSS solution was formulated by dissolving 1 gram of DSS (TdB Labs, Uppsala, Sweden) in 100 mL of sterile water to yield a 1% (w/v) solution. This DSS solution was freshly prepared and renewed daily to maintain its efficacy. All experimental procedures were meticulously executed within the confines of a fume hood to ensure safety and adherence to laboratory protocols.

### Experimental design

**Fig 1:**
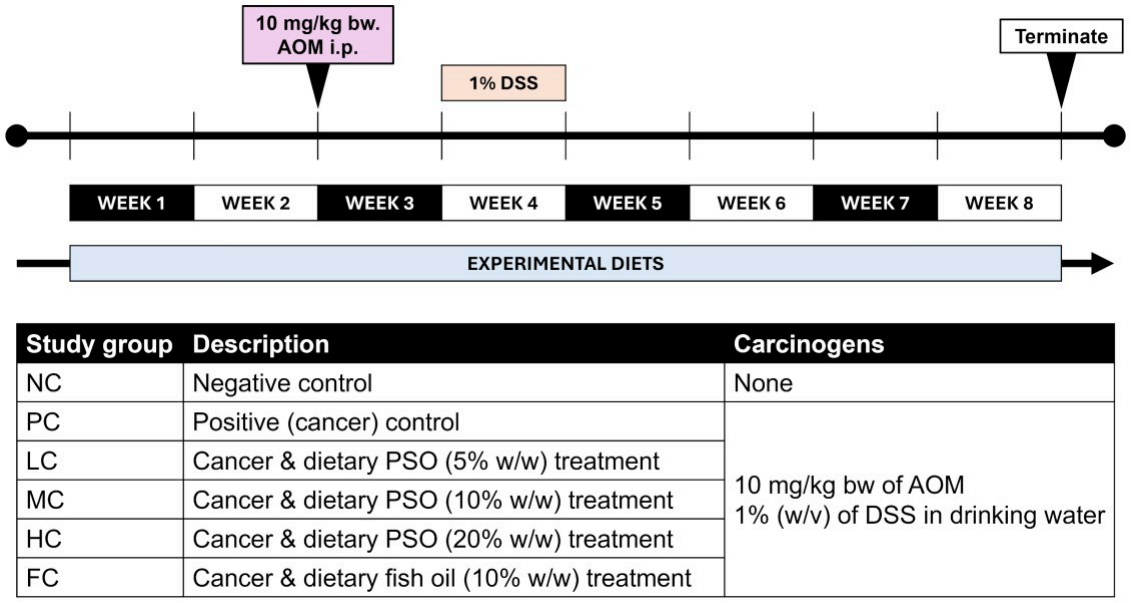
Schematic diagram of experimental design.

A total of 108 mice were stratified into six groups, each comprising 18 mice. The negative control (NC) group was administered a standard diet without carcinogen treatment. The positive control (PC) group was induced with AOM and DSS to promote CRC development while receiving a standard diet. Three groups were given perilla oil in varying concentrations: the low-dose perilla oil diet group (LC) received 5% (w/w) PSO, the middle-dose perilla oil diet group (MC) received 10% (w/w) PSO, and the high-dose perilla oil diet group (HC) received 20% (w/w) PSO, all concurrently with AOM and DSS treatment. The fish oil diet treatment group (FC) was also induced with AOM and DSS, receiving a 10% (w/w) fish oil diet. After one week of acclimatization, the mice were maintained on their respective dietary regimens throughout the experimental period. Colitis-associated CRC was induced based on the protocols of Tanaka et al. [47] and Suzuki et al. [48, 49]. On day 15, all mice, except the NC group, received an intraperitoneal injection of 10 mg/kg AOM, while the NC group received 0.9% saline. From days 22 to 28, all mice except the NC group were given 1% (w/v) DSS in their drinking water. Continuous monitoring of body weight, food, and water consumption were conducted. On day 57, mice were euthanised with isoflurane overdose for blood and organ collection. The experimental timeline is depicted in Fig 1.

### Colitis assessment in mice

The clinical manifestations of colitis-associated CRC in mice were systematically observed on a daily basis subsequent to the administration of AOM. Disease progression and severity were assessed using the disease activity index (DAI). The evaluation encompassed a comprehensive analysis of the general appearance, behavioural alterations, a typical weight reduction, stool characteristics, and the presence of occult blood or pathological bleeding from the anus in each mouse, with corresponding records and scoring. The criteria for DAI scoring, which were described elsewhere [50, 51], are delineated in Table 2. The DAI score was derived by aggregating the individual scores for weight loss, stool consistency, and haemorrhage, and subsequently dividing the sum by three for a comprehensive assessment of the overall disease activity index.

**Table 2:** The criteria for scoring the disease activity index (DAI).

Score	Weight loss (%)	Stool consistency	Rectal bleeding
**0**	None	Normal	Negative
**1**	0–4.99	Loose/Pasty stool	Negative
**2**	5–9.99	Loose/Pasty stool	Positive for occult blood
**3**	10–14.99	Diarrhoea	Positive for occult blood
**4**	15–20	Diarrhoea	Gross rectal bleeding

### Animal welfare monitoring

Throughout the experiment, autoclaved cages, along with bedding materials (corn cob and dried water hyacinth), were replaced weekly. Sterile bedding sheets were added to each cage to serve as enrichment, reducing distress and discomfort, while also facilitating the detection of gastrointestinal abnormalities. Mice displaying signs of dehydration, such as a 5–10% weight loss within 24–48 hours, diarrhoea, or rectal haemorrhage, received immediate fluid replacement therapy. The required fluid volume for each dehydrated mouse was assessed and calculated by institutional veterinary staff using the following formulae. Dehydrated mice were administered warm Lactated Ringer’s Solution (LRS) intraperitoneally and subcutaneously, a combination of physiological saline solution and electrolytes mimicking extracellular fluids. The 24-hour maintenance volume was divided into three small doses administered at 6-hour intervals, or until the animal’s weight rebounded, typically within 2–3 days post-emergency fluid correction. Regarding tumour burden, although internal orthotopic neoplasm determination was challenging, any clinical signs of AOM/DSS-mediated CRC in mice were diligently monitored daily throughout the experiment as surrogate markers for disease progression. According to the UKCCCR guidelines for the welfare of animals in experimental neoplasia [52, 53], humane endpoints were provided to animals before they deteriorated or faced predictable demise, which defined as significant weight loss exceeding 15%, sustained rectal haemorrhage over consecutive days, rectal prolapse, and severe lethargy with poor grooming [54]. No surgical procedures were performed on mice in this study. All efforts and experimental designs were made to minimise suffering and casualties, ensuring strict maintenance of animal well-being.


$$Emergency\;correction\;volume\;(mL)=Body\;weight\;\left(gram\right)\times Dehydration\;(\%decimal)$$

$$24-hour\;Maintenance\;volume\;\left(mL\right)=100\;mL/kg/day$$

### Necropsy and specimen collection

In each study group (n = 18 mice per group), following euthanasia using isoflurane, whole blood was collected via cardiac puncture and processed to isolate serum. The entire colon, including the caecum, was harvested post-dissection and photographed for length measurement. The caecum was collected and stored at -80°C, with 12 out of 18 caecum samples being used for subsequent gut microbiota analysis. For the colon, after removal of luminal contents and photographing the mucosal surface, the samples were divided for further analysis. Six colon samples from each group were fixed in paraformaldehyde and preserved in 70% ethanol for histopathological evaluations. These six samples underwent alcian blue staining for mucin-depleted foci (MDF) evaluation, followed by methylene blue staining for aberrant crypt foci (ACF) evaluation. After gross pathology, the same six samples were processed for paraffin embedding and sectioning for histopathological analysis, including haematoxylin and eosin (H&E) staining for dysplasia grading and immunohistochemistry staining for β-catenin, proliferating cell nuclear antigen (PCNA), and cyclin D1. The remaining 12 colon samples per group were promptly frozen at -80°C for biochemical analysis. Of these, two samples per group were used to optimize the western blotting protocol. Once the optimal conditions were established, 10 samples per group were used for the final western blot analysis to assess CRC biomarkers.

### Gross pathological examination of the colon

The mouse colon underwent a gross pathological examination, commencing with the measurement of its length during necropsy. Subsequently, the entire colon image was meticulously scrutinized using Fiji image analysis software to obtain precise results. For the evaluation of tumour burden, the colon, which had been previously fixed in 70% ethyl alcohol, underwent thorough rinsing, was evenly segmented into proximal, middle, and distal parts, and subjected to alcian blue staining. Following staining and rinsing to remove excess dye, the colon mucosa was observed under low magnification, and mucosal images were captured and subjected to analyses using Fiji image analysis software [55]. Elevated lesions, such as polyps and adenomas, were quantified, and the area occupied by these lesions was measured to calculate tumour volume or burden. Additionally, tumour incidence and multiplicity (number of tumours per mouse) were determined.

### Determination of preneoplastic lesions in mouse colons

Preneoplastic lesions were assessed in mouse colons through the assessment of both MDF and ACF. The MDF evaluation employed conventional alcian blue staining, as outlined by Yoshimi et al. [56], whereby alcian blue was administered to the colon mucosa. Scarce or absent mucus-producing cells were identified and counted under a light microscope [57], and the findings were documented. The ACF assessment encompassed the application of methylene blue staining to the unsectioned colon sample [58], followed by microscopic examination. Aberrant crypts, characterized by enlarged size and other distinct features [59], were identified, classified into small, medium, or large foci [60], and quantified using Fiji image analysis software. The analysed colon mucosa was preserved in 70% ethanol for subsequent investigations.

### Histopathological analysis of mouse colons

Colon tissues underwent processing, sectioning, and subsequent staining with H&E. Dysplasia, serving as an indicator for abnormal cell growth and potential cancer risk [61, 62], was assessed in the H&E-stained colon samples. Dysplasia was graded into three types: 1) atypia or hyperplasia without dysplasia, characterized by increased cell numbers and normal cell features; 2) mild to moderate dysplasia or low-grade dysplasia, featuring elongated crypts, reduced goblet cells, mild cell differentiation, and altered nuclear characteristics; 3) moderate to severe dysplasia or high-grade dysplasia, displaying severe crypt malformation, lack of goblet cells, poor differentiation, abnormal nuclei, high nucleus-to-cytoplasm ratio, and numerous mitotic figures [63–65]. The assessment of dysplasia was conducted on H&E-stained sections using a microscope and quantified as a percentage of total dysplasia through the utilisation of QuPath software [66].

### Immunohistochemical staining

Colon tissue sections were processed for immunohistochemistry (IHC) staining, beginning with heating the sections at 60°C, followed by deparaffinization in xylene and rehydration through a graded ethanol series. Heat-induced epitope retrieval was performed in sodium citrate buffer, after which the sections were treated with hydrogen peroxide to block endogenous peroxidase activity and permeabilized with Triton-X 100. Blocking was carried out using bovine serum albumin (BSA) to minimize non-specific binding. The sections were then incubated overnight with primary antibodies: Anti-beta Catenin antibody [E247]—ChIP Grade (Abcam, Boston, MA, USA), Anti-Cyclin D1 antibody [SP4] (Abcam, Boston, MA, USA), and PCNA (D3H8P) XP^®^ Rabbit mAb #13110 (Cell Signaling Technology, Inc., Danvers, MA, USA). Following thorough washing, the sections were incubated with a biotinylated secondary antibody, Goat Anti-Rabbit IgG (H+L) (BA-1000-1.5) (Vector Laboratories, Inc., Newark, CA, USA), and subsequently treated with the VECTASTAIN^®^ ABC-HRP Kit (PK-4000) (Vector Laboratories, Inc., Newark, CA, USA). Signal detection was carried out using DAB (3,3’-diaminobenzidine) as the chromogen (DAB Substrate Kit, Peroxidase (HRP), with Nickel, SK-4100, Vector Laboratories, Inc., Newark, CA, USA), and the sections were counterstained with Carazzi’s haematoxylin. After dehydration and clearing, the sections were mounted with coverslips. IHC staining intensity was assessed using the modified histochemical score (H-score), a semiquantitative measurement tool analysed through QuPath software [66]. The H-score, ranging from 0 to 300, is calculated from the staining intensity and the percentage of IHC-positive cells. Staining intensity was scored on a scale of 0–3, where 0 represents negative staining, 1 weak, 2 moderate, and 3 strong staining, respectively [67–69].

### Western blot analysis

Frozen colon tissue samples were thawed, and protein lysates were prepared utilising RIPA buffer containing protease and phosphatase inhibitors. Protein concentrations were ascertained using the Bradford assay. Samples were then mixed with β-mercaptoethanol and Laemmli Sample Buffer, followed by separation via SDS-PAGE. Proteins were transferred to PVDF membranes (Immun-Blot^®^ PVDF Membrane #1620177, Bio-Rad Laboratories, Inc., Hercules, CA, USA). Membranes were blocked with non-fat dry milk to prevent non-specific binding and incubated with primary rabbit monoclonal antibodies: Anti-beta Catenin antibody [E247]—ChIP Grade (Abcam, Boston, MA, USA), Cox2 (D5H5) XP^®^ Rabbit mAb #12282 (Cell Signaling Technology, Inc., Danvers, MA, USA), Phospho-NF-κB p65 (Ser536) (93H1) Rabbit mAb #3033 (Cell Signaling Technology, Inc., Danvers, MA, USA), β-Actin (13E5) Rabbit mAb #4970 (Cell Signaling Technology, Inc., Danvers, MA, USA), and Anti-Ras antibody [EPR3255] (Abcam, Boston, MA, USA). After washing, membranes were incubated with the secondary antibody, Anti-mouse IgG, HRP-linked Antibody #7076 (Cell Signaling Technology, Inc., Danvers, MA, USA). Protein bands were visualised using DAB substrate (ImmPACT^®^ DAB Substrate Kit, Peroxidase (HRP), SK-4105, Vector Laboratories, Inc., Newark, CA, USA), scanned and analysed using Fiji software. Protein expression levels were quantified by calculating the ratio of the target protein to β-actin, which was used as the reference protein.

### Intestinal microflora determination

Mouse caecal stool samples, collected post-necropsy, were subjected to thawing and extraction procedures employing the QIAamp PowerFecal Pro DNA Kit (QIAGEN GmbH, Germany), following the manufacturer’s instructions. Subsequent steps included amplifying and pyrosequencing 16S ribosomal DNA (rDNA) sequences on an Illumina MiSeq platform. Taxonomic assignment was achieved by grouping sequence reads into operational taxonomic units (OTUs) at a 97% sequence similarity level. To assess the relative abundance of bacterial taxa between groups, 16S rRNA gene-targeted RT-PCR was performed. Microbiome bioinformatics was conducted using QIIME 2 2022.2 [70]. Raw sequence data underwent demultiplexing via the q2‐demux plugin. Reads with expected errors (maxEE) exceeding 3.0 were removed by denoising software DADA2 (via q2‐dada2), and chloroplast-related 16S sequences were excluded. A phylogenetic tree was built from representative sequences with the q2-phylogeny plugin’s align_to_tree_mafft_fasttree action. For diversity analysis, alpha‐diversity and beta-diversity metrics, along with Principal Coordinate Analysis (PCoA), were estimated using q2‐diversity after subsampling to 12,270 reads. Taxonomy was assigned to ASVs using the classify‐sklearn naive Bayes taxonomy classifier against Greengenes 13_8 99% OTUs reference sequences. Statistical assessments for alpha and beta diversity utilised Kruskal-Wallis and PERMANOVA (number of permutations = 999) tests, respectively.

### Statistical analysis

Numerical data were subjected to ANOVA, with application of Tukey or Dunnett T3 correction for multiple comparisons in cases where the data demonstrated a normal distribution. For non-normally distributed data, the Kruskal-Wallis test was employed followed by Dunn’s correction for multiple comparisons. The analysis was conducted using GraphPad Prism software version 10.1.2 (324), and results are presented as mean ± SD or mean ± SEM as specified. Statistical significance was deemed at P < 0.05.

## Results

### Nutritive values and bioactivity of PSO

The present study involved the scrutiny of PSO for its nutritive composition and bioactive properties, as delineated in Table 3. The preeminent fatty acid discerned in the oil was ALA (C18:3n-3), constituting 61.32% of total fatty acid content. Subsequently, linoleic acid (C18:2n-6) was observed at 19.06%, and oleic acid (C18:1n-9) at 9.66%. Stearic acid, palmitic acid, and caprylic acid were also present, albeit in smaller proportions. The total tocopherol content amounted to 783.90 mg/kg oil, with the total phenolic and flavonoid contents measured at 23.46 mg GAE/g oil and 0.11 mg RE/g oil, respectively. The oil demonstrated notable antioxidant capabilities, evidenced by an ORAC value of 161.07 µmol TE/g and a FRAP value of 7.57 µmol TE/g. These findings emphasise the potential health benefits of PSO as a good source of n-3 fatty acids and bioactive compounds.

**Table 3:** Fatty acid composition, nutritive values, and antioxidant capacity of PSO.

**Fatty acid composition**	**Amount (%)**
Octanoic (caprylic)	0.04 ± 0.06
Hexadecanoic (Palmitic)	7.61 ± 0.48
Octadecanoic (Stearic)	2.31 ± 0.06
Cis-9-Octadecenoic (Oleic)	9.66 ± 2.80
Cis-9,12-Octadecadienoic (Linoleic)	19.06 ± 0.76
Cis-9,12,15-Octadecatrienoic (Linolenic)	61.32 ± 1.50
**Bioactive compound**	**Amount (unit)**
Total tocopherols	783.90 ± 10.35 mg/kg oil
Total polyphenols	23.46 ± 2.62 mg GAE/g oil
Total flavonoids	0.11 ± 0.02 mg RE/g oil
**Antioxidant capacity**	**Activity (µmol TE/g oil)**
Oxygen radical absorbance capacity (ORAC)	161.07 ± 6.94
Ferric reducing antioxidant power (FRAP)	7.57 ± 0.08

The data was expressed as mean ± SD. The analysis was performed in triplicate.

### General observation and DAI score

**Fig 2:**
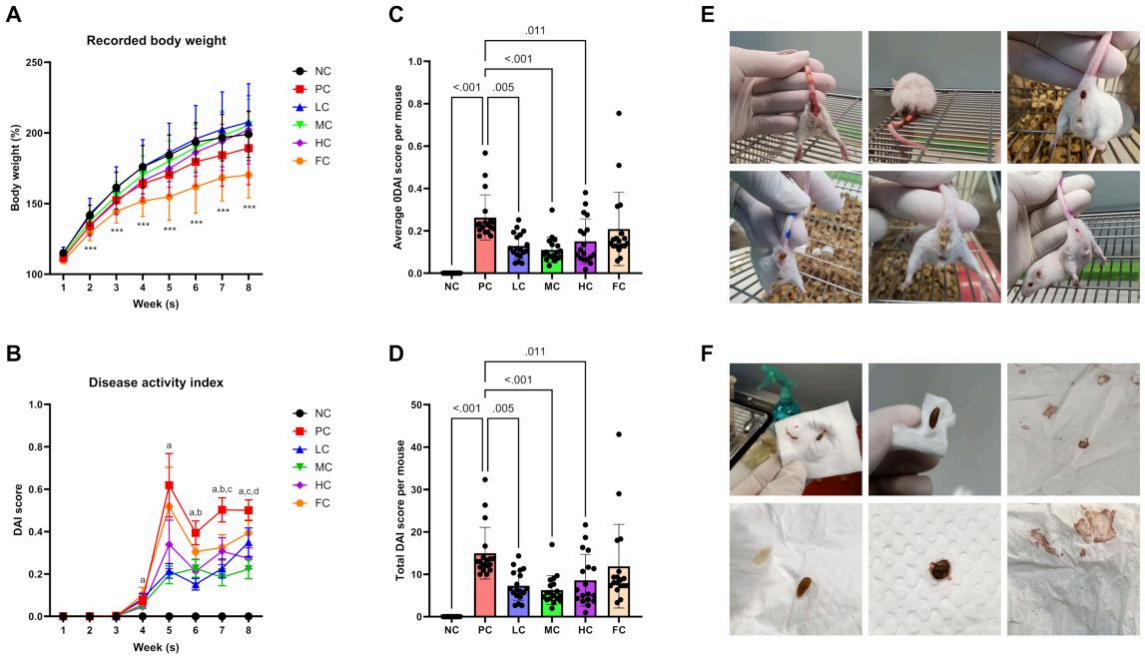
Body weight gain throughout the experiment **(A)**, analysed by one-way ANOVA with Tukey’s post-hoc correction (mean ± SD, n = 18). Disease Activity Index (DAI) scores at different time points **(B)**, average DAI score **(C)**, and total DAI score **(D)** for each group, analysed by Kruskal-Wallis tests followed by Dunn’s post-hoc correction (mean ± SEM, n = 18). Superscripts indicate significant comparisons: a = PC vs. NC, b = PC vs. LC, c = PC vs. MC, and d = PC vs. HC. Clinical manifestations of gastrointestinal discomfort, including diarrhoea and rectal haemorrhage, along with faecal pellets from AOM/DSS-induced mice **(E, F)**.

Throughout the experiment, the PC group exhibited significantly lower weight gain in comparison to their counterparts in the NC group during both the initial (P < 0.001) and second weeks (P = 0.041). The HC group demonstrated reduced weight gain only during the third week as opposed to the NC group (P = 0.049). Conversely, mice in the FC group consistently manifested reduced weight gain from the first week throughout the entire study (P < 0.001, vs NC), while exhibiting significantly lower weight gain compared to the PC group only at the conclusion of week 4 (P = 0.041), as depicted in Fig 2A. Upon general observation of mice subjected to AOM and DSS, discernible indications of gastrointestinal distress, such as weight loss, faecal blood, diarrhoea, and rectal bleeding, were evident as early as day 15 (Fig 2E and 2F). Disease activity index (DAI) scores were employed to assess the progression of the disease, with PC mice displaying significantly higher DAI scores compared to NC from week 4 onwards. The highest DAI score was observed in the PC group at week 5, registering a value of 0.62 ± 0.15 (mean ± SEM). The LC group demonstrated significantly lower DAI scores at week 6 (P = 0.014) and week 7 (P < 0.001) compared to the PC group, and both the MC and HC groups exhibited markedly reduced DAI scores at week 7 (P < 0.001 for MC and P = 0.037 for HC) and week 8 (P = 0.006 for MC and P = 0.041 for HC). No significant difference in DAI scores was observed between FC and PC groups throughout the study (Fig 2B). The overall DAI scores indicated that the PC group had the highest average (0.26 ± 0.11) and total DAI score (14.98 ± 6.10, mean ± SD), which were significantly higher than those in the NC (P < 0.001), LC (P = 0.005), MC (P < 0.001), and HC (P = 0.011) groups. However, no significant difference was found between the FC and PC group mice (Fig 2C and 2D).

### Tumour incidence, burden, and multiplicity

**Fig 3:**
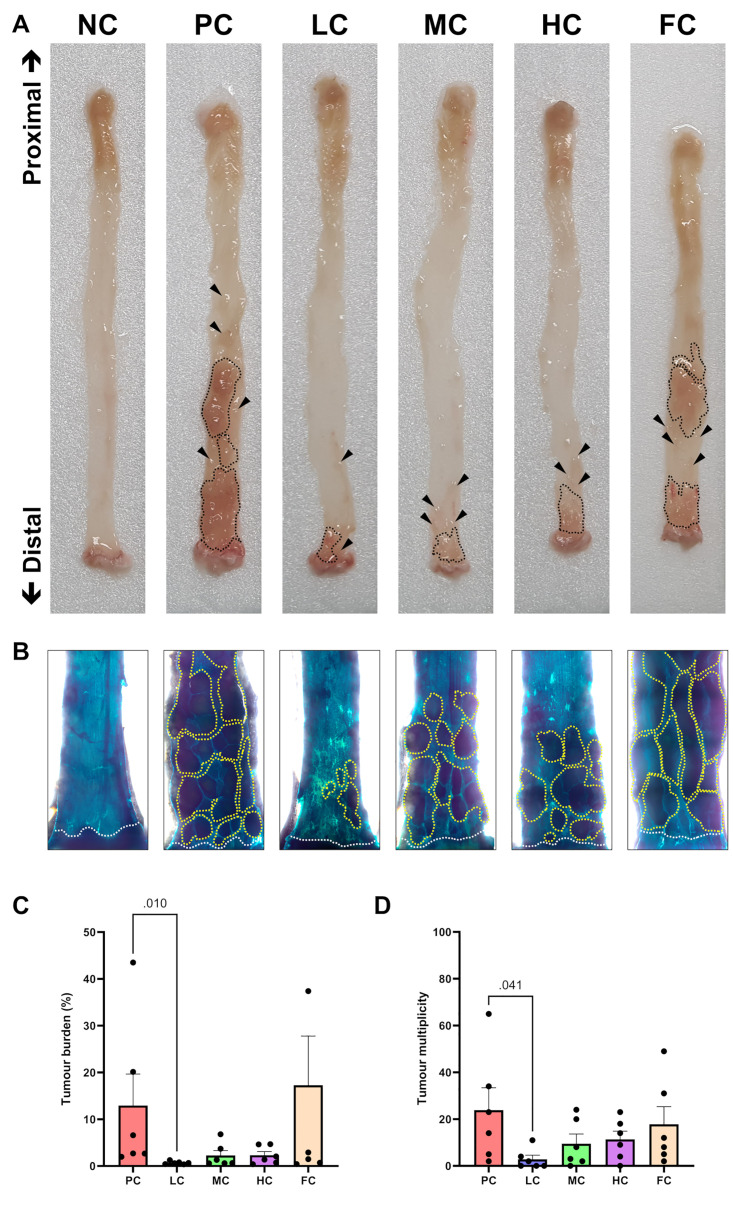
Gross mucosal lesions in mouse colons **(A)**, with black arrows and dotted lines indicating areas occupied by elevated lesions on the colon mucosa. Alcian blue-stained colonic mucosa micrographs showing tumour multiplicity at 12.5X magnification **(B)**, where white dotted lines represent the dentate lines (anorectal junction) and yellow dotted lines indicate adenomas and microadenomas on the colon mucosa. Tumour burden analysis **(C)** and tumour multiplicity **(D)** are expressed as mean ± SEM, and were analysed using Kruskal-Wallis tests followed by Dunn’s multiple comparisons test (n = 6).

In relation to tumour incidence, the NC group exhibited a 0% occurrence of colon tumours, as the induction with AOM or DSS was not administered. Conversely, among the groups subjected to AOM and DSS treatment, the PC and FC groups displayed a 100% tumour incidence. Notably, within the groups receiving a diet supplemented with PSO, the MC and HC groups manifested an 83.33% incidence, while the LC group exhibited the lowest incidence at 50%. The assessment of tumour burden, denoting the proportion of the colon mucosal area occupied by elevated lesions (depicted in Fig 3A), revealed the highest burden in the FC group, accounting for 17.28% of the total mucosal area. Following closely, the PC group exhibited a burden of 12.92%. In contrast, the HC and MC groups displayed lower tumour burdens at 2.30% and 2.26%, respectively. Remarkably, the LC group demonstrated the lowest tumour burden at 0.67%, significantly diverging from the PC group (P = 0.010), as illustrated in Fig 3C. Regarding tumour multiplicity (Fig 3B), the PC group presented the highest tumour count per mouse at 23.83, succeeded by the FC group at 17.83. The HC and MC groups displayed 11.33 and 9.50 tumours per mouse, respectively. Strikingly, the LC group showcased the lowest tumour count at 2.83, exhibiting a significant difference from the PC group (P = 0.041), as shown in Fig 3D.

### Preneoplastic lesions

**Fig 4:**
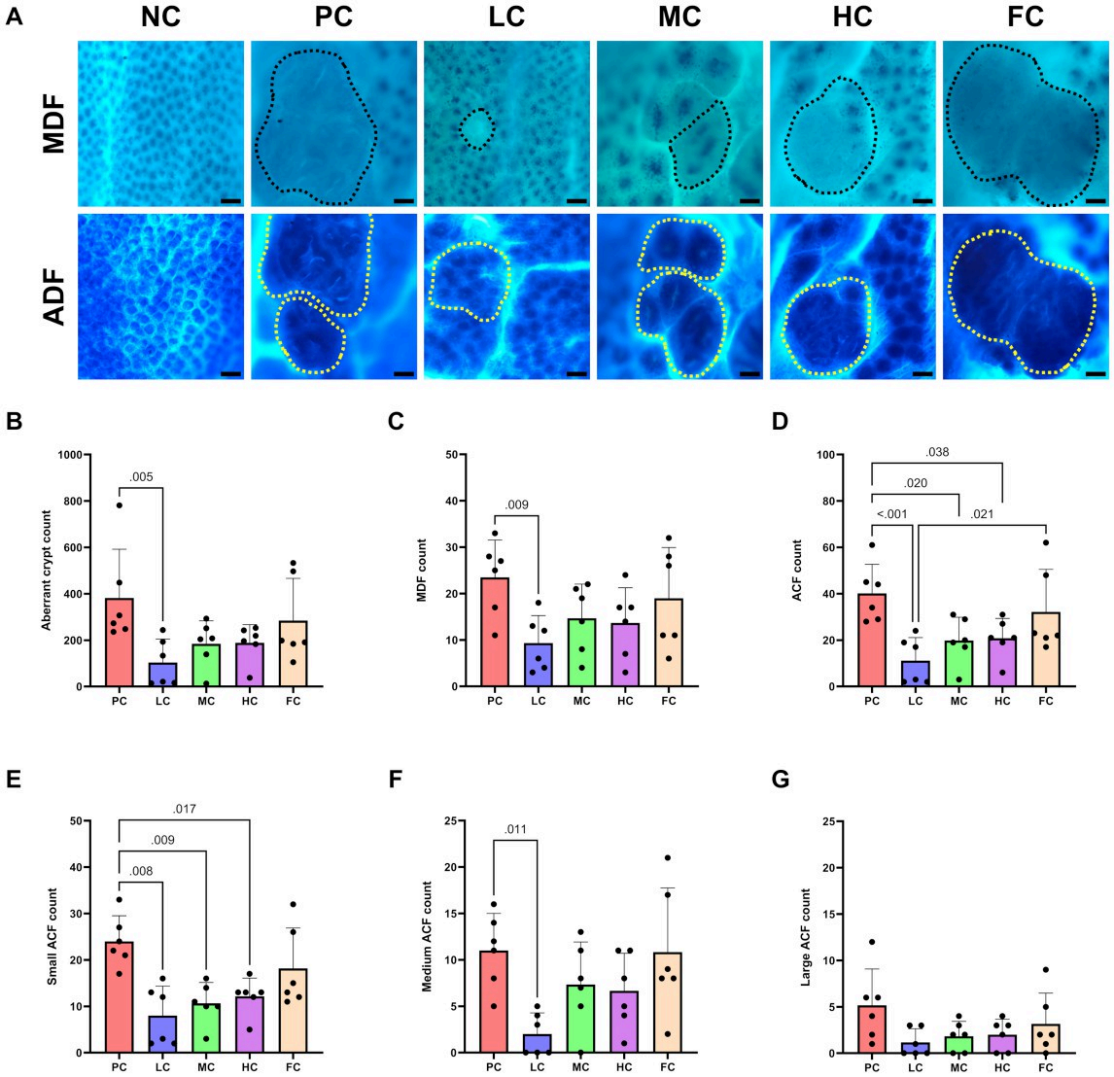
Topographic micrographs of colon mucosa stained with alcian blue and methylene blue at 100X magnification, with a 100 µm scale bar **(A)**. Black dotted lines indicate MDF, characterised by a lack or scarcity of mucus-producing cells, as visualized by reduced mucin staining. Yellow dotted lines represent ACF, characterised by elevated and enlarged crypts compared to adjacent cells, slit-like crypt openings, and an increased pericryptal area, indicated by darker blue-stained crypts. Aberrant crypt counts **(B)**, MDF counts **(C)**, and ACF counts **(D)** were analysed by Kruskal-Wallis test followed by Dunn’s multiple comparisons test (n = 6), presented as mean ± SEM. ACF were categorized by size: small **(E)**, medium **(F)**, and large **(G)**, with analysis performed using one-way ANOVA followed by Dunnett T3 multiple comparisons test (n = 6), presented as mean ± SD.

The aberrant crypts encompassed both MDF and ACF, which are preneoplastic lesions of CRC, as illustrated in Fig 4A. PC mice demonstrated the highest incidence of aberrant crypts, quantified at 382.17 ± 85.70, surpassing FC mice with 284.67 ± 74.26. Among mice subjected to a diet enriched with PSO, the HC and MC groups exhibited 188.50 ± 31.98 and 184.67 ± 40.25 aberrant crypts, respectively, whereas the LC group exhibited the lowest count at 103.50 ± 41.27, notably lower than that of PC mice (P = 0.005) (Fig 4B). The PC group recorded the highest MDF count (23.50 ± 3.28), followed by the FC group (19.00 ± 4.46). The MC and HC groups demonstrated a moderate number of MDF (14.67 ± 3.01 and 13.67 ± 3.09). The LC group presented the lowest MDF count (9.33 ± 2.42), significantly lower than PC mice (P = 0.009) (Fig 4C). In the ACF category, PC mice exhibited the highest count (40.17 ± 5.11), significantly surpassing the HC, MC, and LC groups (P = 0.038, 0.020, and < 0.001, respectively). FC mice had the second highest ACF count (32.17 ± 7.49), significantly exceeding the LC (P = 0.021) group, as illustrated in Fig 4D. Furthermore, ACF were categorized by size into small, medium, and large foci. PC mice showed the highest count of small ACF (24.00 ± 5.51), significantly surpassing the HC, MC, and LC groups (P < 0.05 for all three comparisons). FC mice recorded the second-highest count of small ACF, with the value of 18.17 ± 8.70. Medium-sized ACF were most prevalent in PC and FC groups (11.00 ± 4.00 and 10.83 ± 6.91), with the PC group showing significantly higher counts than the LC group (P = 0.011). Large-sized ACF exhibited similar patterns, with no significant differences observed between groups in this category (Fig 4E–4G).

### Dysplasia grading

**Fig 5:**
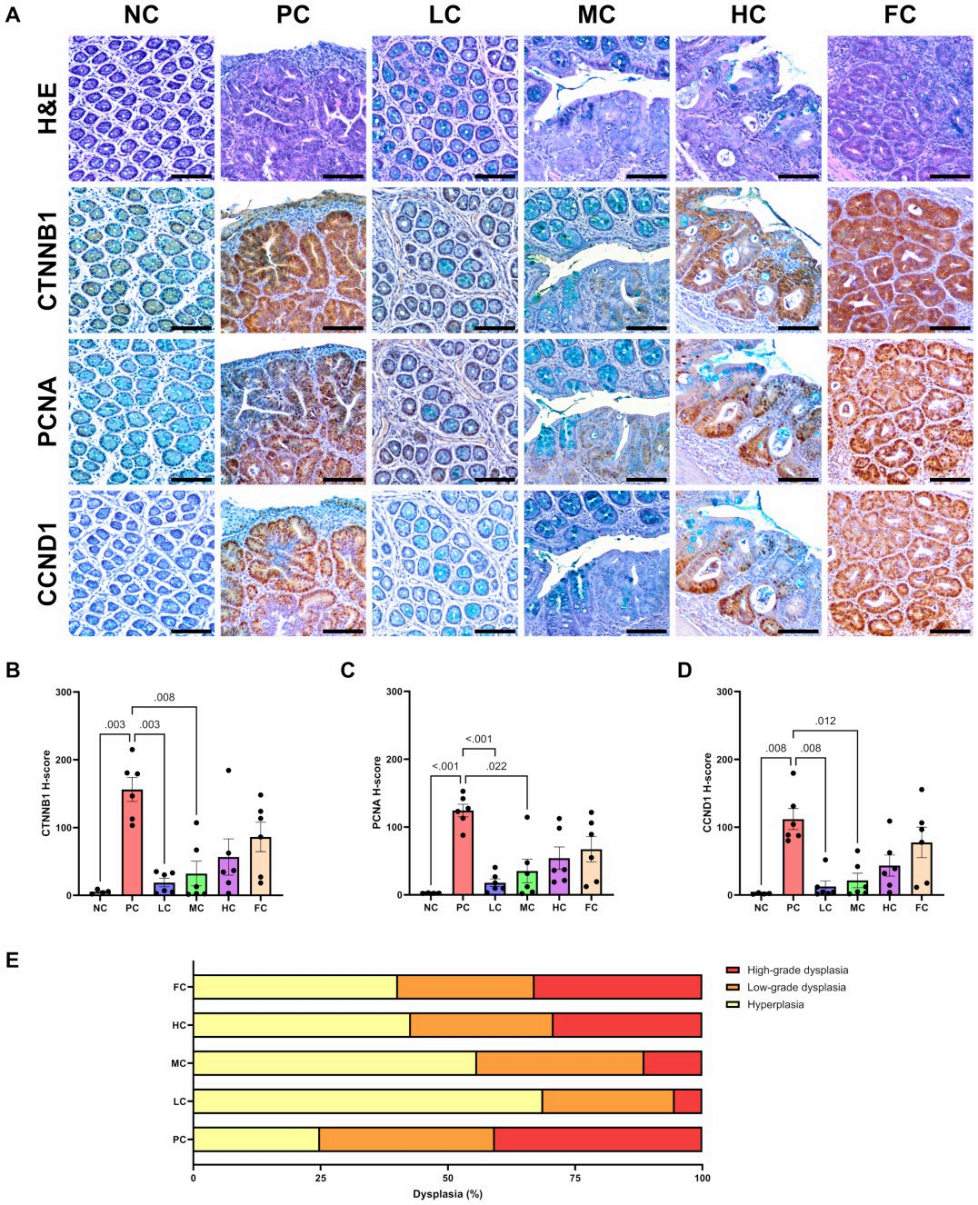
Micrographs of mouse colon mucosal cells stained with H&E and corresponding IHC serial sections at 200X magnification, with a 100 µm scale bar **(A)**. Dysplastic tissue shows poor cell differentiation, characterized by reduced goblet cells, distorted crypt architecture, and an increased nucleus-to-cytoplasm (N/C) ratio. β-catenin (CTNNB1) positive cells exhibit both cytoplasmic and nuclear IHC staining, while PCNA and cyclin D1 (CCND1) show only nuclear IHC staining. H-scores for CTNNB1 **(B)**, PCNA **(C)**, and CCND1 **(D)** immunopositive cells, with a maximum value of 300. Dysplasia grading in mouse colon mucosal cells for each group **(E)**. All data were analysed using One-way ANOVA followed by Dunnett T3 test for multiple comparisons correction, presented as mean ± SEM (n = 6).

The evaluation of dysplasia grading in colon tissues within the investigated groups revealed notable distinctions (Fig 5A H&E). High-grade dysplasia, indicative of moderate to severe dysplastic crypts, was most abundant in the PC group, constituting 40.74 ± 17.32% of the overall dysplastic tissues. This prevalence was markedly higher than observed in the MC and LC groups (P = 0.001 and P < 0.001, respectively). The FC group exhibited the second-highest incidence of high-grade dysplasia at 32.92 ± 8.38%, significantly surpassing the MC and LC groups (P = 0.035 and P = 0.003, respectively). Among the study groups, mice in the HC group mice displayed the third-highest occurrence of high-grade dysplastic tissues, accounting for 29.21 ± 12.41% of all colon dysplasia and exhibiting a statistically significant rise compared to the LC group (P = 0.015). While no statistically significant variations were observed in low-grade dysplasia across the study groups, the LC group demonstrated the highest prevalence of hyperplastic crypts, comprising 68.76 ± 18.97% of total dysplastic tissues. This incidence was significantly greater than that observed in the PC, HC, and FC groups (P < 0.001, P = 0.006, and P = 0.002, respectively). The MC group exhibited the second-highest prevalence of hyperplastic crypts at 55.77 ± 19.02%, significantly surpassing that of the PC group (P < 0.001), as depicted in Fig 5E.

### Immunohistochemistry

Immunohistochemistry revealed that within the PC group, the β-catenin (CTNNB1) exhibited the highest H-score, measuring 165.25 ± 17.69. This value was significantly higher compared to the NC, LC, and MC groups, with respective P-values of 0.003, 0.003, and 0.008. Conversely, the HC and FC groups displayed β-catenin H-scores of 56.56 ± 26.81 and 86.18 ± 21.67, respectively, and did not exhibit statistically significant differences between them. Similarly, the proliferating cell nuclear antigen (PCNA) exhibited the highest H-score in the PC group, registering at 124.35 ± 9.26. This score was significantly greater than those observed in the NC, LC, and MC groups (P < 0.001, < 0.001, and is 0.022, respectively). The HC and FC groups displayed PCNA H-scores of 54.00 ± 16.63 and 67.22 ± 18.63, respectively, with no discernible significant distinctions between them. Moreover, Cyclin D1 (CCND1) exhibited the highest expression in the PC group, presenting an H-score of 111.93 ± 15.46. This value was significantly higher in comparison to the NC, LC, and HC groups, with respective p-values of 0.008, 0.008, and 0.012. The HC and FC groups manifested CCND1 H-scores of 43.44 ± 15.52 and 77.54 ± 22.56, respectively, and did not demonstrate statistically significant differences (Fig 5A IHC and 5B–5D).

### Western blot

**Fig 6:**
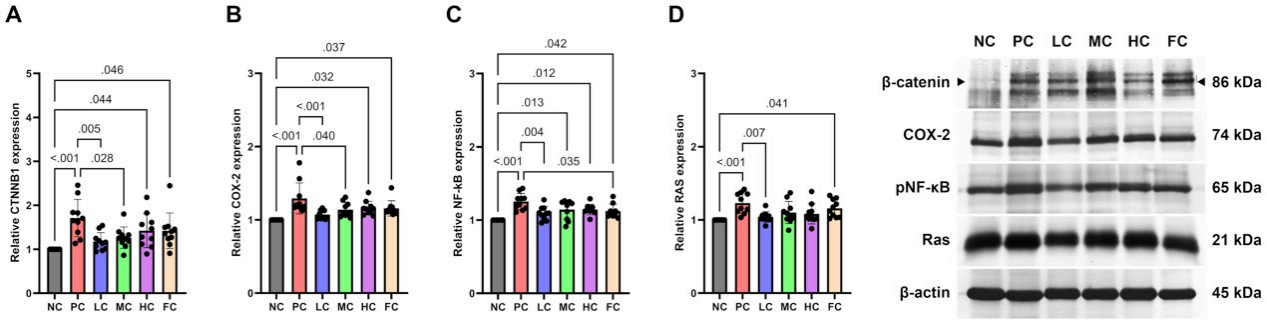
The relative protein expression levels of CTNNB1 **(A)**, COX-2 **(B)**, phosphorylated p65 NF-κB **(C)**, and RAS **(D)**. Data were analysed using One-way ANOVA with Tukey multiple comparisons correction and are presented as mean ± SD (n = 10).

In the context of Western blot analysis, discernible trends were observed among various experimental groups involving mice subjects. Specifically, mice subjected to the protocol in the PC group—wherein AOM and DSS were administered—evinced a notable upregulation in the expression levels of CTNNB1, COX-2, NF-κB (p65), and RAS proteins in comparison to counterparts in the NC group (P < 0.001 for all markers). Concomitantly, the FC group, characterized by a dietary regimen comprising 10% (w/w) fish oil, similarly manifested a substantial increase in the expression levels of CTNNB1, COX-2, NF-κB (p65), and RAS proteins as opposed to the NC group (P = 0.046, 0.037, 0.042, and 0.041, respectively), thereby the observations made in the PC group. Within the HC group, characterized by mice subjected to a diet formulated with 20% (w/w) perilla oil, a discernible elevation in the expression levels of CTNNB1, COX-2, and NF-κB (p65) proteins was evident when juxtaposed with the NC group (P = 0.044, 0.032, and 0.012, respectively). In the MC group, where mice were administered a 10% (w/w) perilla oil-formulated diet, an increase in NF-κB (p65) expression was noted in comparison to the untreated group (NC) (P = 0.013), while expressions of CTNNB1 and COX-2 were significantly reduced in contrast to the PC group (P = 0.028 and 0.040, respectively). Noteworthy findings arose from the LC group, wherein subjects received a 5% (w/w) perilla oil-formulated diet. Mice in this group demonstrated diminished expression levels of CTNNB1, COX-2, NF-κB (p65), and RAS relative to the PC group (P = 0.005, < 0.001, 0.004, and 0.007, respectively). A comprehensive visualization of these outcomes is succinctly presented in Fig 6A–6D.

### Gut microbial diversity

**Fig 7:**
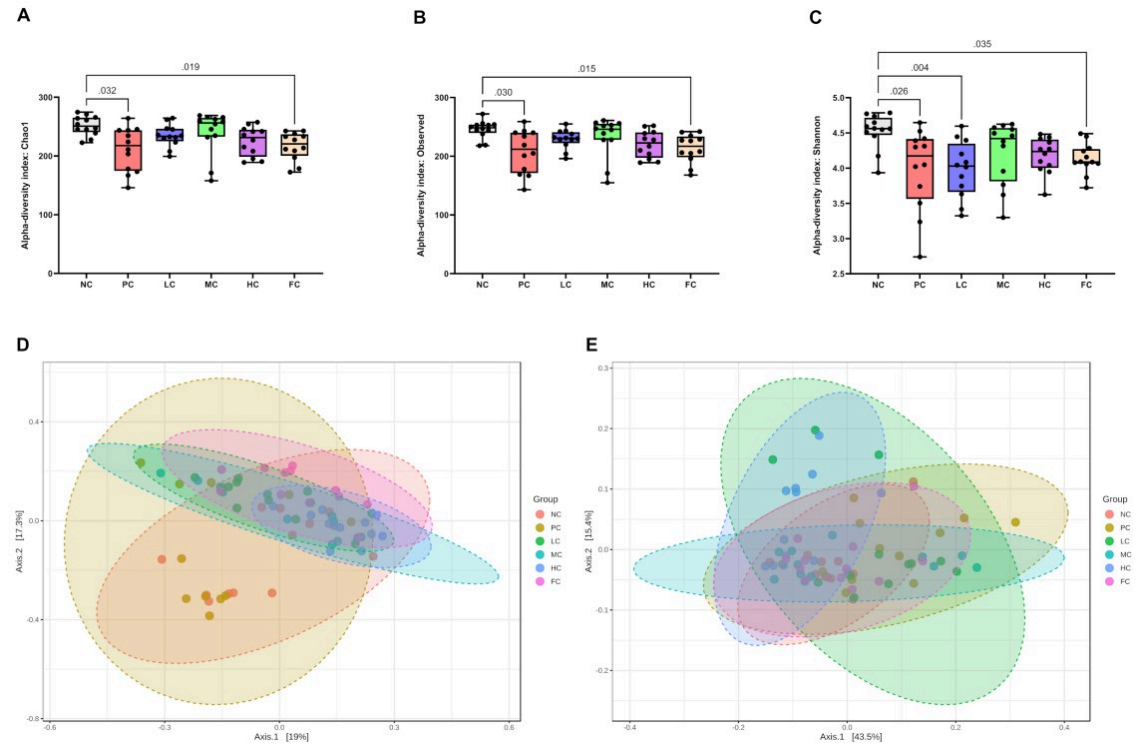
Alpha-diversity indices, including Chao1 **(A)**, Observed **(B)**, and Shannon **(C)** as well as PCoA plots for Bray-Curtis **(D)** and weighted UniFrac **(E)** beta-diversity metrics. Statistical tests for alpha and beta diversity utilized Kruskal-Wallis and PERMANOVA with 999 permutations, respectively (n = 12).

The intestinal microflora diversity was analysed using faecal content from caecum samples, focusing on both alpha- and beta-diversity within each study group. Alpha-diversity metrics, specifically the Chao1 index, Observed species, and Shannon index, were employed to evaluate species richness and evenness within the microbial community. Notably, both the PC and FC groups exhibited a noteworthy reduction in alpha-diversity when contrasted with the NC group (Fig 7A and 7B). Examining the even distribution of gut microbial species, the LC group mice, alongside the PC and FC groups, manifested reduced species evenness compared to their NC counterparts (Fig 7C). Beta-diversity was employed to investigate disparities in bacterial community composition between groups, utilizing Bray-Curtis dissimilarity and weighted UniFrac metrics. Mice subjected to AOM and DSS treatment displayed discernible distinctions in their gut microbiome relative to the NC group. Moreover, discernible variations were noted contingent upon the diverse dietary oil components incorporated in the formulated diets, as illustrated in Fig 7D and 7E.

### Gut bacterial taxonomic profiling

**Fig 8:**
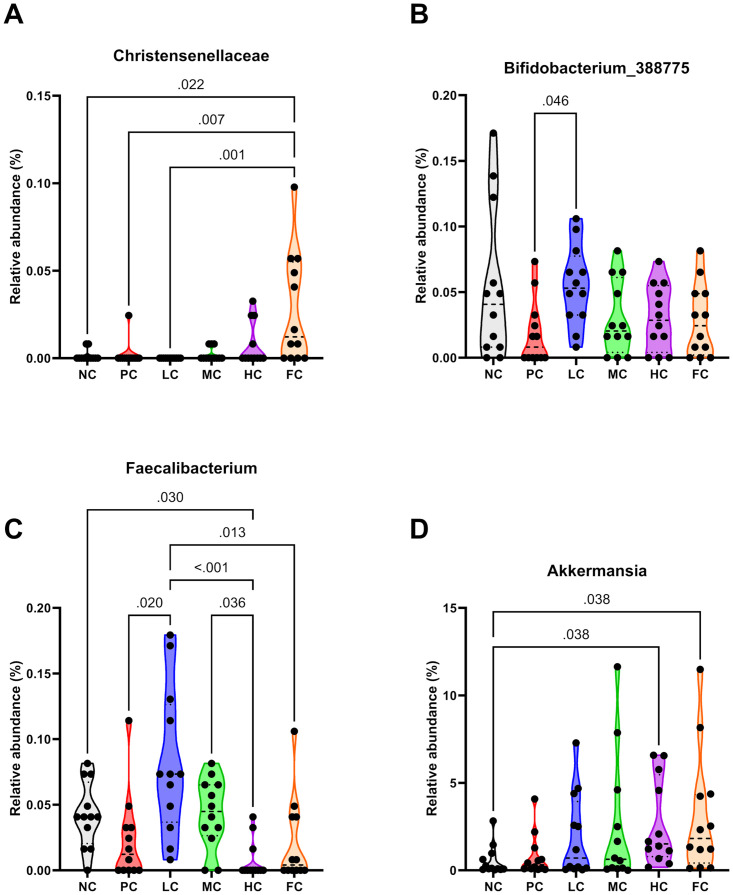
Violin plots for the relative abundances of the *Christensenellaceae* bacterial family **(A)** and the microbial genera *Bifidobacterium_388775* **(B)**, *Faecalibacterium* **(C)**, and *Akkermansia* **(D)**. Data were analysed using the Kruskal-Wallis test with Dunn’s correction for multiple comparisons (n = 12).

In the context of gut bacterial taxonomic profiling, the FC group exhibited a notably significantly higher abundance of the *Christensenellaceae* family in comparison to the NC (P = 0.022), PC (P = 0.007), and LC (P = 0.001) groups. Conversely, the LC group demonstrated a significantly higher prevalence of the *Bifidobacterium_388775* genus relative to the PC group (P = 0.046). Moreover, the *Faecalibacterium* genus exhibited a substantially augmented presence in mice of the LC group as opposed to those in the PC (P = 0.020), HC (P < 0.001), and FC (P = 0.013) groups. Conversely, mice in the HC group manifested a markedly lower abundance of the *Faecalibacterium* genus in the gut when compared to the NC (P = 0.030) and MC (P = 0.036) groups. Additionally, the *Akkermansia* genus displayed an increased prevalence in both the HC, and FC groups, in contrast to the NC group (P = 0.038 for both comparisons). A comprehensive visualization of these outcomes is presented in Fig 8A–8D.

## Discussion

PSO emerges as a substantial reservoir of ALA, constituting approximately 61.32% of its total fatty acid composition. This places PSO among the preeminent sources of ALA within the spectrum of diverse plant-derived alternatives. Our comprehensive analysis further elucidates a noteworthy total tocopherol content of 783.9 mg/kg oil, thereby enhancing its nutritional profile. These findings align with prior research, underscoring parallel ALA levels and substantial antioxidant capacities across various PSO types. Such studies attest to the consistent presence of ALA, with recorded values ranging from 53.14% to 60.39%, while tocopherol content exhibits variability contingent upon processing conditions. Moreover, the comparatively lower phenolic and flavonoid content in PSO does not detract from its considerable antioxidant capacity, which is presumably ascribed to its abundance in tocopherols [21, 22, 71–73]. Cumulatively, these findings accentuate the potential health benefits associated with PSO, underscoring its pivotal role as a wellspring of plant-derived n-3 polyunsaturated fatty acids and tocopherols within the realm of human nutrition.

All mice induced with both AOM and DSS manifested gastrointestinal disturbances, characterized by pronounced severe weight loss, diarrhoea, and rectal bleeding, as evidenced by elevated DAI scores. The group subjected to a standard diet (PC group) exhibited the highest cumulative DAI scores, followed by those administered a diet comprising 10% (w/w) fish oil (FC group). Conversely, the LC, MC, and HC groups, receiving diets enriched with 5% (w/w), 10% (w/w), and 20% (w/w) PSO, respectively, demonstrated significantly reduced DAI scores, suggestive of a mitigated disease severity. Interestingly, the FC group did not display statistically significant variations in DAI scores, implying that PSO, rather than fish oil, mitigated the deleterious effects induced by AOM/DSS-triggered colitis in mice. These outcomes are in concordance with prior investigations indicating that perilla oil reduces DAI scores and histopathological indices in colitis mice models induced by a high-fat diet [74]. This aligns with observed anti-inflammatory effects in DSS-induced colitis mice [75, 76]. Notably, our study deviates from certain reports illustrating the favourable impacts of fish oil in colitis mice models, as we did not observe a positive influence in mice subjected to a diet containing 10% (w/w) fish oil [74, 76].

During our investigation into the tumour inhibitory effects of PSO, we have successfully implemented a colitis-associated CRC model in ICR mice. This was achieved through the modification of an established protocol, utilizing a 1% (w/v) concentration of DSS instead of 2% (w/v) [47–49]. This adjustment not only ensured a 100% tumour incidence but also contributed to an improved welfare profile for the experimental animals. The induction of CRC was initiated by intraperitoneal administration of 10 mg/kg AOM, followed by a single one-week cycle of 1% (w/v) DSS in the drinking water. This approach yielded a consistently reproducible model. Our investigation revealed that mice subjected to AOM/DSS-induced developed colon tumours, with the highest incidence in the PC and FC groups (100%), and a comparatively lower incidence in the LC group (50%). Remarkably, dietary supplementation with 5% (w/w) PSO exhibited the most significant inhibitory effect on colon tumours. In contrast, higher concentrations of PSO (10% and 20% (w/w) demonstrated diminishing efficacy. Analysis of tumour burden metrics, including tumour occupancy and adenoma count, indicated that PC and FC group mice displayed the highest values, while LC group mice exhibited a significant reduction in both parameters. Interestingly, a higher abundance of high-grade dysplastic tissues was noted in PC, HC, and FC group mice. This abundance was significantly reduced by diets containing 5% and 10% (w/w) PSO, with the 5% (w/w) proving to be the most effective in mitigating high-grade dysplasia. These findings align with previous studies demonstrating the suppressive effect of PSO on colon tumorigenesis across various animal models [29–33].

The anti-proliferative potential of PSO, particularly attributed to its high ALA content, was discerned in our investigative study. IHC analysis unveiled that mice induced with AOM/DSS-triggered colitis, particularly within the PC group, manifested increased expression of proliferative markers in colon mucosal cells. In contrast, the LC and MC groups, subjected to diets containing 5% (w/w) and 10% (w/w) perilla oil, respectively, exhibited a discernible reduction in these markers. This observation implies a tangible anti-proliferative impact of PSO in the context of colitis-associated CRC. Previous research has ascertained the anti-cancer properties of ALA-rich PSO, with a particular emphasis on its influence on the expression of cell cycle-associated proteins. Specifically, ALA has been documented to impede cell proliferation across diverse cancer cell lines, encompassing those of breast and colon origins. Furthermore, evidence indicates ALA’s capability to diminish tumour size in animal models, underscoring its therapeutic potential. Notably, ALA appears to modulate the expression of pivotal proteins such as cyclin D1, thereby intricately regulating cellular growth dynamics [17–20].

Our western blot analysis elucidated increased levels of colon carcinogenic and inflammatory biomarkers in AOM/DSS-induced mice, particularly evident in the PC, HC, and FC groups. This implies that the administration of AOM and DSS resulted in the upregulation of these markers within colon mucosal cells, thereby contributing to pro-carcinogenic and pro-inflammatory effects. Notably, supplementation with diets enriched with PSO, especially at a concentration of 5% (w/w), mitigated the upregulation of these biomarkers. Despite the relatively low conversion rates of ALA conversion in humans [77, 78], various studies have highlighted the beneficial health effects associated with ALA-rich oils. ALA has demonstrated anti-proliferative and anti-inflammatory effects, culminating in cell cycle arrest, the downregulation of proliferative and inflammatory markers, and an overall anti-carcinogenic impact [24, 31, 32, 74, 76]. The diverse and intricate roles played by ALA-rich PSO indicate its potential in preventing CRC, positioning it as a valuable component of dietary intervention strategies.

Beyond its high ALA content, PSO is also a good source of tocopherols, as demonstrated in our study and supported by previous findings. Tocopherols are lipophilic antioxidants that protect cell membranes and lipid molecules from oxidative damage by donating hydrogen to free radicals, thus halting chain reactions. Their potent antioxidant activity also helps suppress inflammation caused by oxidative stress [79, 80]. In addition to tocopherols, our study shown that PSO contains trace amounts of polyphenols and flavonoids, which further enhance its antioxidant capacity. These phenolic compounds, commonly found in plant-derived oils [81], act by mechanisms such as single electron transfer and hydrogen atom transfer, effectively neutralising free radicals and mitigating oxidative stress [82]. They also possess anti-inflammatory and anti-cancer properties due to their strong antioxidant potential [83]. PSO is also notable for its phytosterol content, particularly β-sitosterol [26], which supports cellular membrane integrity and has been shown to exert anti-cancer effects in rodent models of chemically-induced colon cancer [84, 85]. The combined action of these minor phytoconstituents likely contributes to the suppression of colorectal cancer in our *in vivo* model. Therefore, the anti-cancer effects observed in PSO are likely the result of synergistic interactions between ALA and these other compounds.

Our investigation revealed a notable reduction in the diversity of intestinal microbial communities, particularly within the PC and FC groups, following the induction of CRC in mice through the administration of AOM and DSS. This reduction signified a manifestation of gut dysbiosis associated with the pathological condition. Intriguingly, mice subjected to identical colorectal procarcinogens in the LC, MC, and HC groups, while concurrently fed with experimental diets formulated with 5%, 10%, and 15% perilla oil, respectively, exhibited no alterations in gut microbiome diversity. This observation suggests that the inclusion of PSO in the dietary regimen may mitigate dysbiosis. Numerous investigations have established a correlation between CRC and gut dysbiosis, a relationship corroborated by our findings, independent of the specific AOM/DSS induction protocol employed [9, 10]. Furthermore, prior studies have demonstrated the efficacy of PSO supplementation in ameliorating gut dysbiosis across various disease models [86, 87]. This lends support to the proposition that PSO inclusion in diets could potentially restore microbial diversity in disease-associated conditions. Intriguingly, our study unveiled that a diet comprising 10% (w/w) fish oil failed to alleviate gut microbiome dysbiosis in AOM/DSS-induced CRC mice. This contradicts some previous findings [88, 89], which indicated that n-3 PUFAs in the form of either EPA, DHA, or High-DHA tuna oil, ameliorate gut dysbiosis in mouse models induced with ceftriaxone sodium or high-fat diets.

Our study unveiled a substantial decrease in the abundance of *Bifidobacterium*, a bacterial genus crucially linked to the maintenance of gut barrier integrity, within mice belonging to the PC group mice induced with AOM and DSS, as compared to the NC group. Notably, mice in the LC group, subjected to a 5% (w/w) perilla oil-formulated diet, exhibited a conspicuous increase in *Bifidobacterium* abundance in contrast to the PC group. This implies that the induction of colitis-associated CRC via AOM and DSS exerts a negative impact on *Bifidobacterium* colonization in the intestine milieu, which can be ameliorated by the inclusion of a 5% (w/w) PSO-containing diet. Nevertheless, a higher fat content in the diet manifested a decrease in *Bifidobacterium* abundance, irrespective of the fat type employed [90, 91]. *Faecalibacterium*, recognised for its efficient production of butyrate [92], plays a crucial role in maintaining gut health. Our findings demonstrated a reduced abundance of *Faecalibacterium* in the gut of mice induced with AOM and DSS, aligning with previous studies indicating a substantial reduction in *Faecalibacterium* abundance in CRC-afflicted subjects compared to their healthy individuals [93, 94]. However, diets incorporating 5% (w/w) or 10% (w/w) PSO proved efficacious in sustaining *Faecalibacterium* abundance in CRC-induced mice. Conversely, *Akkermansia* abundance was predominantly overrepresented in the HC and FC group mice, potentially attributed to the higher n-3 PUFAs in their diets [95, 96], although an excess of *Akkermansia* may induce mucus layer degradation and foster inflammation [97–99]. Moreover, an abundance of the *Christensenellaceae* family, intricately associated with weight regulation [100], exhibited a significant increase solely in FC group mice, aligning with their comparatively lower overall weight gain throughout the study.

Our study did not observe any tumour-inhibitory effect of fish oil in our CRC model. This suggests that a diet exclusively composed of fish oil may exert an unfavourable influence on disease progression, in contrast to combinations of fish oil with other dietary fat sources, irrespective of concentration [45, 46, 101, 102]. The contrasting effects between fish oil and PSO diets in our study may be due to the distinct modes of action of different types of n-3 PUFAs. Both EPA and DHA are considered active forms of n-3 PUFAs, exerting their anti-inflammatory effects primarily through enzymatic oxidation, yielding anti-inflammatory eicosanoids. While the conversion of ALA to EPA and DHA in mammals and humans is relatively low [103], several studies have shown that ALA can exert anti-inflammatory effects independently. ALA acts through pathways such as NF-κB and iNOS [104, 105], reduces the expression of vascular and cellular adhesion molecules [106], and suppresses pro-inflammatory cytokines like TNF-α and IL-1β [107]. This suggests that ALA may not require conversion to EPA or DHA to exert its anti-inflammatory effects, as evidenced by low levels of long-chain n-3 PUFAs detected in the plasma and colon tissues of mice fed ALA-rich oils [29, 31, 108]. Additionally, in vitro studies report that ALA has anti-cancer properties, including upregulation of tumour suppressor proteins such as p53, p21, and p27, and modulation of matrix metalloproteinases [23, 109]. ALA has also been shown to inhibit COX-2 and cyclin D1 expression, which may contribute to its anti-cancer effects [18, 110]. Collectively, these findings suggest that ALA itself can act as an anti-inflammatory and anti-cancer agent independently of its conversion to long-chain n-3 PUFAs.

On the other hand, several studies have reported that fish oil, despite its known benefits, may have deleterious effects on colitis- and infection-driven CRC. For instance, Woodworth et al. demonstrated that SMAD3^−/−^ mice fed a DHA-enriched fish oil diet had significantly higher colon histopathology scores and increased colon dysplasia compared to those fed corn oil or safflower oil diets in a *Helicobacter hepaticus*-mediated CRC model [111]. Similarly, in an IL-10^−/−^ colitis model, mice fed a 7% (w/w) fish oil diet exhibited increased colitis scores, aberrant crypt counts, colon dysplasia, and elevated COX-2 expression in colon tissue [112]. In another study using a DSS-induced colitis model, C57BL/6 mice fed an 8% (w/w) fish oil diet showed increased TNF-α mRNA expression and inflammatory cell infiltration in the colonic mucosa [113]. The mechanisms underlying this exacerbation of inflammation and CRC by fish oil may involve alterations in CD8^+^ T-cell populations and FOXP3 expression [111], increased macrophage secretion of pro-inflammatory TNF-α, reduced anti-inflammatory IL-10 secretion [114], and reduced adiponectin expression in colon tissues [113]. Another potential explanation for the detrimental effects of fish oil in our study could be the increased oxidative stress associated with its long-chain PUFAs. EPA and DHA, being highly unsaturated, are particularly susceptible to oxidation by free radicals generated during inflammation, which leads to lipid peroxidation and subsequent damage to vital cellular biomolecules [115]. Studies have shown that dietary fish oil can enhance cellular oxidative stress and oxidative damage, as indicated by elevated TBARS levels and reduced Nrf2 expression and antioxidant enzyme activities in various in vivo models [116–118]. Collectively, these findings suggest that the protective effects of fish oil in colitis-associated CRC remain inconclusive, as studies, including ours, have produced mixed results. Further comprehensive exploration is needed to elucidate the exact role and mechanisms of fish oil in inflammation-driven CRC.

High-fat diets, especially those with 20% PSO, exhibited no tumour inhibitory effects in our model. This observation is consistent with prior studies indicating a potential adverse impact of high-fat diets on CRC progression, regardless of the type of fat used [119, 120]. Specifically, we observed that mice fed a 5% (w/w) PSO diet exhibited the most effective tumour inhibition, while those fed higher concentrations of PSO (10% or 20%) demonstrated less tumour inhibition. This dose-dependent effect suggests that, although PSO contains beneficial n-3 fatty acids, its protective effects may be attenuated at higher fat levels. The attenuation of PSO’s efficacy in higher fat diets could be due to several factors. First, high-fat diets are known to promote chronic inflammation, which is a key driver of CRC [121]. Regardless of the fat type, excess dietary fat can stimulate pro-inflammatory pathways and increase the production of pro-inflammatory cytokines, which in turn exacerbates tumorigenesis [122]. Additionally, high-fat diets may alter the gut microbiota in ways that promote cancer development by increasing the abundance of pro-inflammatory bacterial species [123]. These mechanisms could explain why the tumour-suppressive effects of PSO were less pronounced at higher concentrations in our study. Moreover, the negative impact observed with high-fat PSO diets parallels the effects seen with fish oil diets in our study, where mice fed 10% (w/w) fish oil showed increased tumour progression. This further reinforces the idea that, while specific fats like n-3 PUFAs from PSO or fish oil may have protective properties, the overall fat content of the diet plays a critical role in modulating cancer outcomes [124]. Thus, our results suggest that while PSO may have beneficial anti-cancer properties, the total fat content of the diet must be carefully considered, as excessive fat intake may negate the positive effects of n-3 fatty acids in preventing CRC. Further studies are needed to better understand the precise mechanisms by which dietary fat levels influence the protective effects of PSO.

We observed that PSO was able to maintain microbial diversity and increase the abundance of beneficial gut bacteria such as *Bifidobacterium* and *Faecalibacterium*, which are known for their roles in maintaining gut health and integrity. In contrast, a diet rich in fish oil failed to restore microbial balance, aligning with findings from other studies suggesting that n-3 PUFAs from fish oil may not always have a beneficial effect on gut health in inflammatory conditions. Furthermore, the changes in specific microbial populations, such as the increase in *Akkermansia* in the high-fat groups (including both PSO and fish oil), accentuate the complexity of the relationship between diet, gut microbiota, and CRC development. While *Akkermansia* has been associated with gut health, its overabundance may degrade the mucus layer and exacerbate inflammation, as seen in high-fat diets. These findings suggest that while dietary fats like PSO may support gut health, excessive fat intake, regardless of the source, could disrupt microbial balance and contribute to CRC progression.

Our study provides insights into the mechanistic pathways through which ALA-rich PSO exerts its anti-cancer effects in the AOM/DSS-induced colitis-associated CRC model. Notably, PSO targets the Wnt signalling pathway, downregulating β-catenin and its downstream target cyclin D1, which are key drivers of tumorigenesis. This is supported by the decreased expression of β-catenin, cyclin D1, and PCNA observed in colon tissue sections and confirmed by western blot analysis. Additionally, PSO appears to modulate the ERK signalling pathway, as demonstrated by reduced Ras expression in the colon tissues, suggesting a potential role in controlling cellular proliferation. Inflammation, a major contributor to CRC development, was also markedly suppressed by PSO through the inhibition of NF-κB and COX-2 expression, further corroborating its anti-inflammatory and anti-tumorigenic effects. In parallel, PSO promoted gut homeostasis by mitigating dysbiosis and enhancing the abundance of short-chain fatty acid-producing bacteria, which are known to contribute to gut health and reduce CRC risk. These findings indicate that the anti-cancer activity of PSO is mediated through multiple interrelated pathways, targeting both cancer cell proliferation and inflammation while promoting gut health.

In the present study, we focused on the effects of ALA, which is the major n-3 PUFA in PSO, constituting approximately 61% of its total fatty acid content. ALA has been shown to have potent anti-inflammatory and anticancer effects, and our aim was to assess its role in inflammation-driven CRC. However, we acknowledge that PSO contains other bioactive compounds, including tocopherols, polyphenols, and flavonoids, which may contribute to its overall biological activity. While the protective effects observed in our study may be largely due to ALA, the potential contribution of these other phytochemicals should not be overlooked. It is plausible that the phytochemicals in perilla oil enhanced the overall therapeutic effects, and further studies are warranted to explore the individual contributions of these components.

## Conclusions

This study aimed to evaluate the efficacy of cold press perilla seed oil in attenuating colorectal cancer inhibition, cell proliferation, inflammation responses, and gut microbiota diversity using an AOM/DSS-induced colitis-associated colorectal cancer mouse model. Perilla seed oil, characterized by its abundance in n-3 ALA & tocopherols, demonstrated a notable capability to hinder the development of colonic tumours. This inhibitory effect appears to be mediated through the modulation of crucial proteins associated with cell cycle regulation and inflammation within the colon mucosal cells. Furthermore, perilla seed oil exhibited a mitigating impact on AOM/DSS-induced gut microbiome dysbiosis, surpassing the prophylactic efficacy of fish oil against colitis-associated colorectal cancer. The consumption of perilla oil holds promise for conferring health benefits in both the prevention and treatment of colorectal cancer. Despite the valuable insights provided by this study, further research endeavours are imperative to elucidate the specific influences of perilla seed oil on key proteins intricately involved in colorectal cancer pathogenesis and to explore its potential impact on epigenetic modifications. Furthermore, subsequent investigations should incorporate human studies to assess the efficacy of perilla seed oil in the context of ulcerative colitis, thereby fostering a more comprehensive understanding of its effects on the intestinal microbiome. The results presented herein underscore the potential of perilla seed oil as a therapeutic and preventive agent against colorectal cancer yet underscore the necessity for additional inquiries to refine and expand upon our current understanding of its multifaceted impacts.

## Supporting information

S1 Data (XLSX)

S2 Data (XLSX)

S3 Data (XLSX)

S4 Data (XLSX)

S5 Data (XLSX)

S6 Data (XLSX)

S7 Data (XLSX)

S8 Data (ZIP)

S9 Data (XLSX)

S1 Raw images (PDF)

S2 Raw images (PDF)

S1 File (PDF)
