# Supplementary material for: Cold-pressed extraction of perilla seed oil enriched with alpha-linolenic acid mitigates tumour progression and restores gut microbial homeostasis in the AOM/DSS mice model of colitis-associated colorectal cancer
Source: PLoS One. 2024 Dec 9;19(12):e0315172. doi: 10.1371/journal.pone.0315172 (PMC11627366; doi:10.1371/journal.pone.0315172)
Supplement: S1 Raw images — (PDF) [file pone.0315172.s010.pdf]

β-catenin: analysed using QuPath software

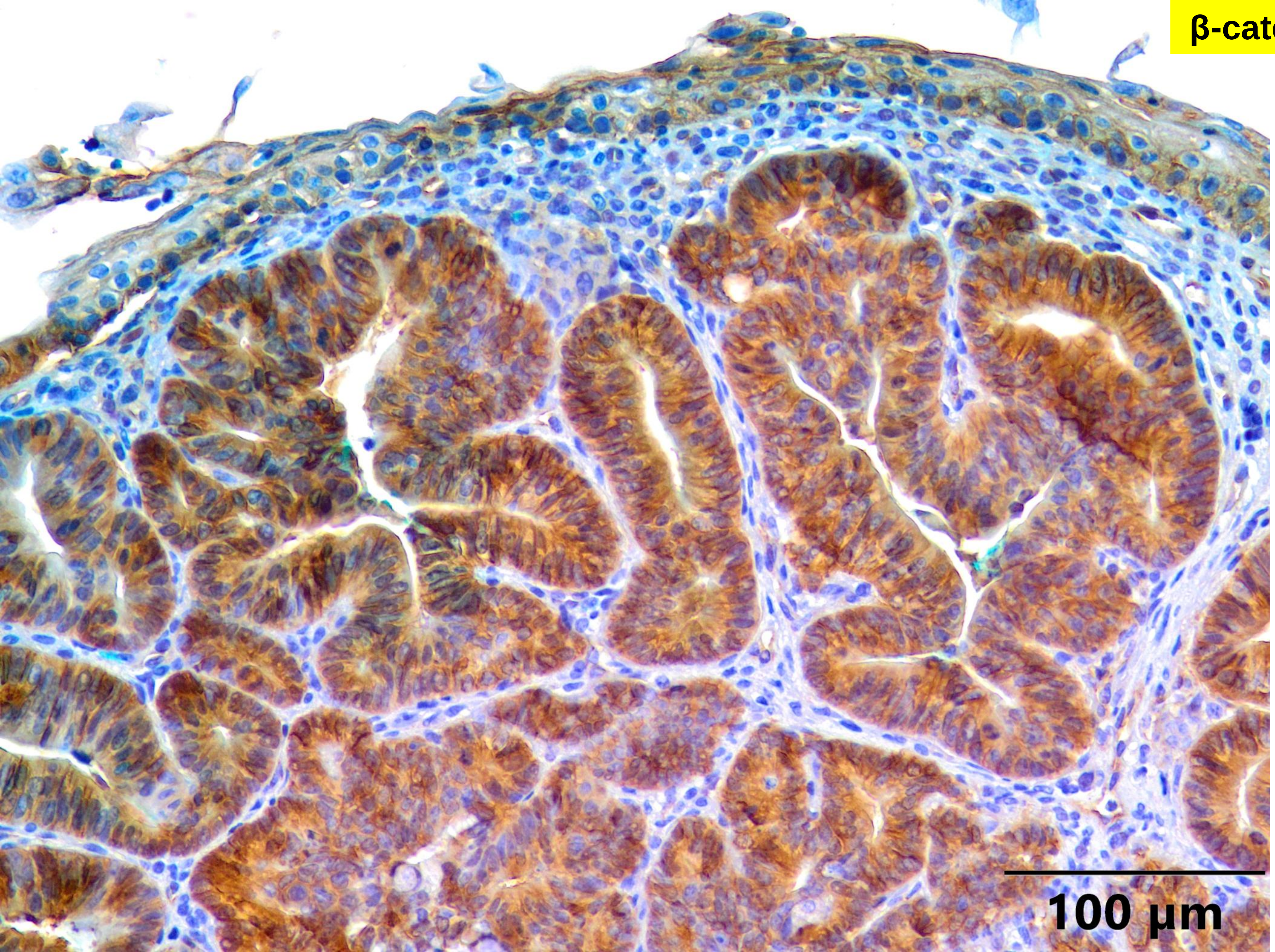

Original micrograph

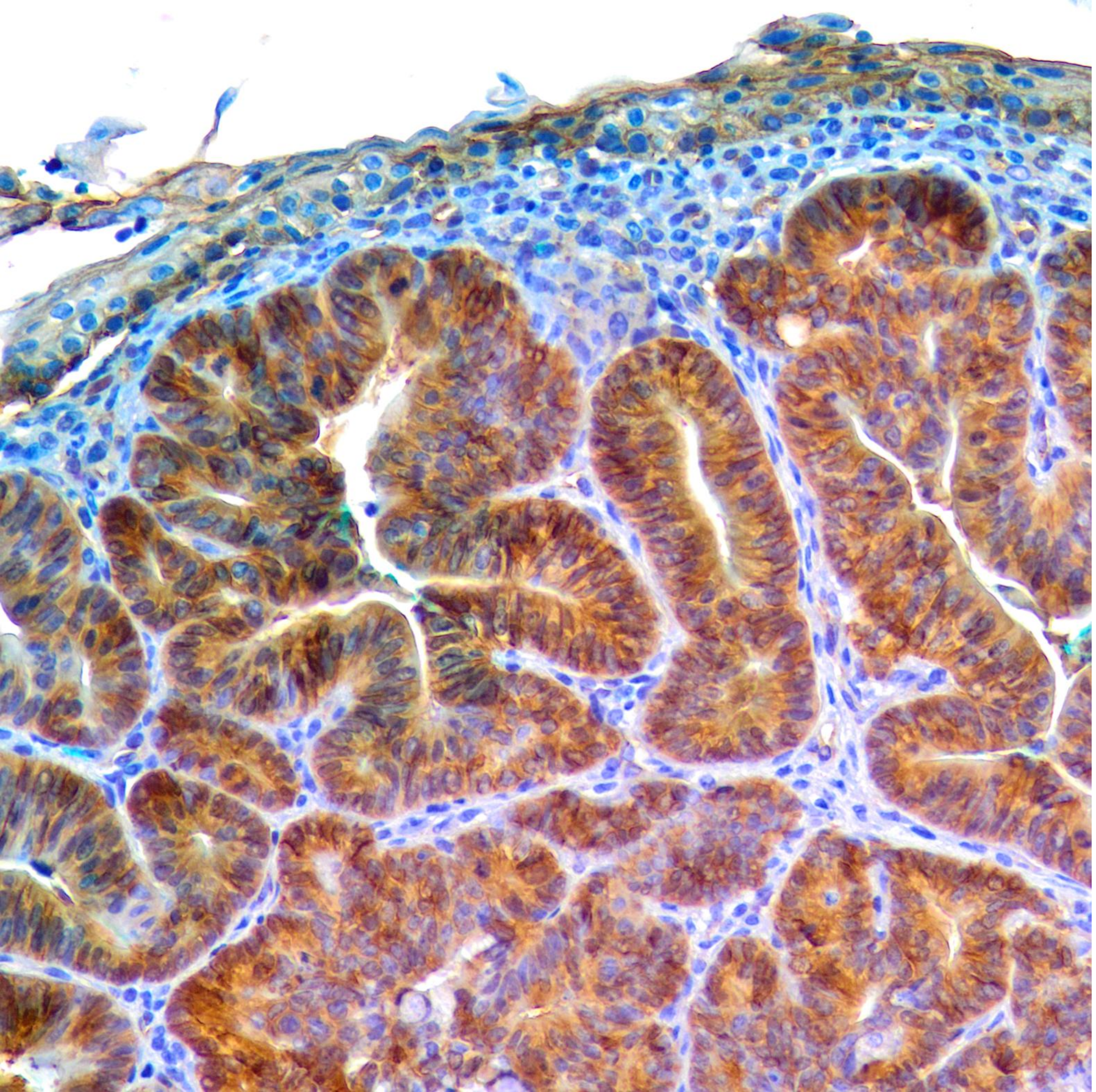

β-catenin: analysed using QuPath software

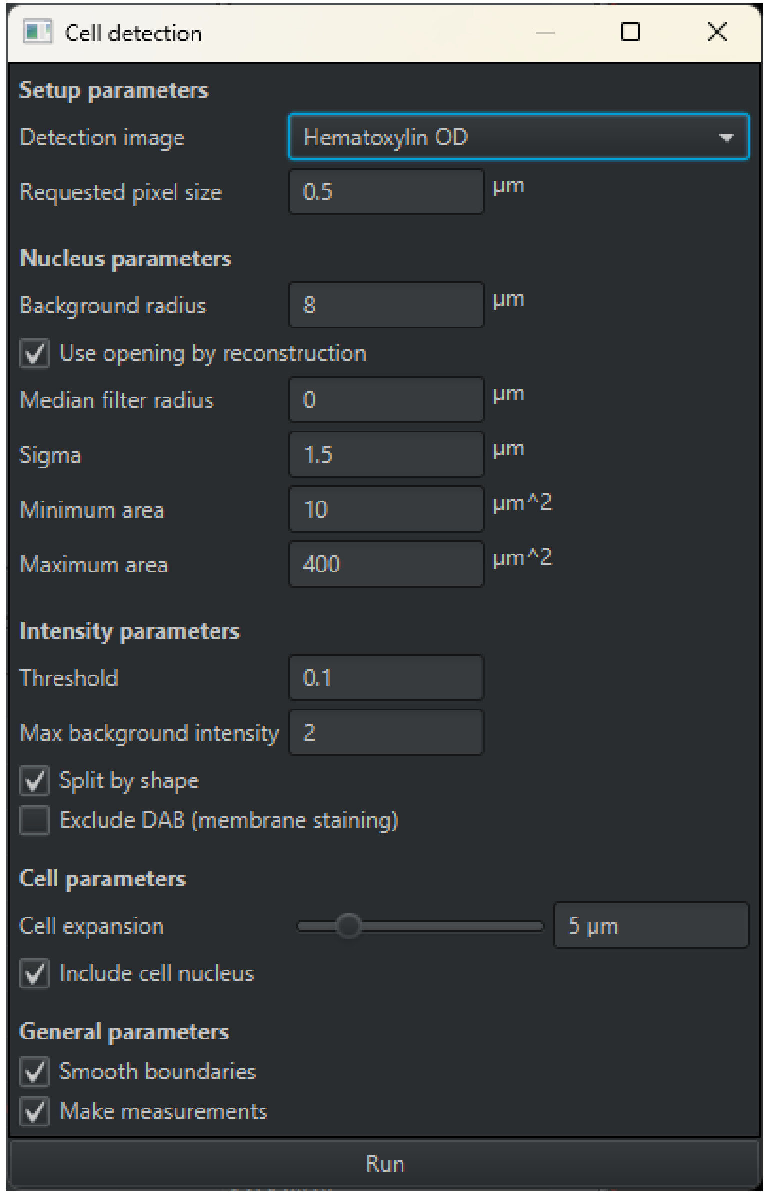

Select the area for IHC analysis and count all cells in the area

β-catenin: analysed using QuPath software

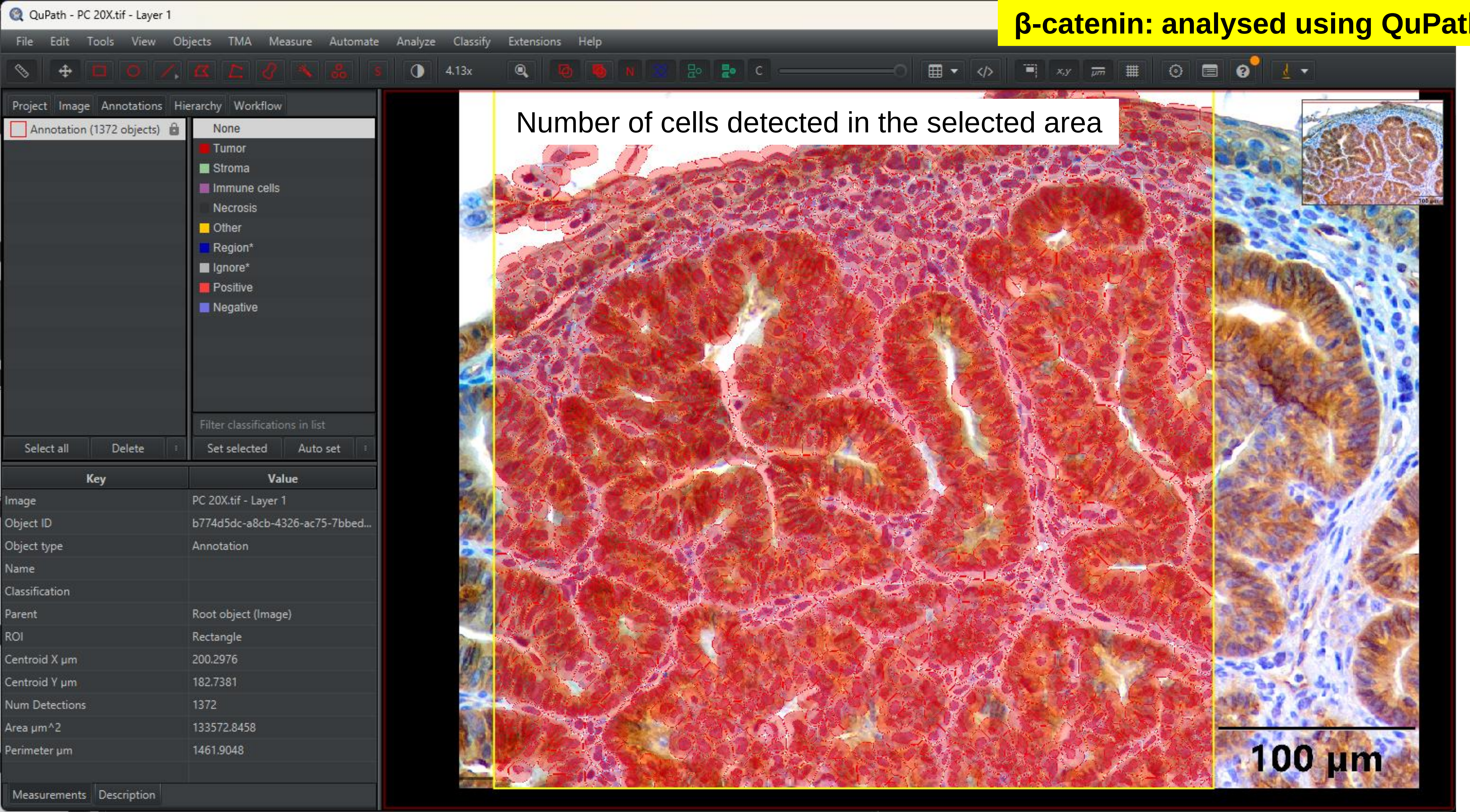

β-catenin: analysed using QuPath software

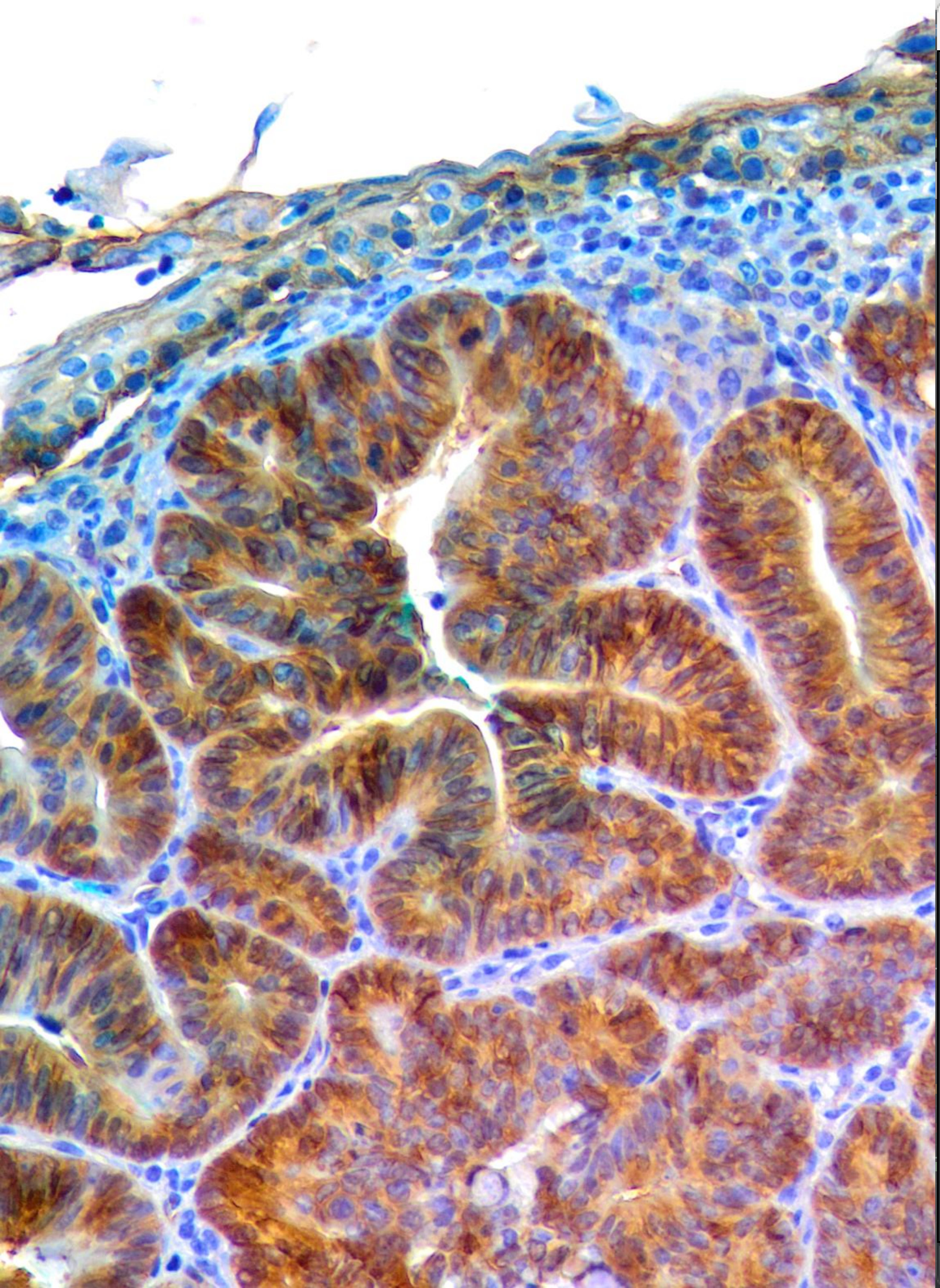

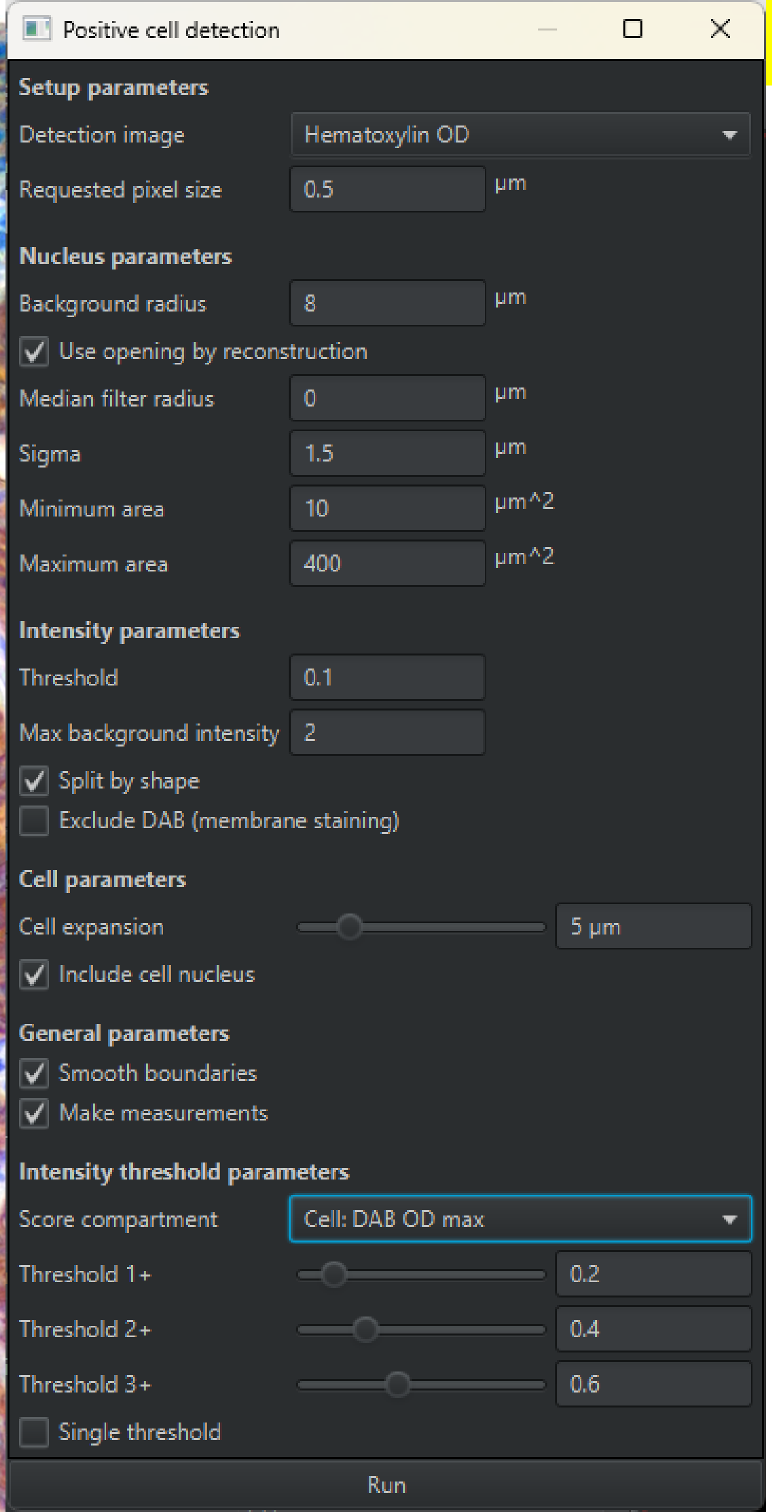

Setup the parameters for analysis of immunopositive cells in the IHC micrograph.

For β-catenin, use cell DAB OD max for the detection.

β-catenin: analysed using QuPath software

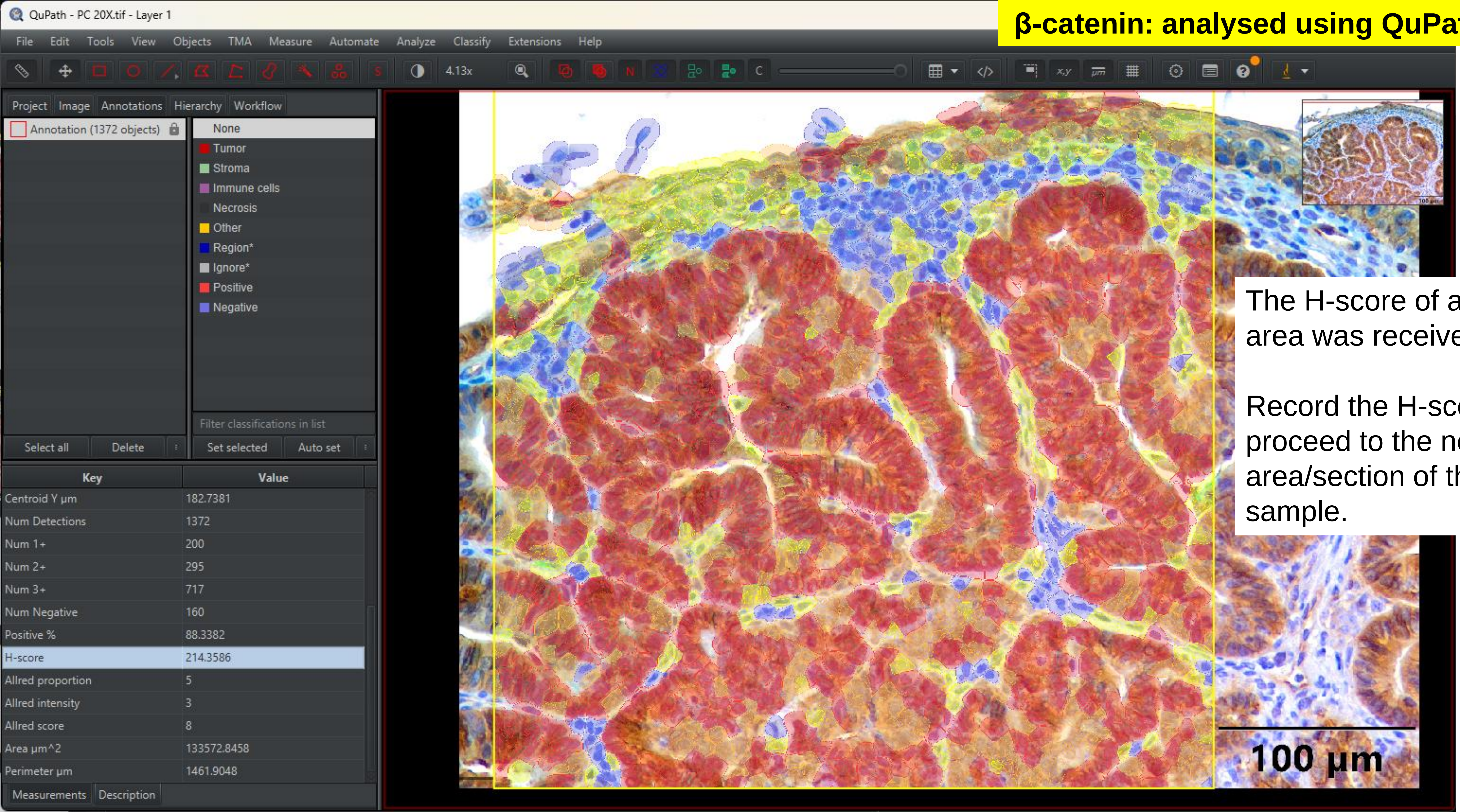

The H-score of a selected area was received.

Record the H-score and proceed to the next area/section of the same sample.

PCNA: analysed using QuPath software

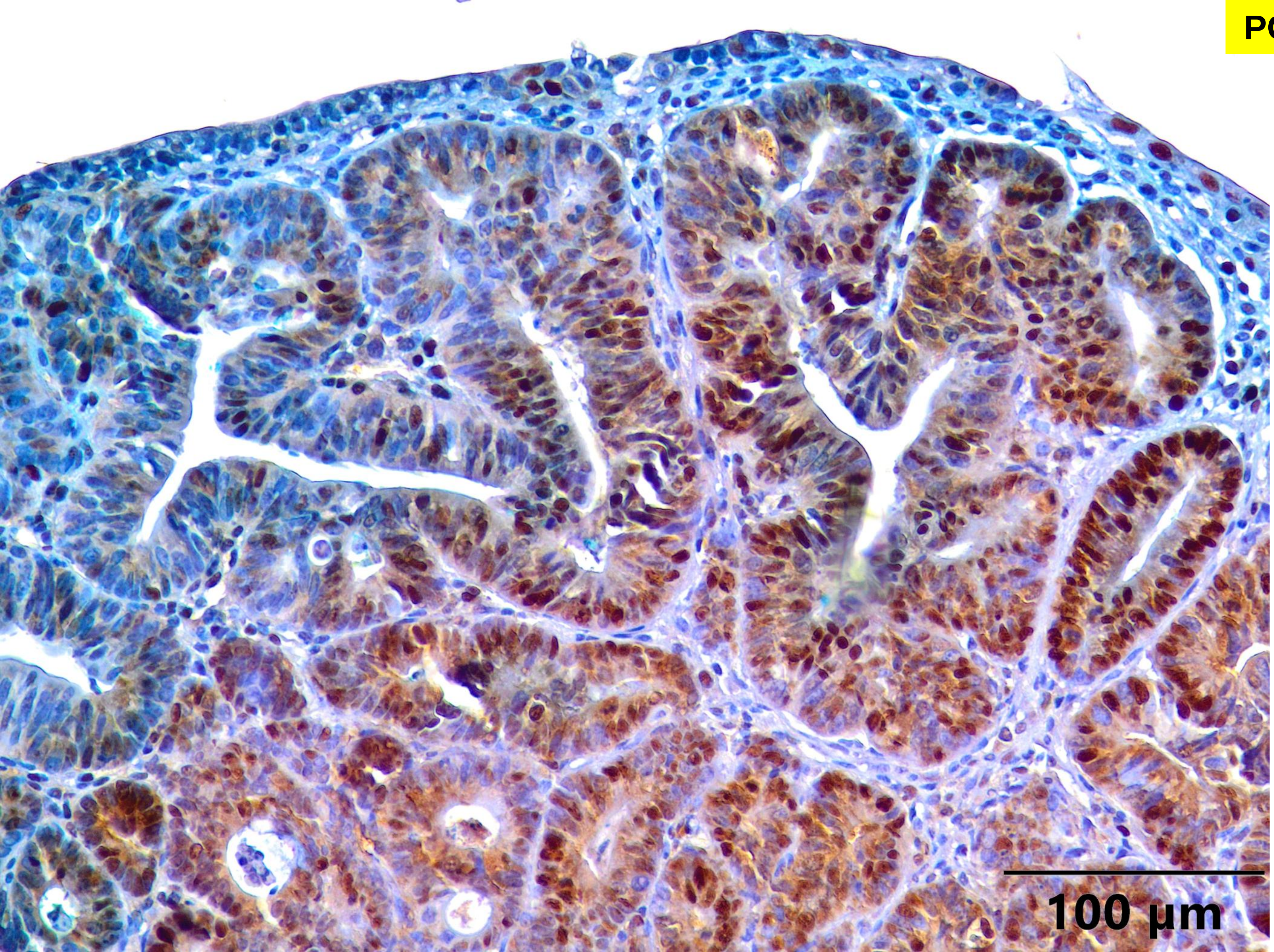

Original micrograph

PCNA: analysed using QuPath software

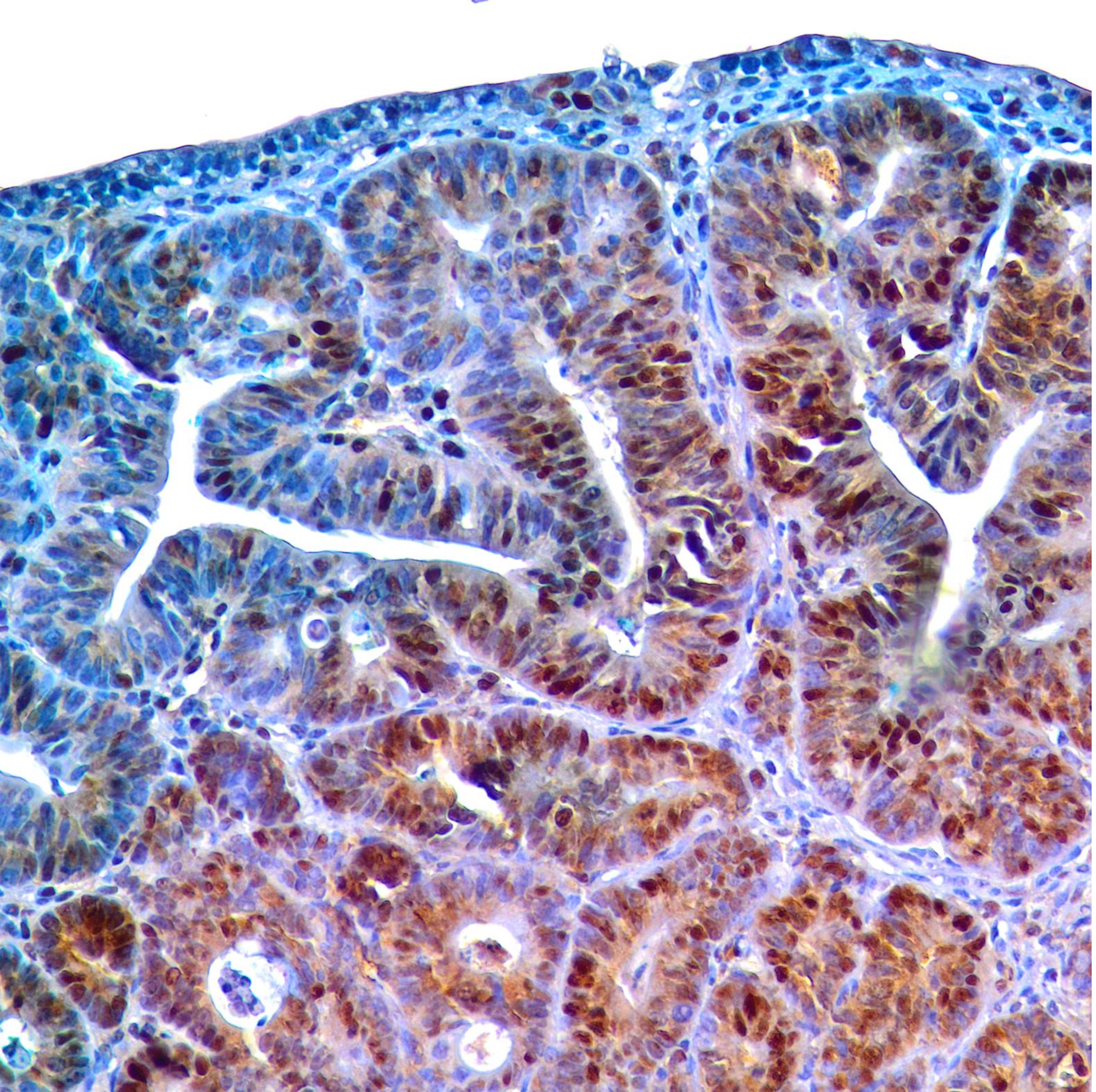

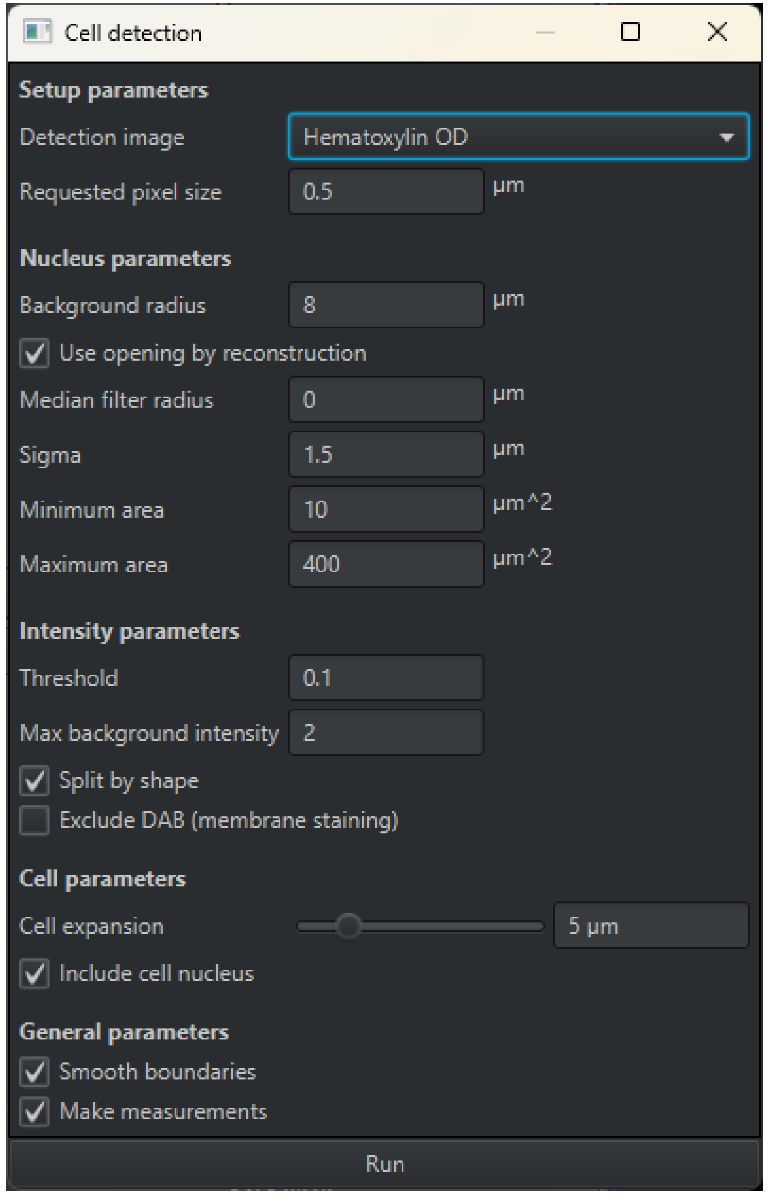

Select the area for IHC analysis and count all cells in the area

PCNA: analysed using QuPath software

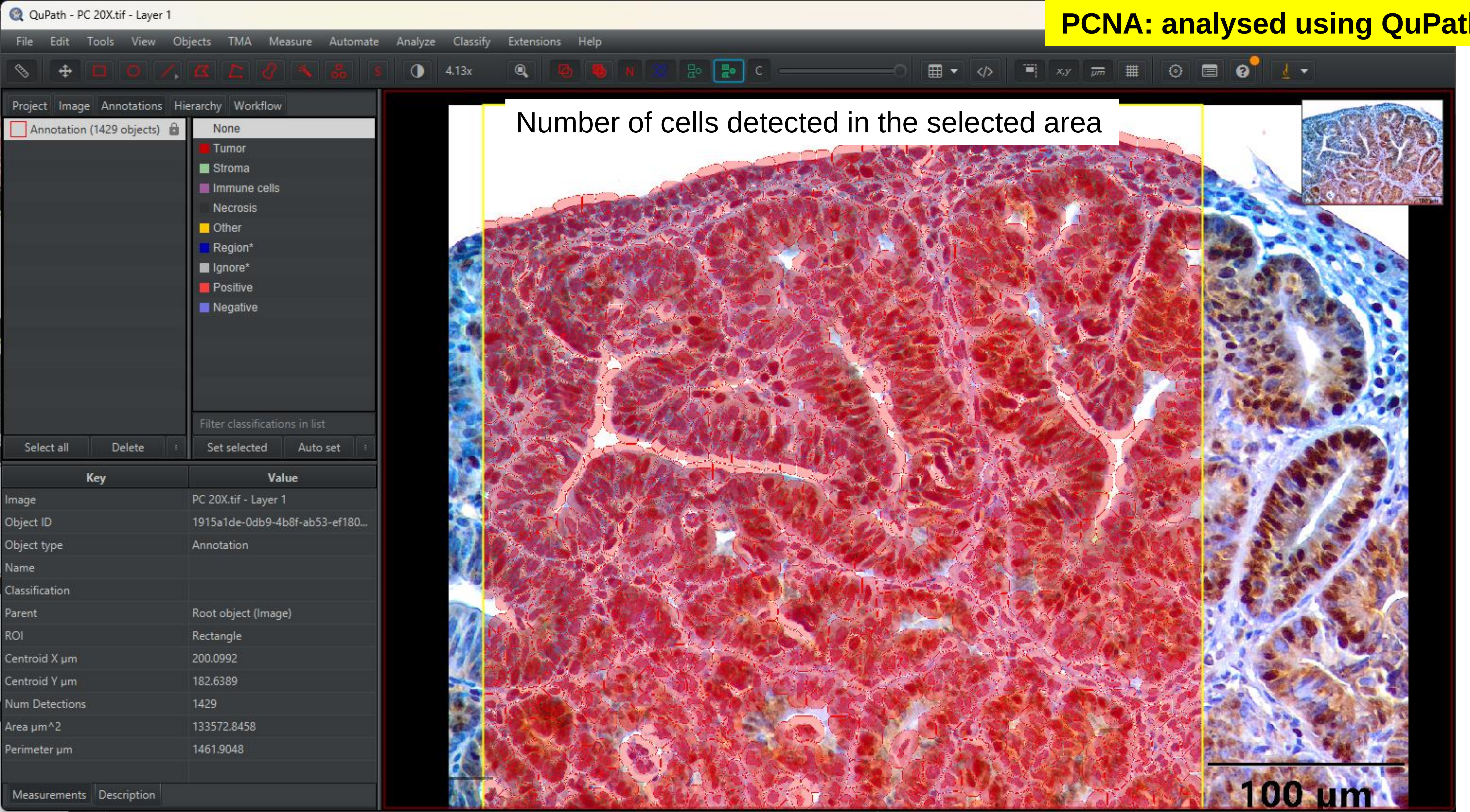

PCNA: analysed using QuPath software

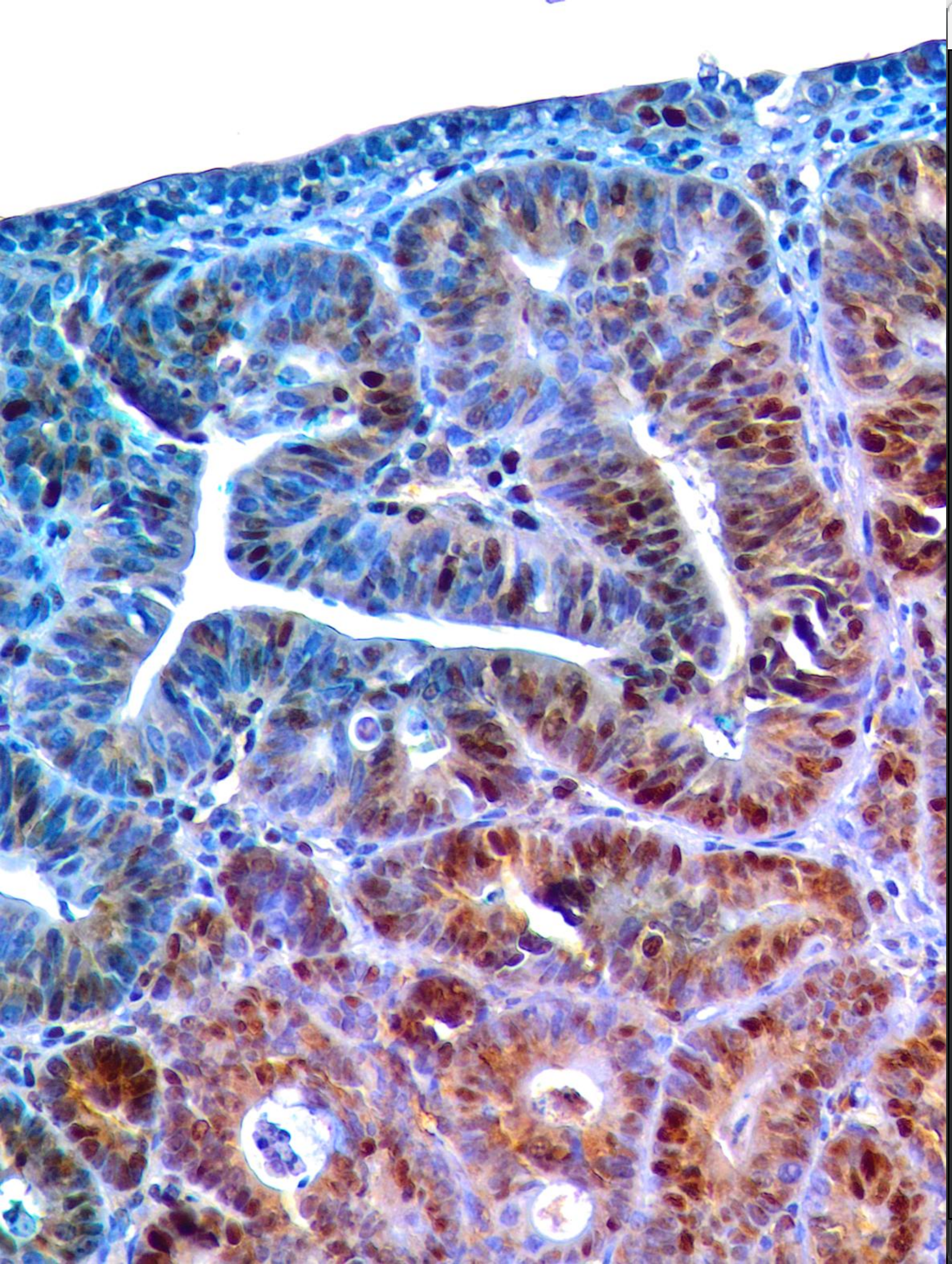

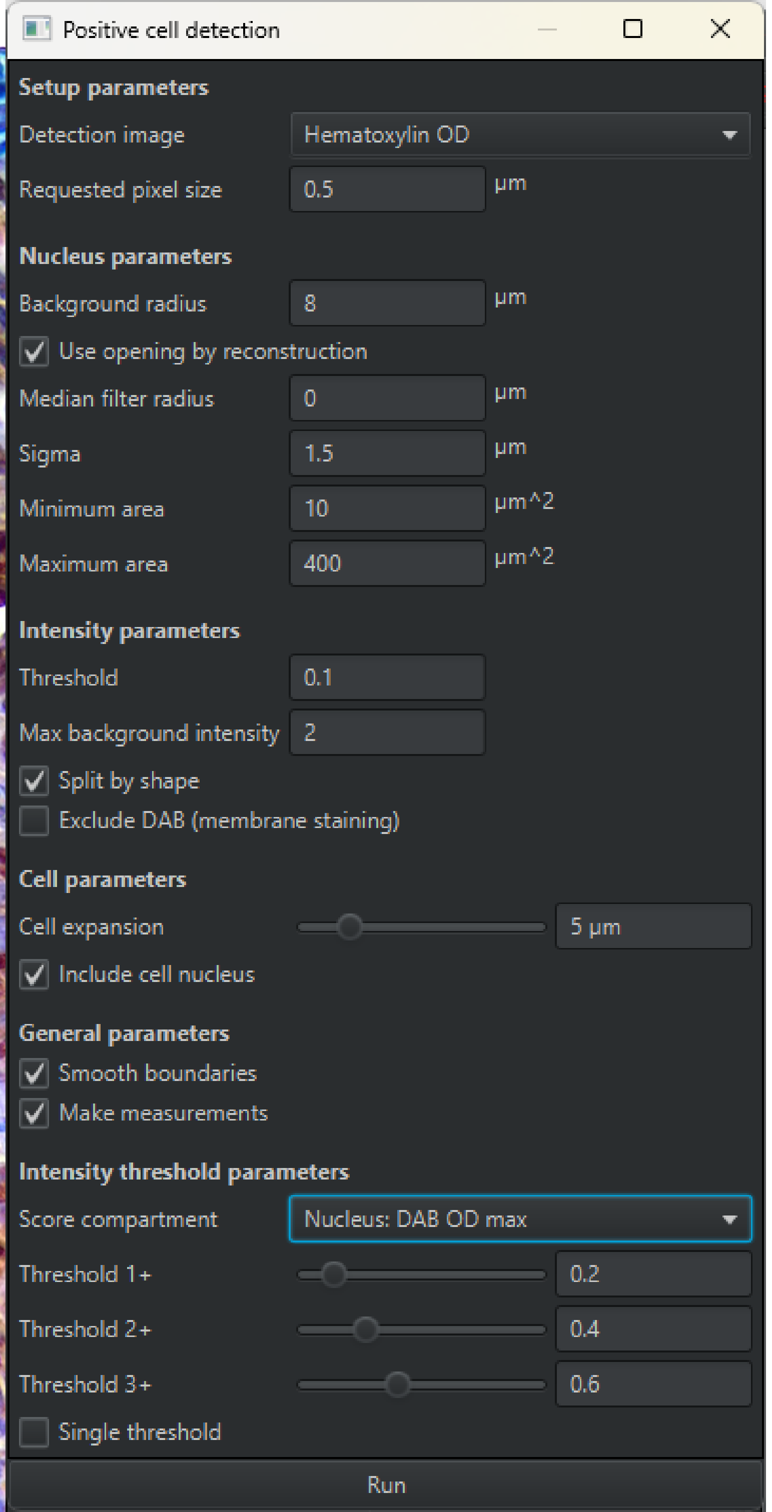

Setup the parameters for analysis of immunopositive cells in the IHC micrograph.

For PCNA and cyclin D1, use nuclear DAB OD max for the detection.

PCNA: analysed using QuPath software

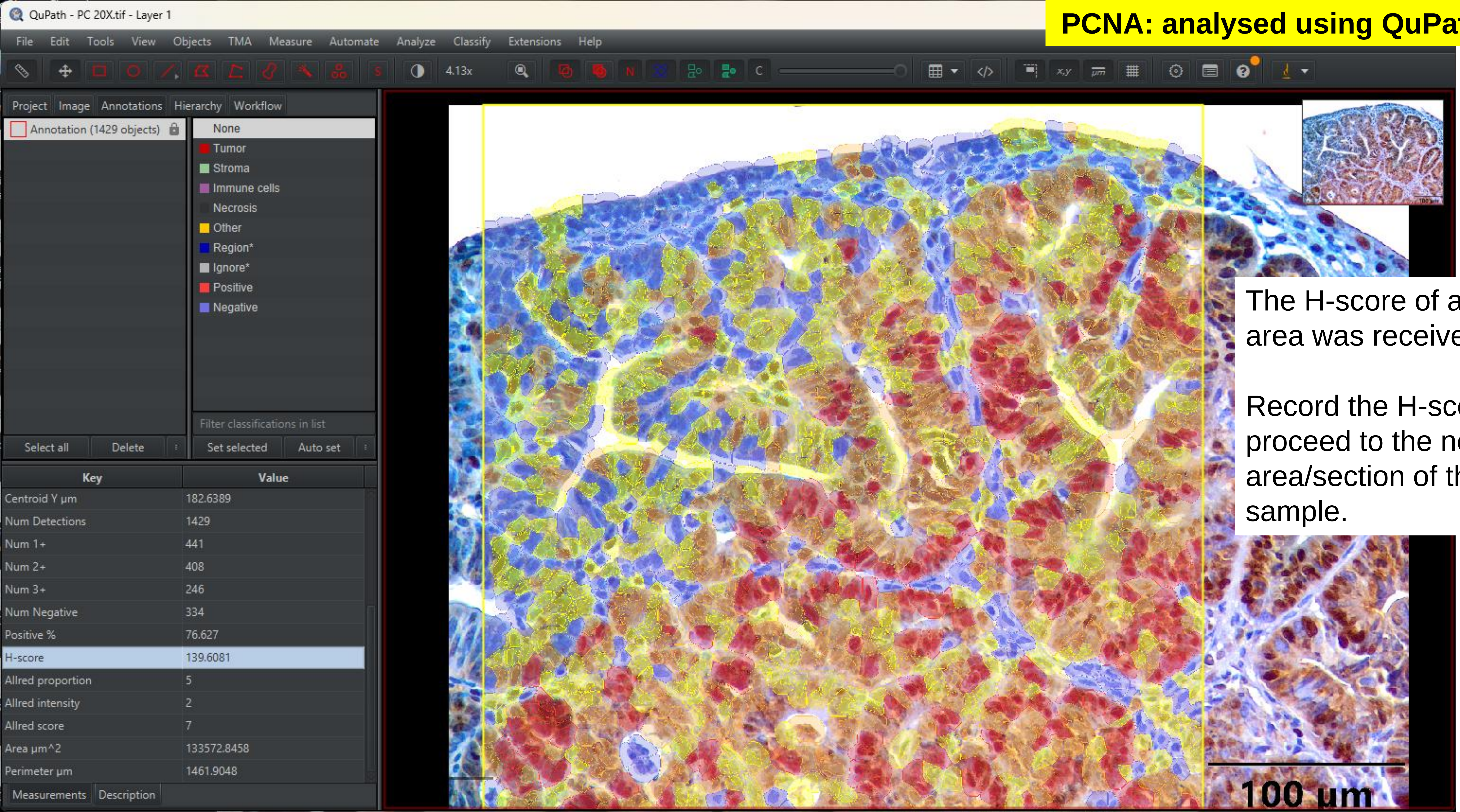

The H-score of a selected area was received.

Record the H-score and proceed to the next area/section of the same sample.

Cyclin D1: analysed using QuPath software

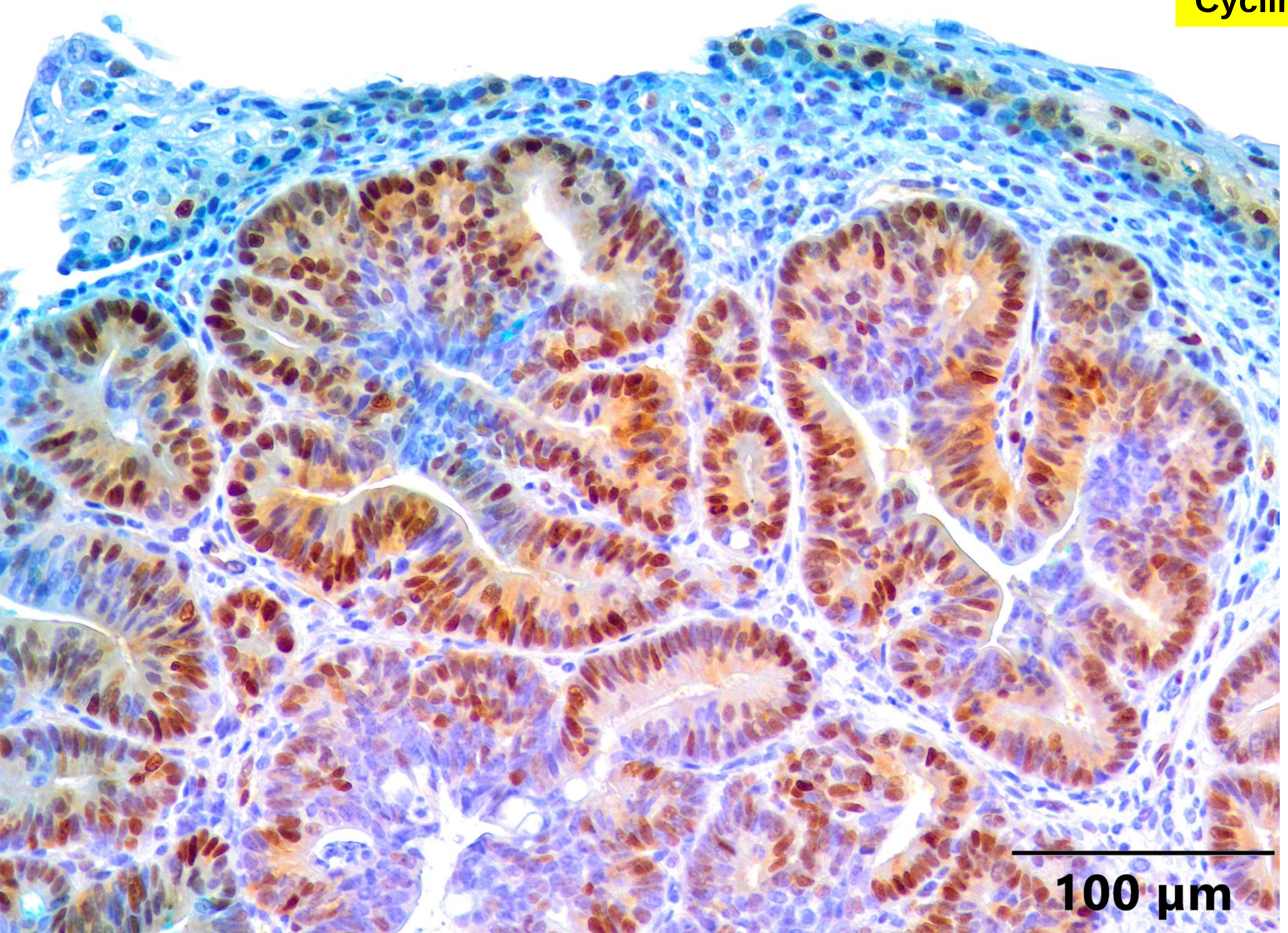

Original micrograph

Cyclin D1: analysed using QuPath software

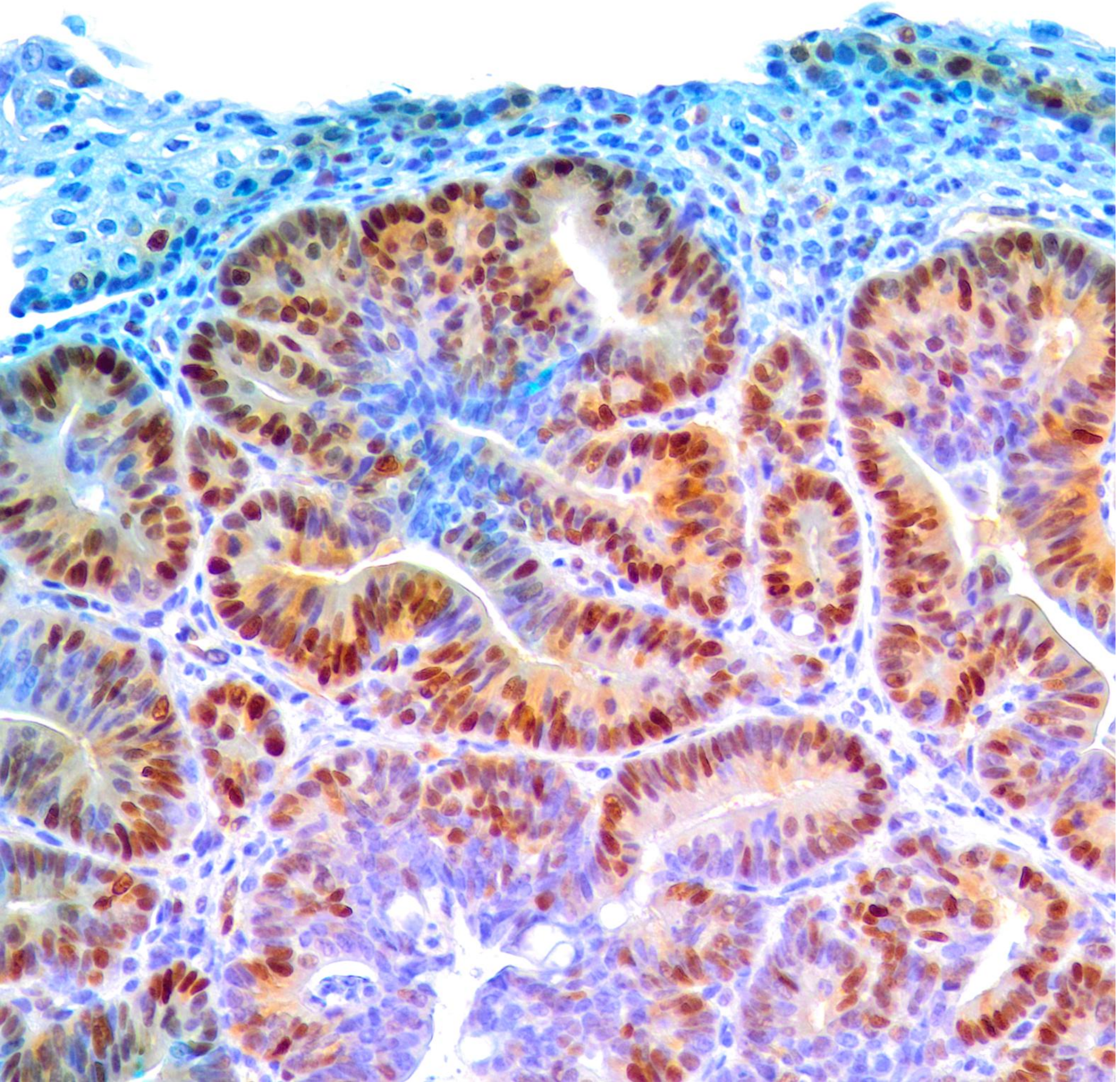

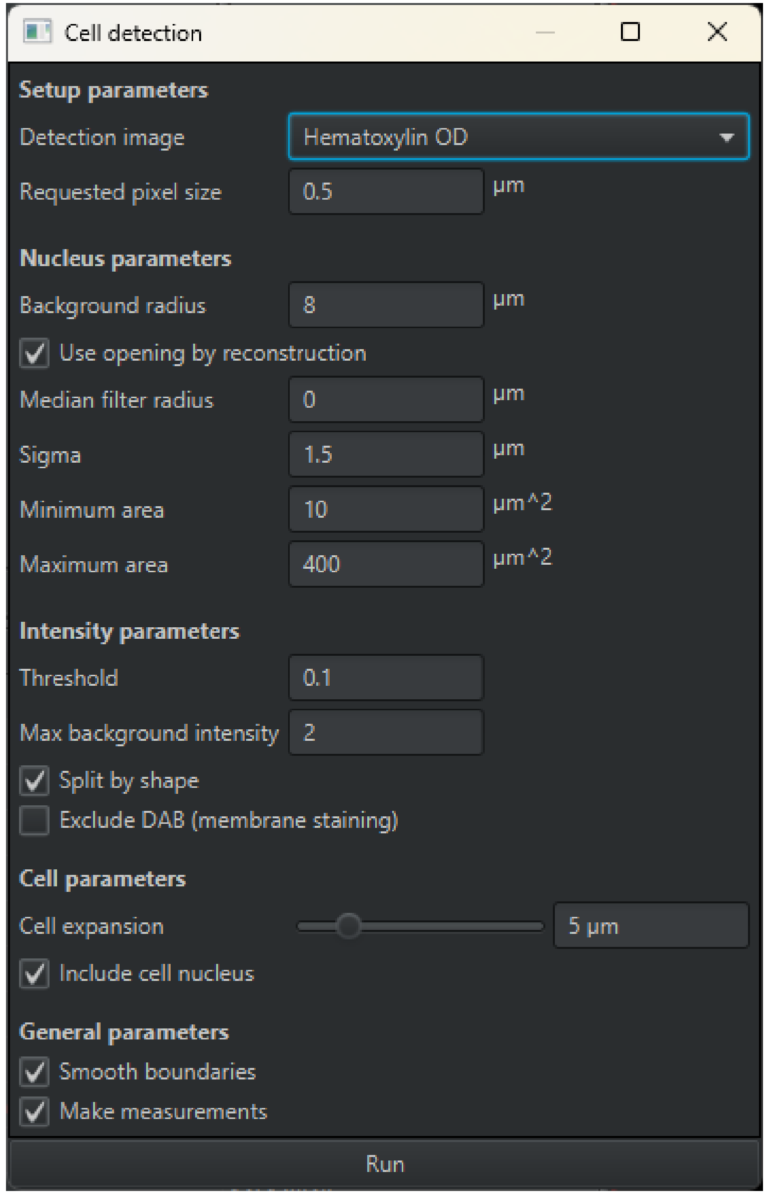

Select the area for IHC analysis and count all cells in the area

Cyclin D1: analysed using QuPath software

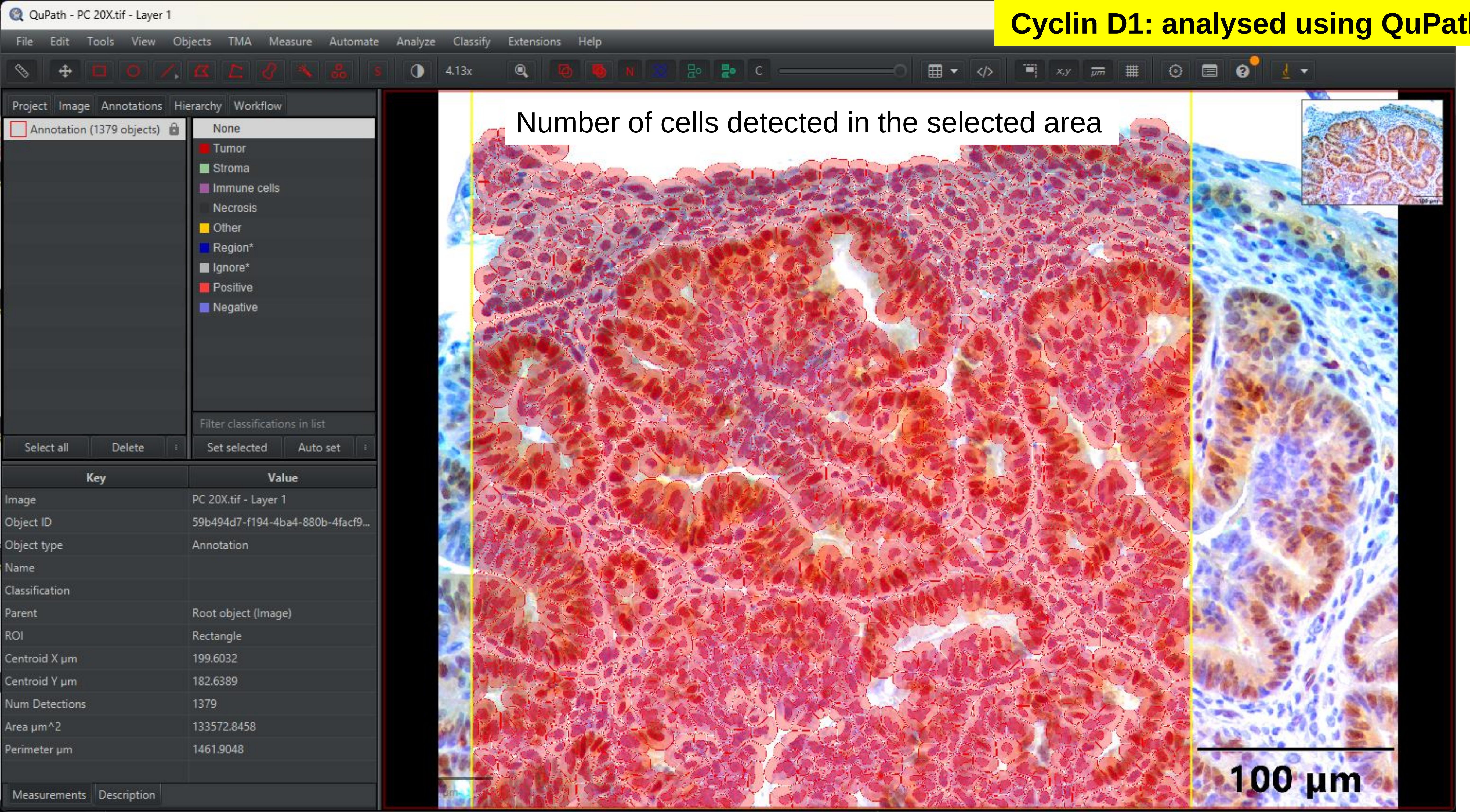

Cyclin D1: analysed using QuPath software

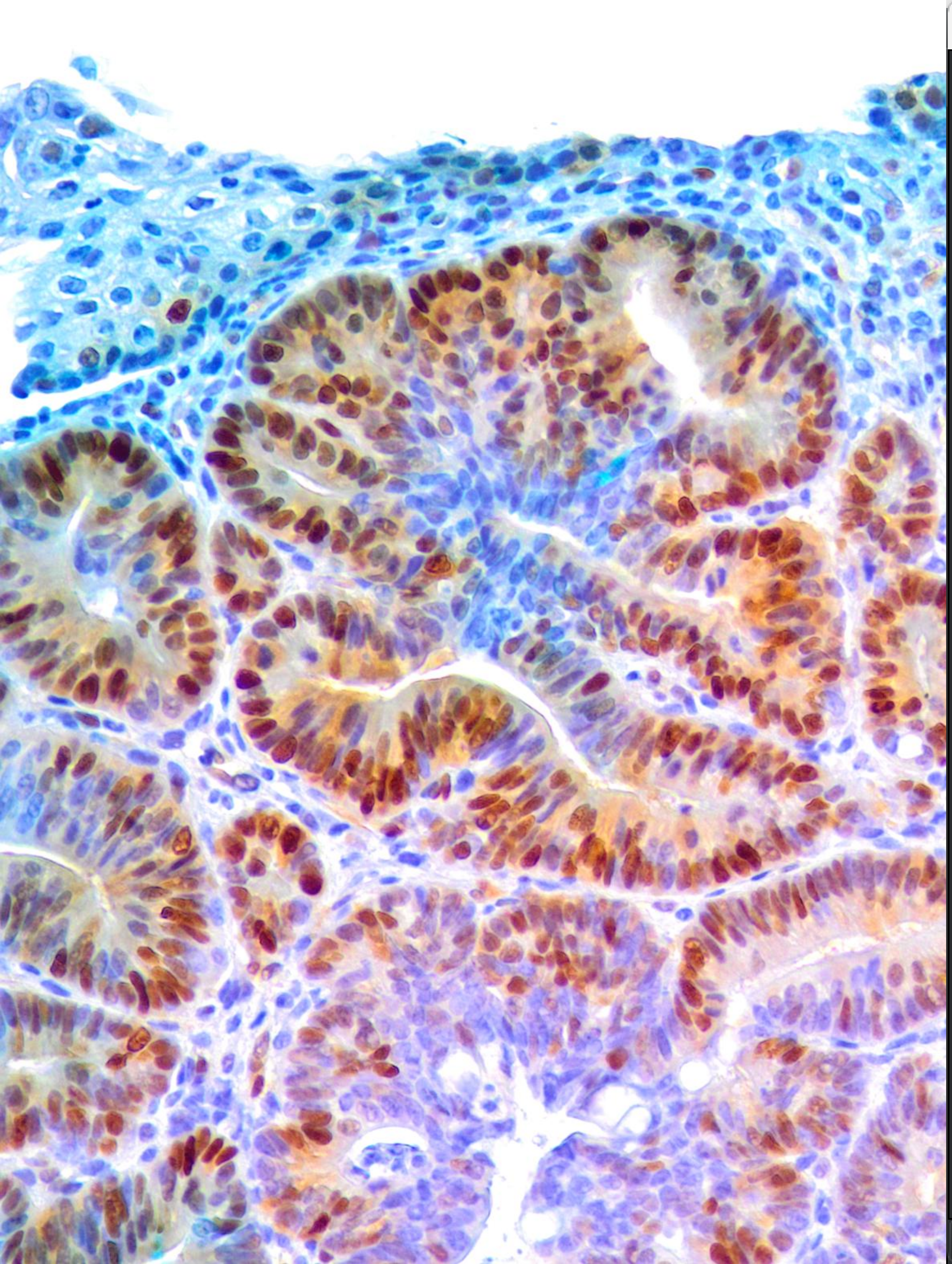

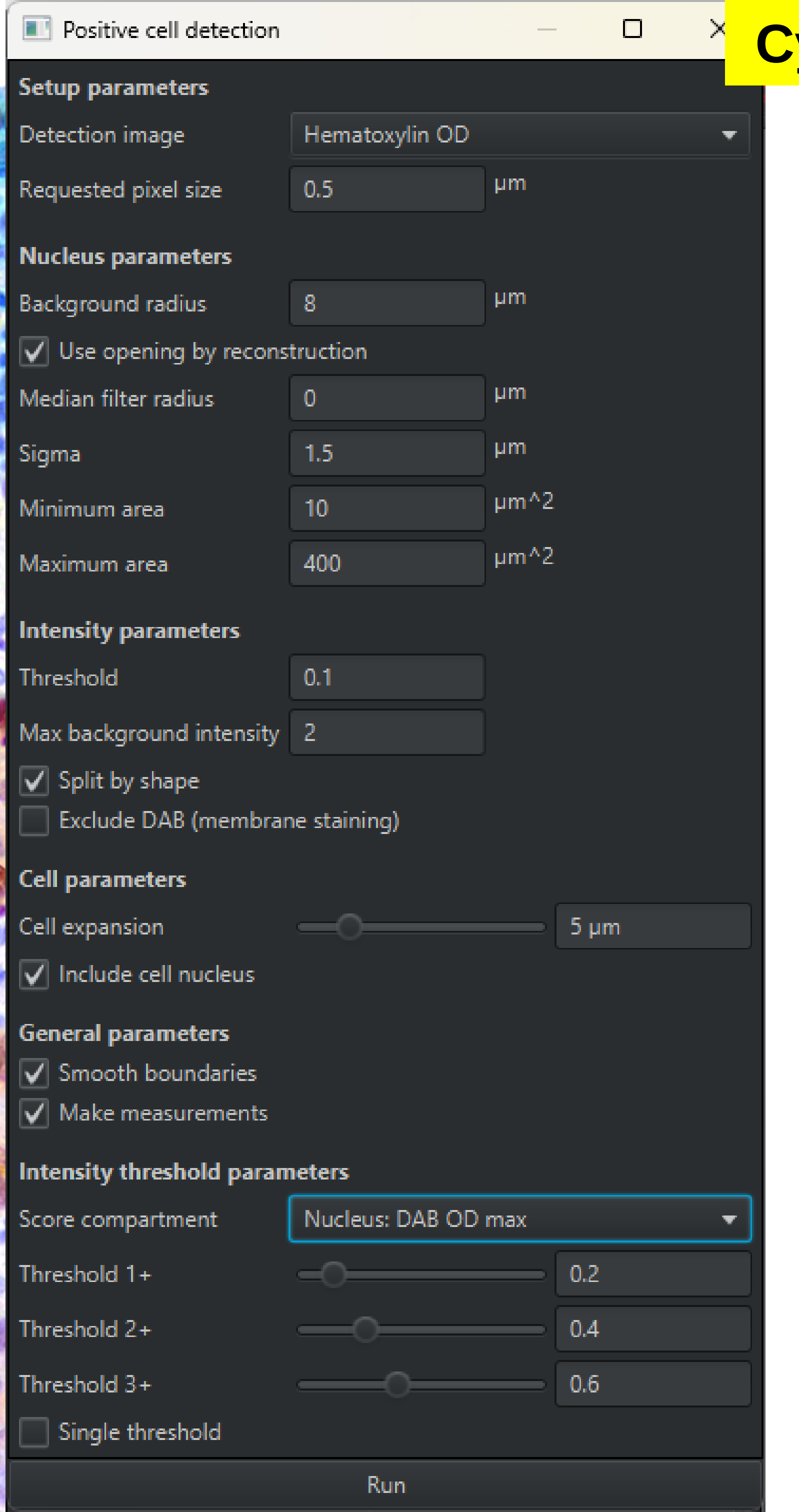

Setup the parameters for analysis of immunopositive cells in the IHC micrograph.

For PCNA and cyclin D1, use nuclear DAB OD max for the detection.

Cyclin D1: analysed using QuPath software

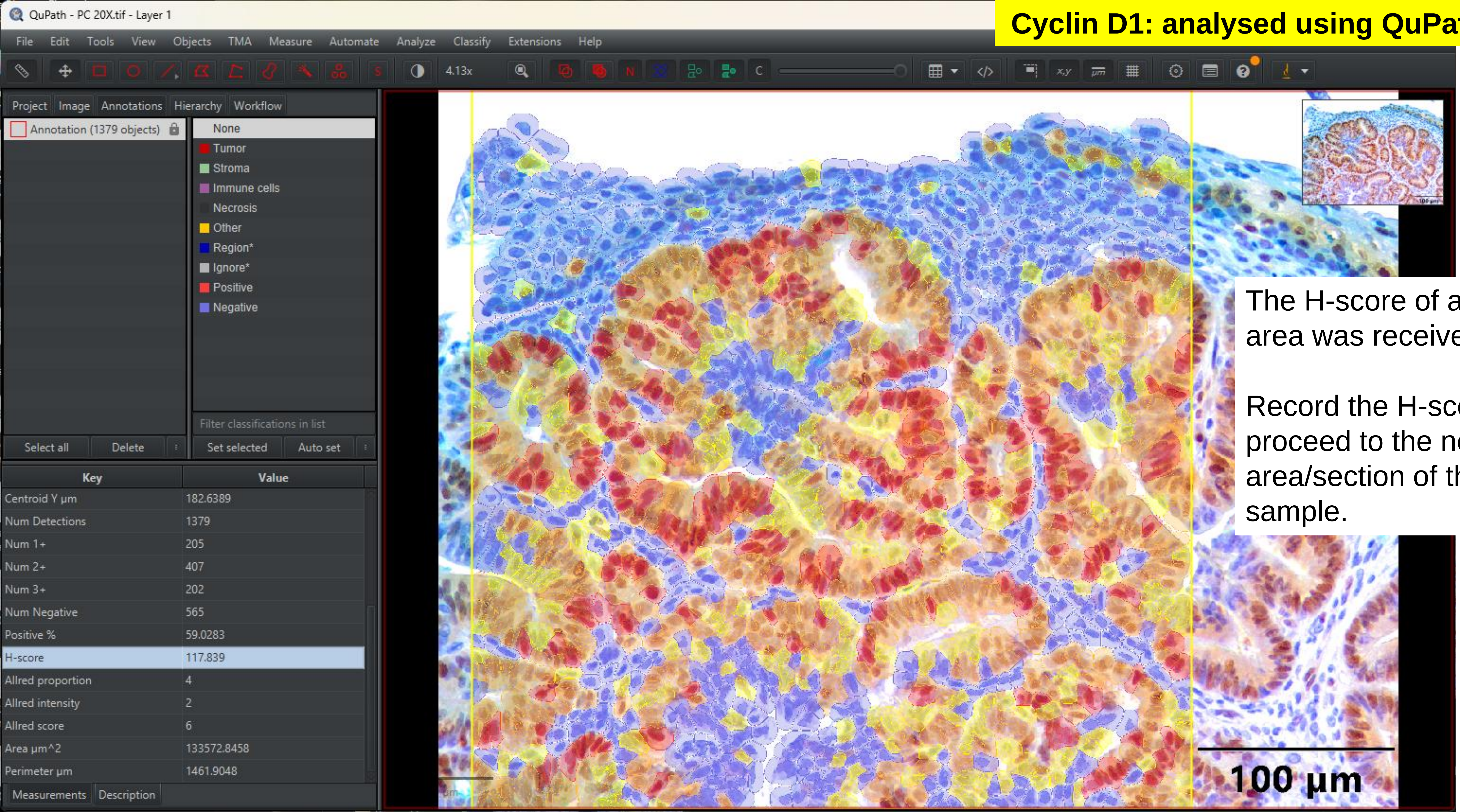

The H-score of a selected area was received.

Record the H-score and proceed to the next area/section of the same sample.

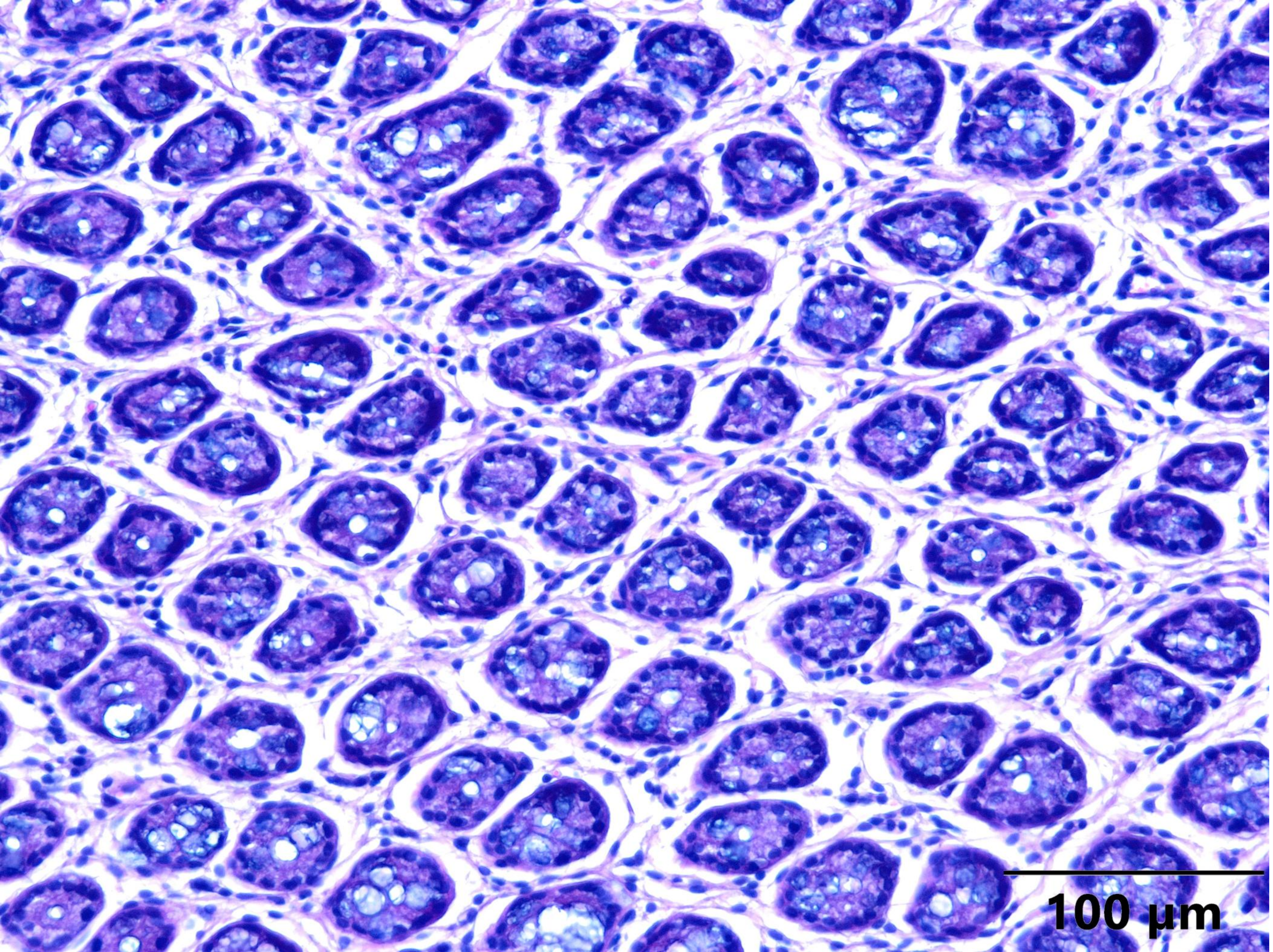

Original micrograph (NC)

Normoplasia

- Regular crypt appearance
- Well-differentiated crypts
- Goblet cells: present

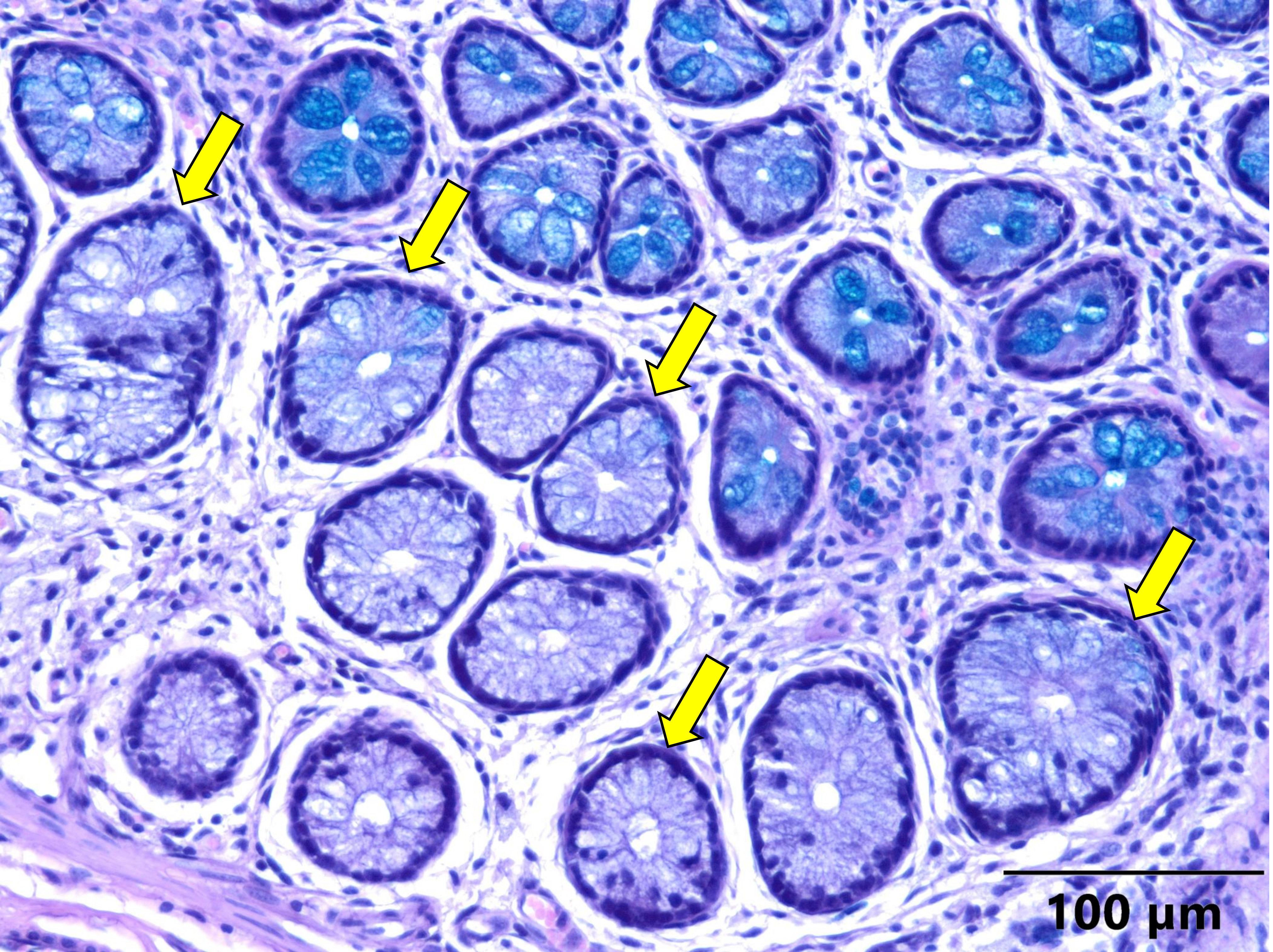

Original micrograph (LC)

Hyperplasia (arrows)

- Enlarged crypt appearance
- Somewhat differentiated crypts
- Goblet cells: present but reduced compared to the normal crypts
- Demonstrate mitotic figures, such nuclear stratification

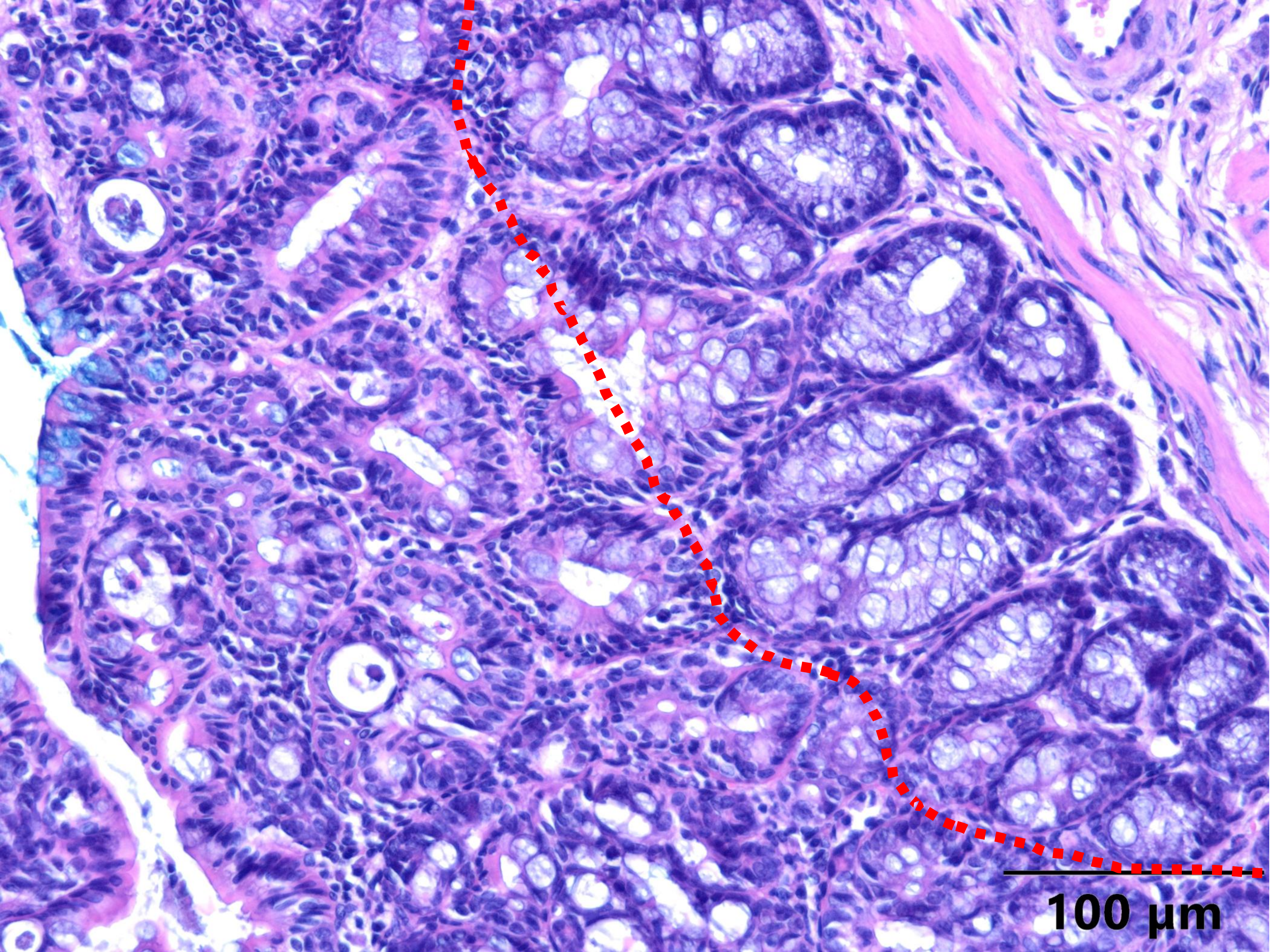

Dysplasia grading

Original micrograph (PC)

Low-grade dysplasia (Rt-sided)

- Aberrant crypt architecture
- Poor differentiated crypts
- Goblet cells: very low
- Nuclear stratification
- Columnar to cuboid shaped
- Increase N/C ratio

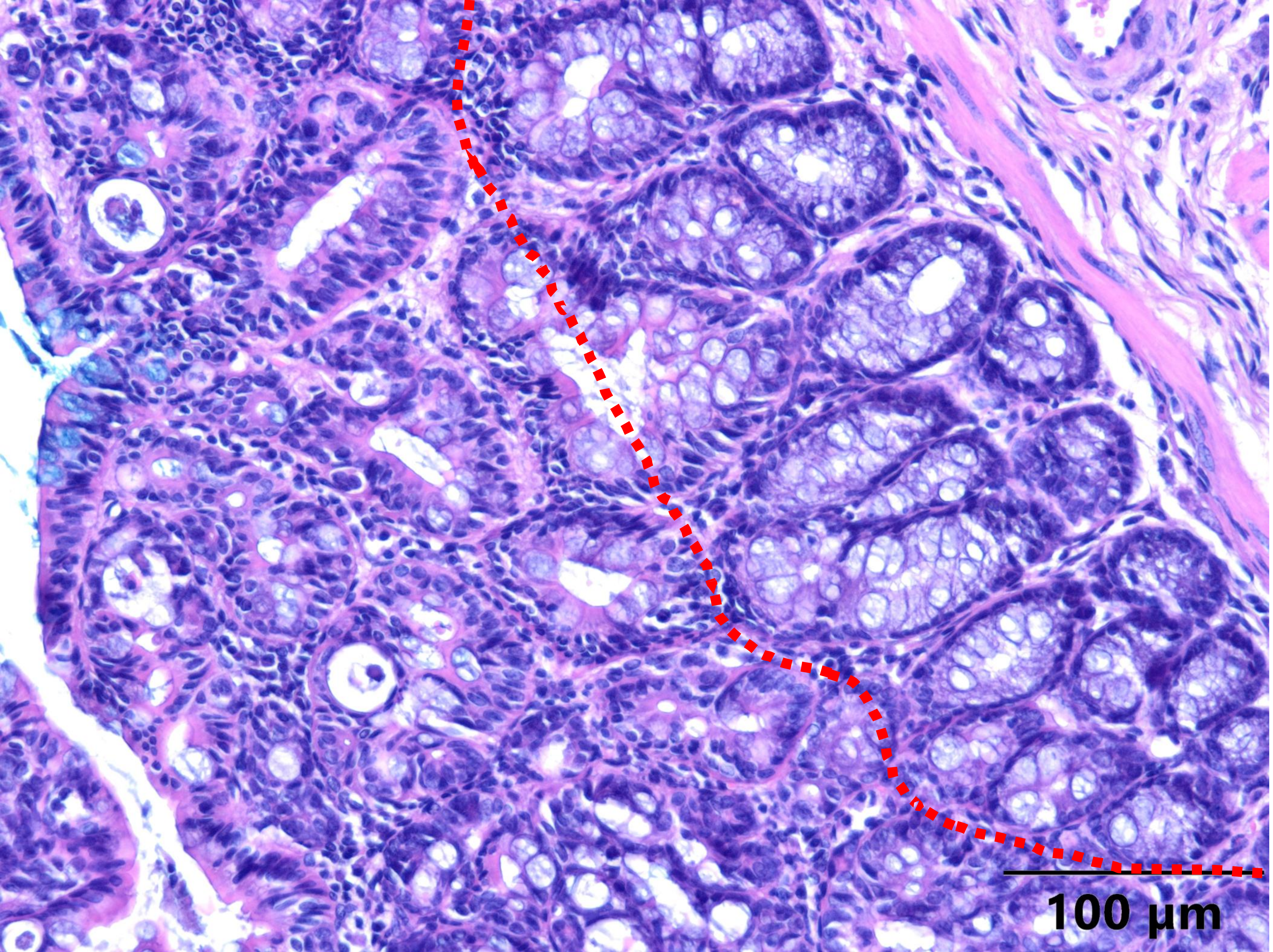

Original micrograph (PC)

High-grade dysplasia (Lt-sided)

- Distorted crypt structures
- Undifferentiated crypts
- Goblet cells: scarce
- Severe nuclear stratification
- Cuboid and squamous shaped
- Increase N/C ratio
