# Supplementary material for: Cold-pressed extraction of perilla seed oil enriched with alpha-linolenic acid mitigates tumour progression and restores gut microbial homeostasis in the AOM/DSS mice model of colitis-associated colorectal cancer
Source: PLoS One. 2024 Dec 9;19(12):e0315172. doi: 10.1371/journal.pone.0315172 (PMC11627366; doi:10.1371/journal.pone.0315172)
Supplement: S2 Raw images — (PDF) [file pone.0315172.s011.pdf]

β-catenin: raw

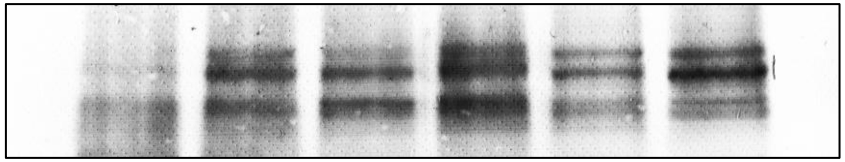

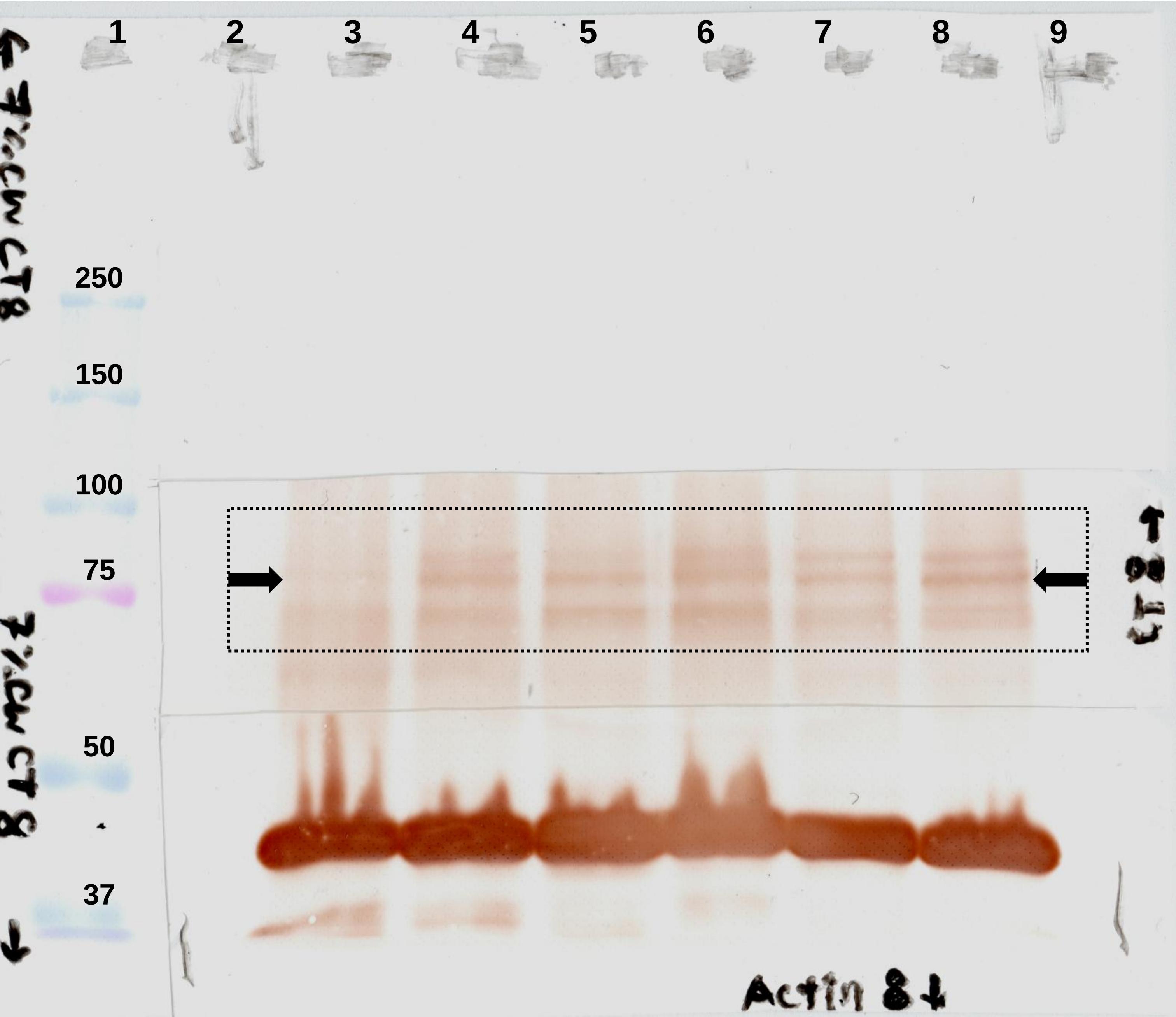

Load order

1. Prestained 10–250 kD (#1610374, Bio-Rad Laboratories, Inc)
2. -
3. NC
4. PC
5. LC
6. MC
7. HC
8. FC
9. -

Conditions

1. Gel: 7%
2. Protein conc.: 40 µg in each well
3. Stacking: 80 V, 30 min
4. Resolving: 100 V, 100 min
5. Transfer: semi-dry, 180 mA, 2 h
6. Membrane: Immun-Blot® 0.2 µm PVDF
7. Blocking: 5% NFDM in TBST, 1 h

Antibodies

1. Target: β-catenin, 1:1000 in 5% NFDM in TBST, incubated at 4 °C overnight
2. Control: β-actin, 1:1000 in 5% BSA in TBST, incubated at 4 °C overnight
3. HRP-2ndAb: 1:500 in TBST, incubated at RT for 2 h

Detection

1. ImmPACT® DAB Substrate Kit, Peroxidase (HRP) (SK-4105)
2. Exposure: 10 min at RT

β-catenin: inverted

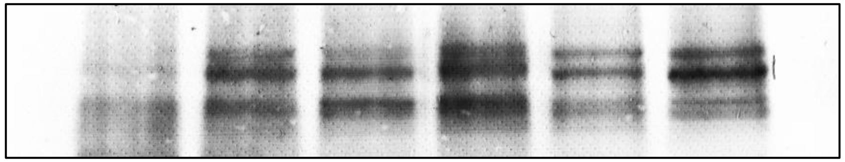

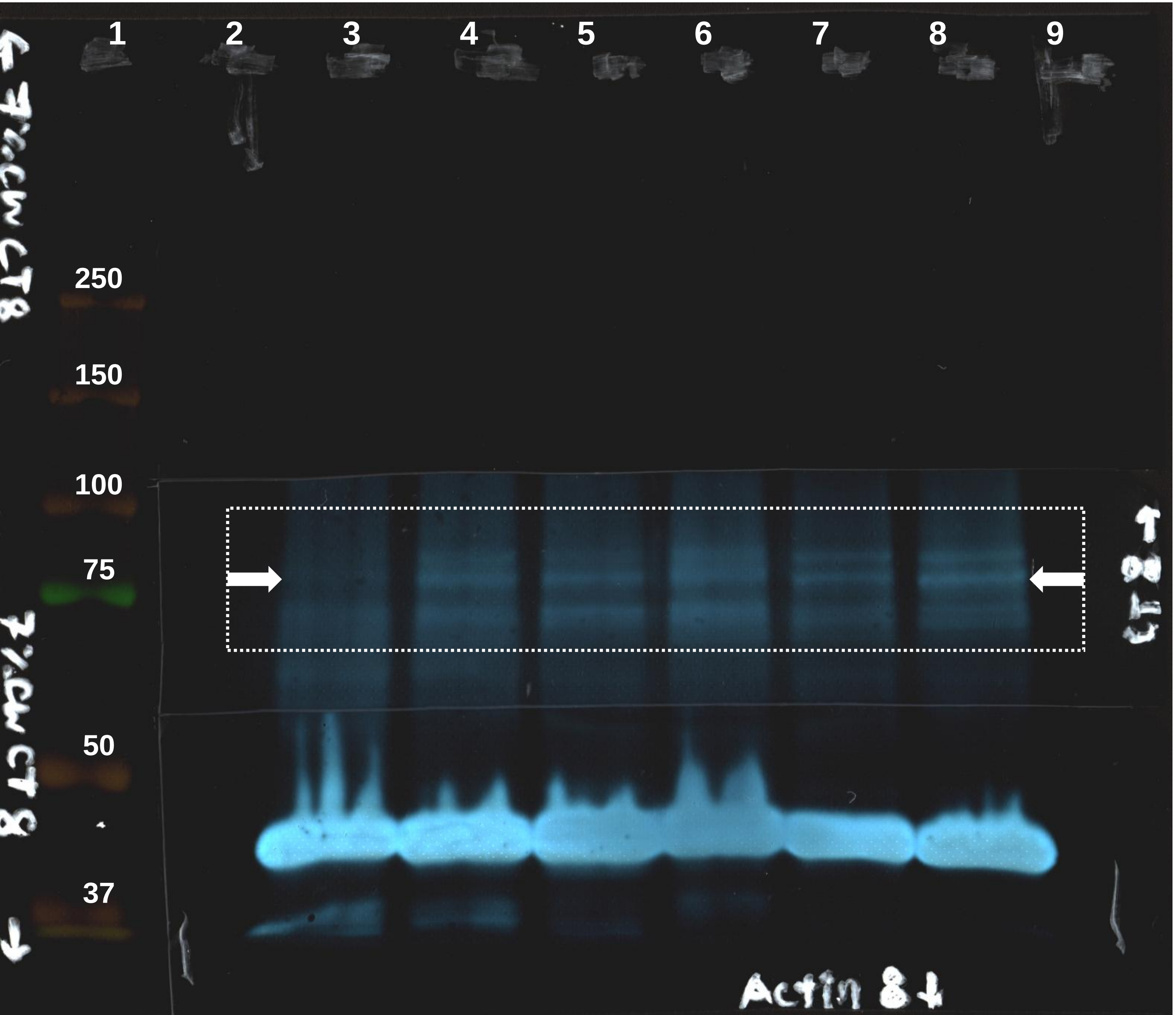

Load order

1. Prestained 10–250 kD (#1610374, Bio-Rad Laboratories, Inc)
2. -
3. NC
4. PC
5. LC
6. MC
7. HC
8. FC
9. -

Conditions

1. Gel: 7%
2. Protein conc.: 40 µg in each well
3. Stacking: 80 V, 30 min
4. Resolving: 100 V, 100 min
5. Transfer: semi-dry, 180 mA, 2 h
6. Membrane: Immun-Blot® 0.2 µm PVDF
7. Blocking: 5% NFDM in TBST, 1 h

Antibodies

1. Target: β-catenin, 1:1000 in 5% NFDM in TBST, incubated at 4 °C overnight
2. Control: β-actin, 1:1000 in 5% BSA in TBST, incubated at 4 °C overnight
3. HRP-2ndAb: 1:500 in TBST, incubated at RT for 2 h

Detection

1. ImmPACT® DAB Substrate Kit, Peroxidase (HRP) (SK-4105)
2. Exposure: 10 min at RT

COX-2: raw

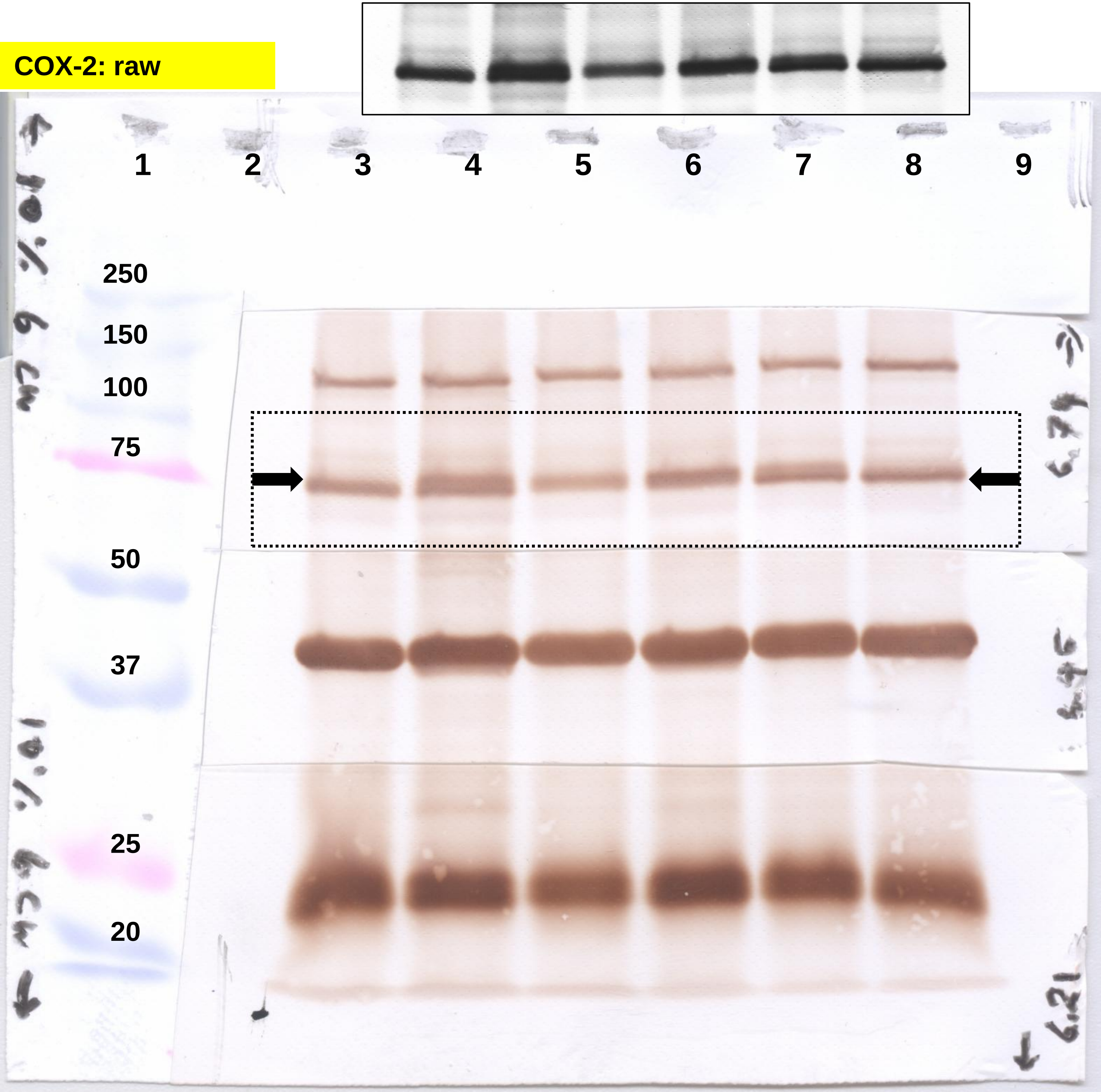

Load order

1. Prestained 10–250 kD (#1610374, Bio-Rad Laboratories, Inc)
2. -
3. NC
4. PC
5. LC
6. MC
7. HC
8. FC
9. -

Conditions

1. Gel: 10%
2. Protein conc.: 20 µg in each well
3. Stacking: 80 V, 30 min
4. Resolving: 100 V, 105 min
5. Transfer: semi-dry, 180 mA, 2 h
6. Membrane: Immun-Blot® 0.2 µm PVDF
7. Blocking: 5% NFDM in TBST, 1 h

Antibodies

1. Target: COX-2, 1:1000 in 5% NFDM in TBST, incubated at 4 °C overnight
2. Control: β-actin, 1:1000 in 5% BSA in TBST, incubated at 4 °C overnight
3. HRP-2ndAb: 1:500 in TBST, incubated at RT for 2 h

Detection

1. ImmPACT® DAB Substrate Kit, Peroxidase (HRP) (SK-4105)
2. Exposure: 10 min at RT

COX-2: inverted

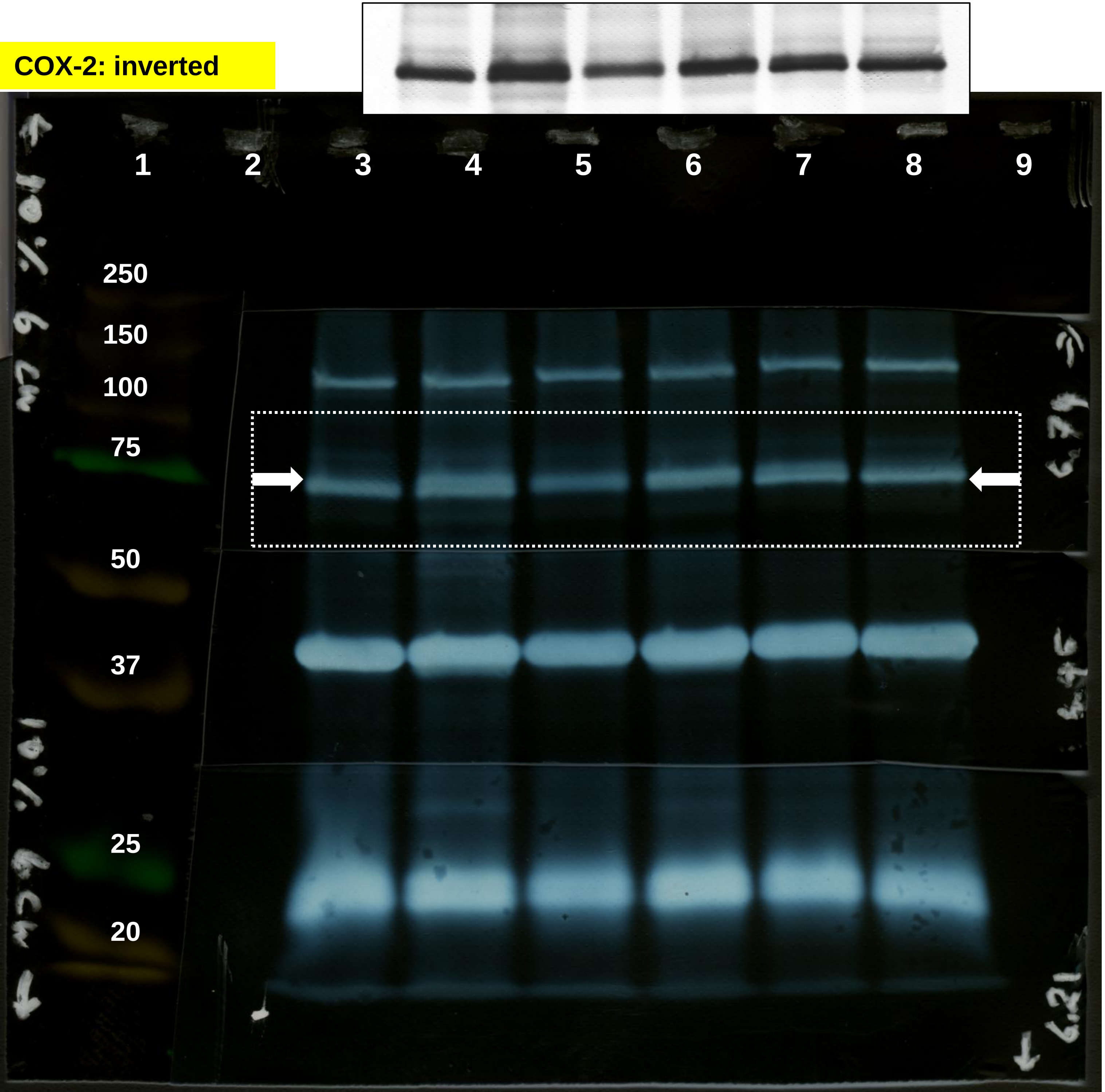

Load order

1. Prestained 10–250 kD (#1610374, Bio-Rad Laboratories, Inc)
2. -
3. NC
4. PC
5. LC
6. MC
7. HC
8. FC
9. -

Conditions

1. Gel: 10%
2. Protein conc.: 20 µg in each well
3. Stacking: 80 V, 30 min
4. Resolving: 100 V, 105 min
5. Transfer: semi-dry, 180 mA, 2 h
6. Membrane: Immun-Blot® 0.2 µm PVDF
7. Blocking: 5% NFDM in TBST, 1 h

Antibodies

1. Target: COX-2, 1:1000 in 5% NFDM in TBST, incubated at 4 °C overnight
2. Control: β-actin, 1:1000 in 5% BSA in TBST, incubated at 4 °C overnight
3. HRP-2ndAb: 1:500 in TBST, incubated at RT for 2 h

Detection

1. ImmPACT® DAB Substrate Kit, Peroxidase (HRP) (SK-4105)
2. Exposure: 10 min at RT

pNF-κB: raw

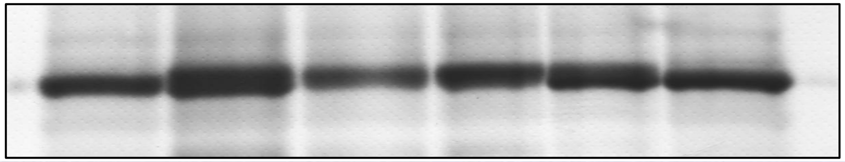

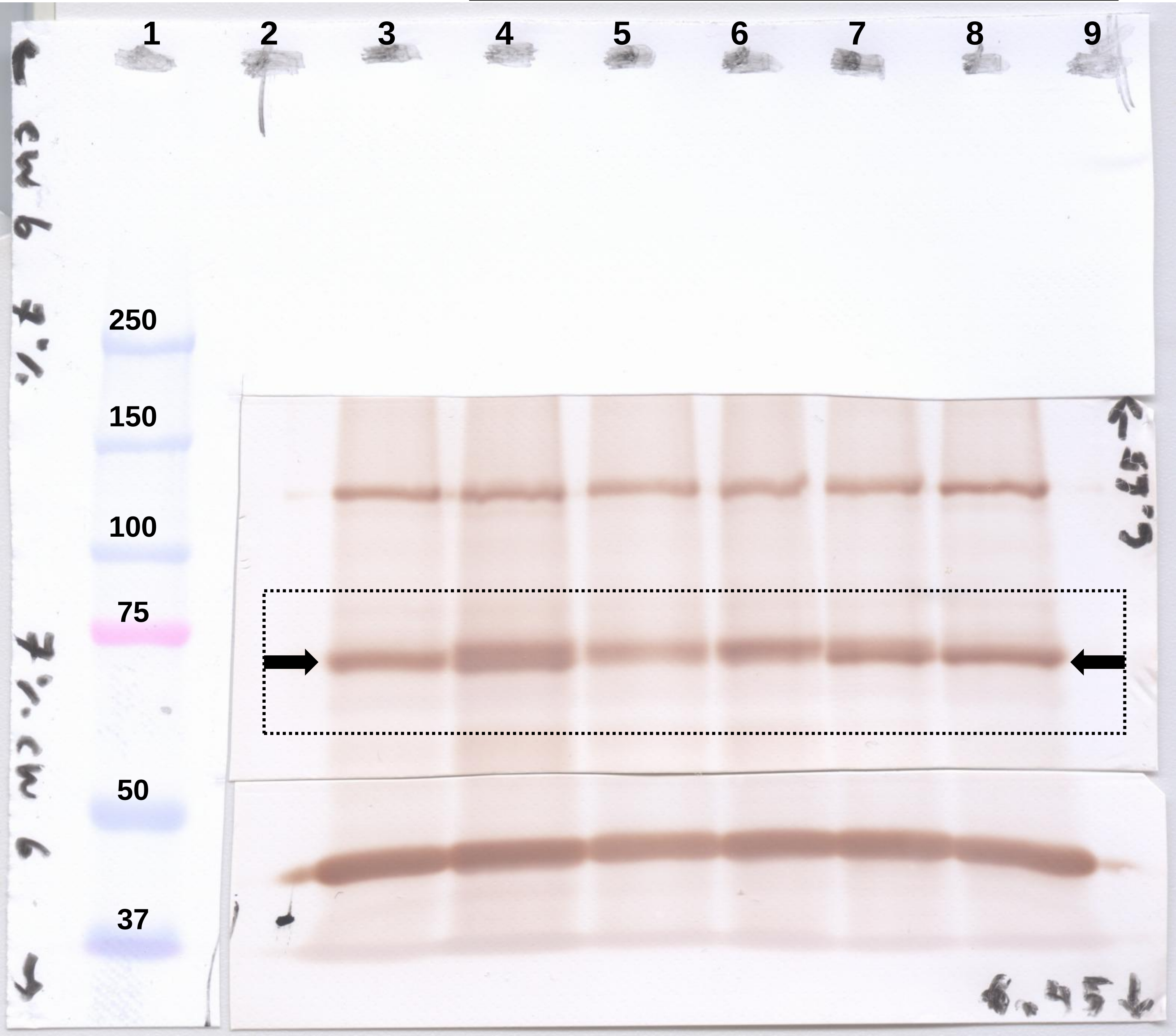

Load order

1. Prestained 10–250 kD (#1610374, Bio-Rad Laboratories, Inc)
2. -
3. NC
4. PC
5. LC
6. MC
7. HC
8. FC
9. -

Conditions

1. Gel: 7%
2. Protein conc.: 20 µg in each well
3. Stacking: 80 V, 30 min
4. Resolving: 100 V, 100 min
5. Transfer: semi-dry, 90 mA, 2 h
6. Membrane: Immun-Blot® 0.2 µm PVDF
7. Blocking: 5% NFDM in TBST, 1 h

Antibodies

1. Target: pNF-κB, 1:1000 in 5% BSA in TBST, incubated at 4 °C overnight
2. Control: β-actin, 1:1000 in 5% BSA in TBST, incubated at 4 °C overnight
3. HRP-2ndAb: 1:500 in TBST, incubated at RT for 2 h

Detection

1. ImmPACT® DAB Substrate Kit, Peroxidase (HRP) (SK-4105)
2. Exposure: 10 min at RT

pNF-κB: inverted

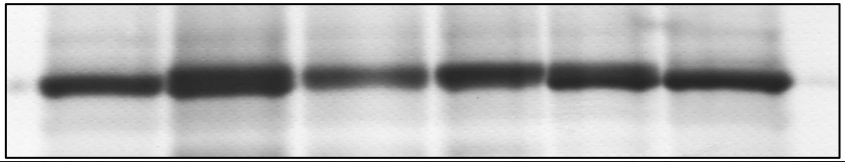

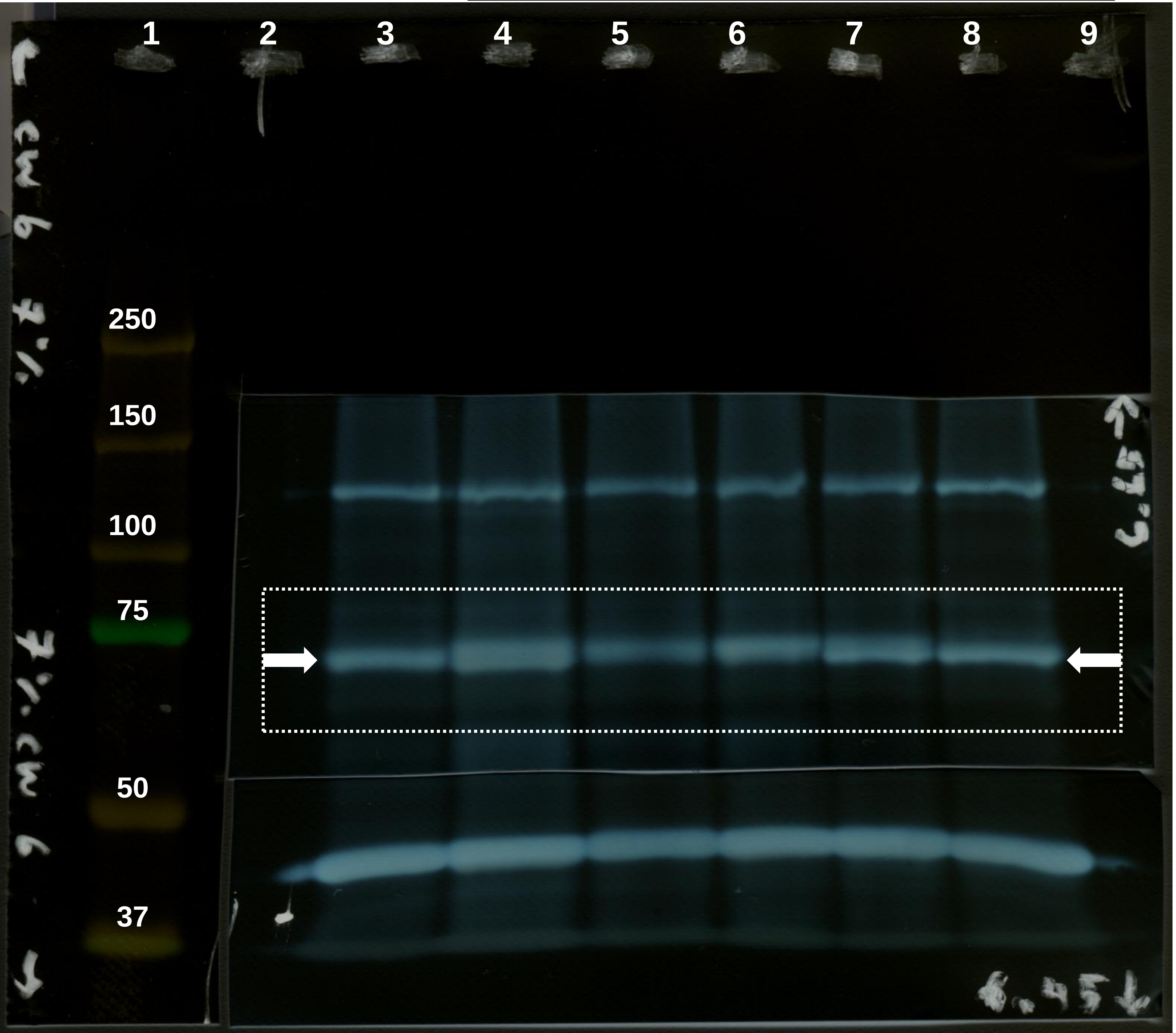

Load order

1. Prestained 10–250 kD (#1610374, Bio-Rad Laboratories, Inc)
2. -
3. NC
4. PC
5. LC
6. MC
7. HC
8. FC
9. -

Conditions

1. Gel: 7%
2. Protein conc.: 20 µg in each well
3. Stacking: 80 V, 30 min
4. Resolving: 100 V, 100 min
5. Transfer: semi-dry, 90 mA, 2 h
6. Membrane: Immun-Blot® 0.2 µm PVDF
7. Blocking: 5% NFDM in TBST, 1 h

Antibodies

1. Target: pNF-κB, 1:1000 in 5% BSA in TBST, incubated at 4 °C overnight
2. Control: β-actin, 1:1000 in 5% BSA in TBST, incubated at 4 °C overnight
3. HRP-2nd Ab: 1:500 in TBST, incubated at RT for 2 h

Detection

1. ImmPACT® DAB Substrate Kit, Peroxidase (HRP) (SK-4105)
2. Exposure: 10 min at RT

Ras: raw

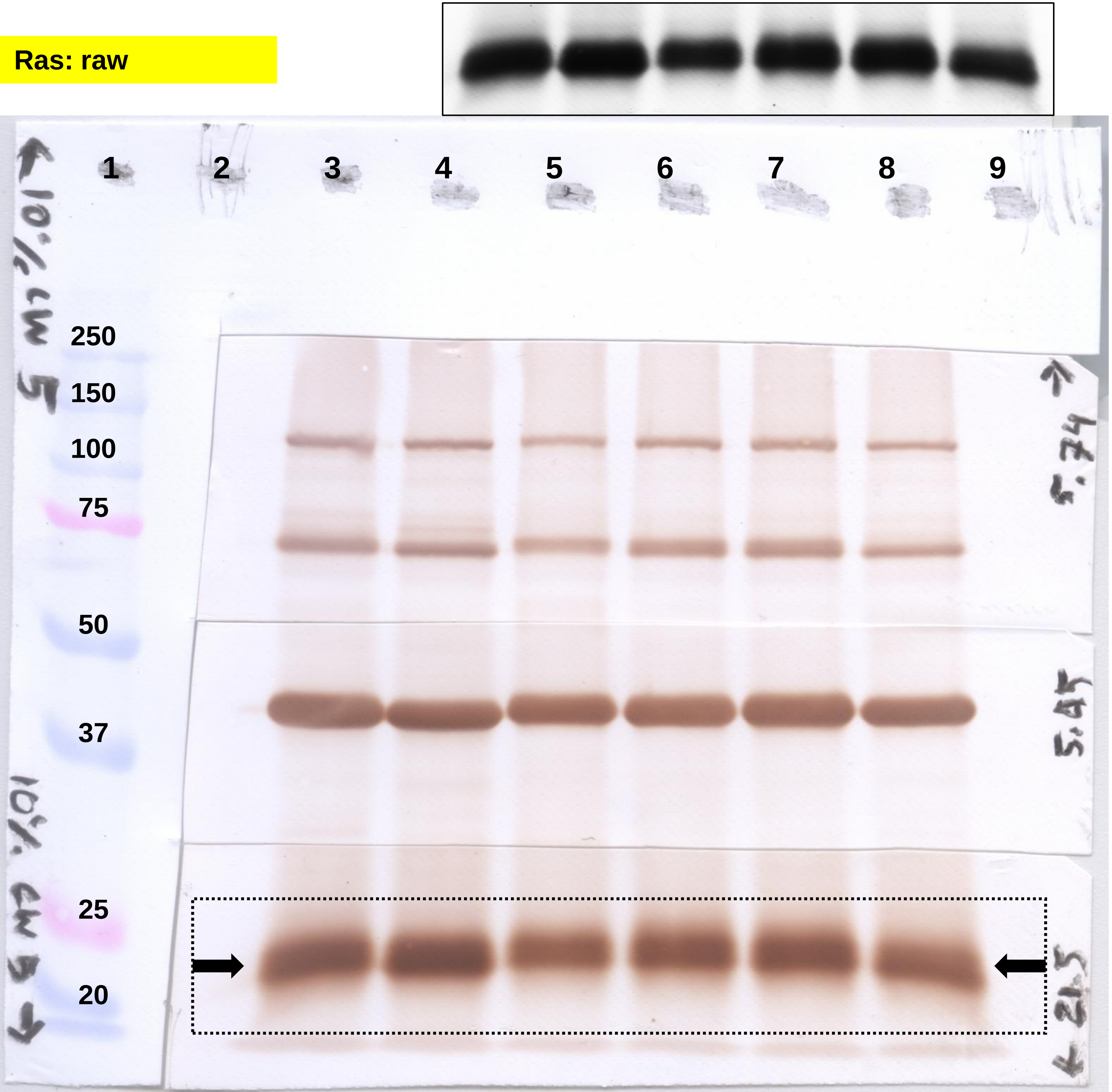

Load order

1. Prestained 10–250 kD (#1610374, Bio-Rad Laboratories, Inc)
2. -
3. NC
4. PC
5. LC
6. MC
7. HC
8. FC
9. -

Conditions

1. Gel: 10%
2. Protein conc.: 20 µg in each well
3. Stacking: 80 V, 30 min
4. Resolving: 100 V, 105 min
5. Transfer: semi-dry, 180 mA, 2 h
6. Membrane: Immun-Blot® 0.2 µm PVDF
7. Blocking: 5% NFDM in TBST, 1 h

Antibodies

1. Target: Ras, 1:1000 in 5% BSA in TBST, incubated at 4 °C overnight
2. Control: β-actin, 1:1000 in 5% BSA in TBST, incubated at 4 °C overnight
3. HRP-2ndAb: 1:500 in TBST, incubated at RT for 2 h

Detection

1. ImmPACT® DAB Substrate Kit, Peroxidase (HRP) (SK-4105)
2. Exposure: 10 min at RT

Ras: inverted

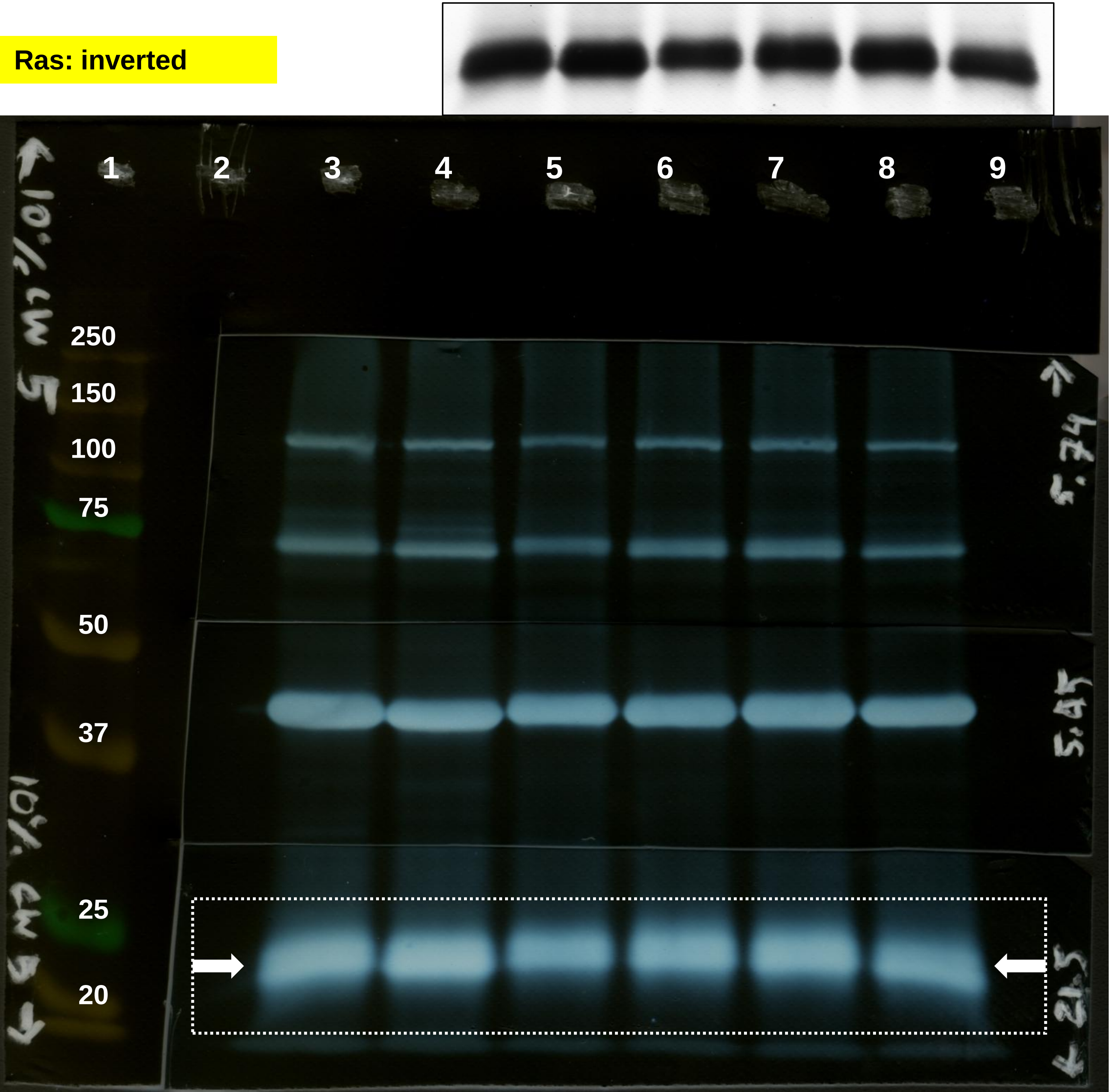

Load order

1. Prestained 10–250 kD (#1610374, Bio-Rad Laboratories, Inc)
2. -
3. NC
4. PC
5. LC
6. MC
7. HC
8. FC
9. -

Conditions

1. Gel: 10%
2. Protein conc.: 20 µg in each well
3. Stacking: 80 V, 30 min
4. Resolving: 100 V, 105 min
5. Transfer: semi-dry, 180 mA, 2 h
6. Membrane: Immun-Blot® 0.2 µm PVDF
7. Blocking: 5% NFDM in TBST, 1 h

Antibodies

1. Target: Ras, 1:1000 in 5% BSA in TBST, incubated at 4 °C overnight
2. Control: β-actin, 1:1000 in 5% BSA in TBST, incubated at 4 °C overnight
3. HRP-2ndAb: 1:500 in TBST, incubated at RT for 2 h

Detection

1. ImmPACT® DAB Substrate Kit, Peroxidase (HRP) (SK-4105)
2. Exposure: 10 min at RT

β-actin: raw

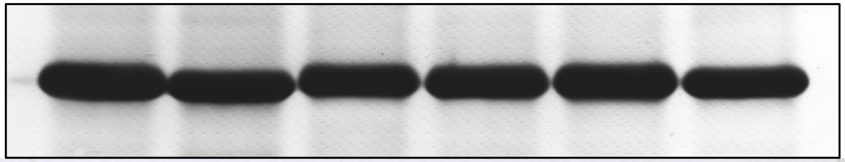

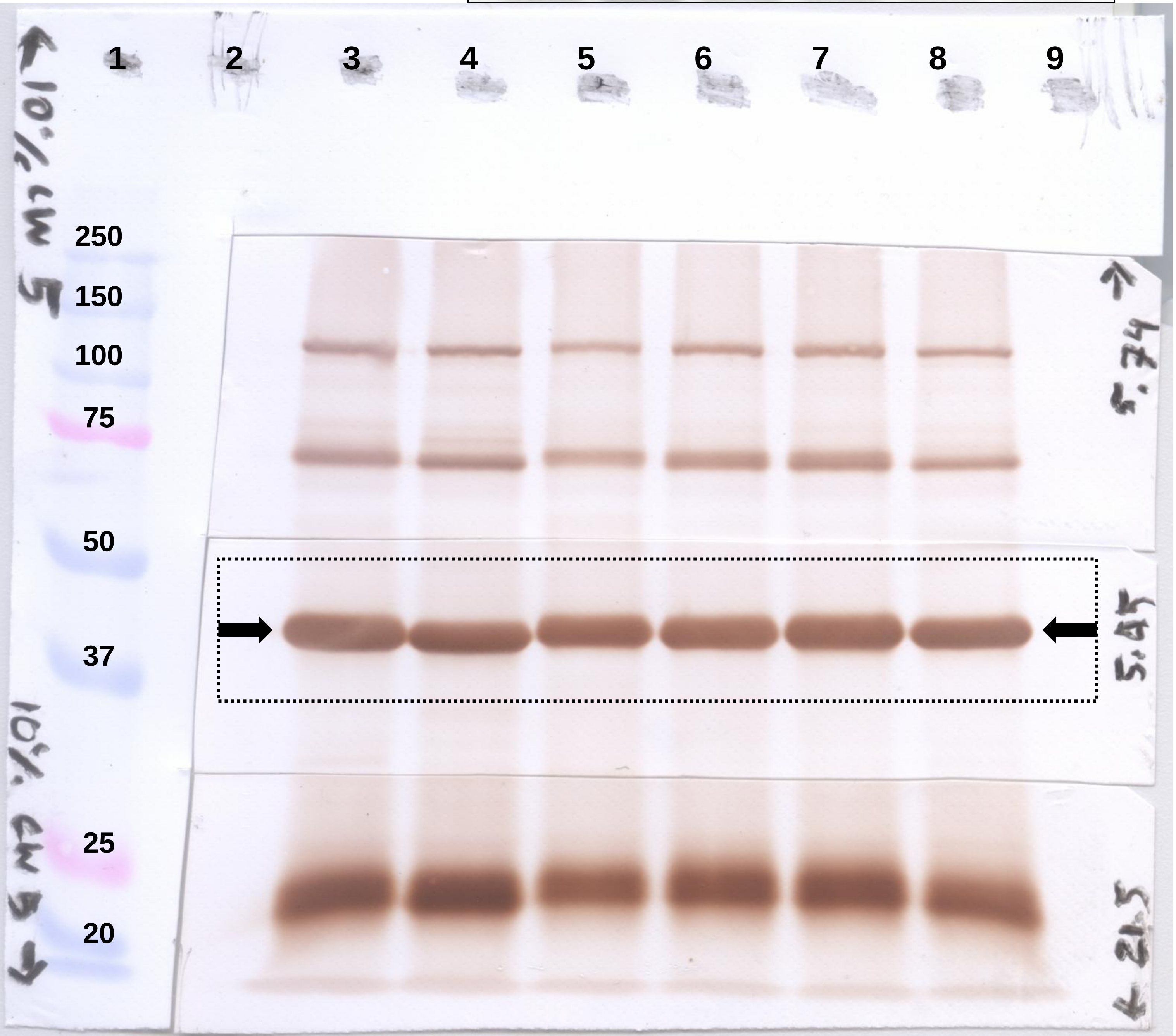

Load order

1. Prestained 10–250 kD (#1610374, Bio-Rad Laboratories, Inc)
2. -
3. NC
4. PC
5. LC
6. MC
7. HC
8. FC
9. -

Conditions

1. Gel: 10%
2. Protein conc.: 20 µg in each well
3. Stacking: 80 V, 30 min
4. Resolving: 100 V, 105 min
5. Transfer: semi-dry, 180 mA, 2 h
6. Membrane: Immun-Blot® 0.2 µm PVDF
7. Blocking: 5% NFDM in TBST, 1 h

Antibodies

1. Target: Ras, 1:1000 in 5% BSA in TBST, incubated at 4 °C overnight
2. Control: β-actin, 1:1000 in 5% BSA in TBST, incubated at 4 °C overnight
3. HRP-2ndAb: 1:500 in TBST, incubated at RT for 2 h

Detection

1. ImmPACT® DAB Substrate Kit, Peroxidase (HRP) (SK-4105)
2. Exposure: 10 min at RT

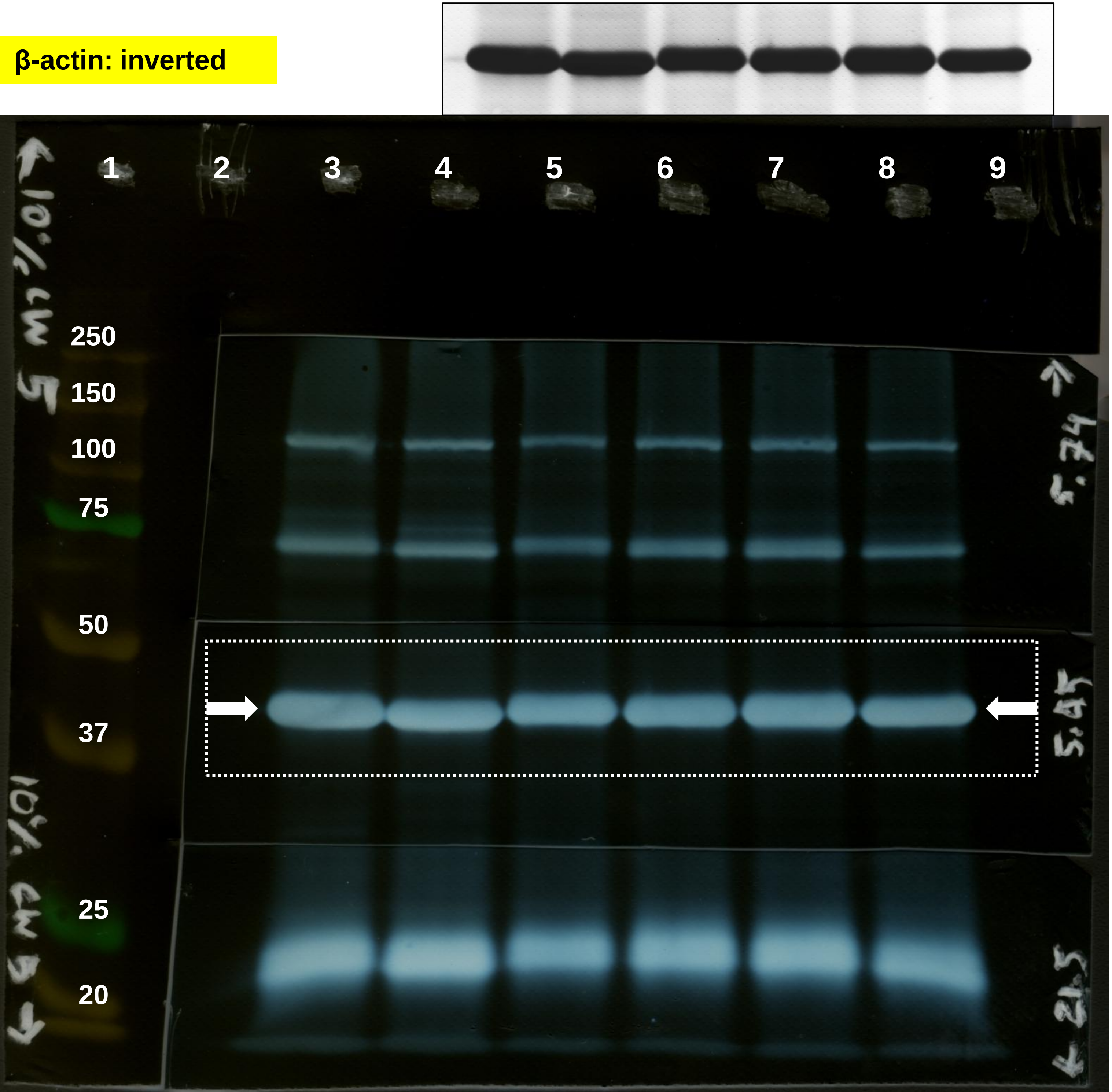

Load order

1. Prestained 10–250 kD (#1610374, Bio-Rad Laboratories, Inc)
2. -
3. NC
4. PC
5. LC
6. MC
7. HC
8. FC
9. -

Conditions

1. Gel: 10%
2. Protein conc.: 20 µg in each well
3. Stacking: 80 V, 30 min
4. Resolving: 100 V, 105 min
5. Transfer: semi-dry, 180 mA, 2 h
6. Membrane: Immun-Blot® 0.2 µm PVDF
7. Blocking: 5% NFDM in TBST, 1 h

Antibodies

1. Target: Ras, 1:1000 in 5% BSA in TBST, incubated at 4 °C overnight
2. Control: β-actin, 1:1000 in 5% BSA in TBST, incubated at 4 °C overnight
3. HRP-2ndAb: 1:500 in TBST, incubated at RT for 2 h

Detection

1. ImmPACT® DAB Substrate Kit, Peroxidase (HRP) (SK-4105)
2. Exposure: 10 min at RT

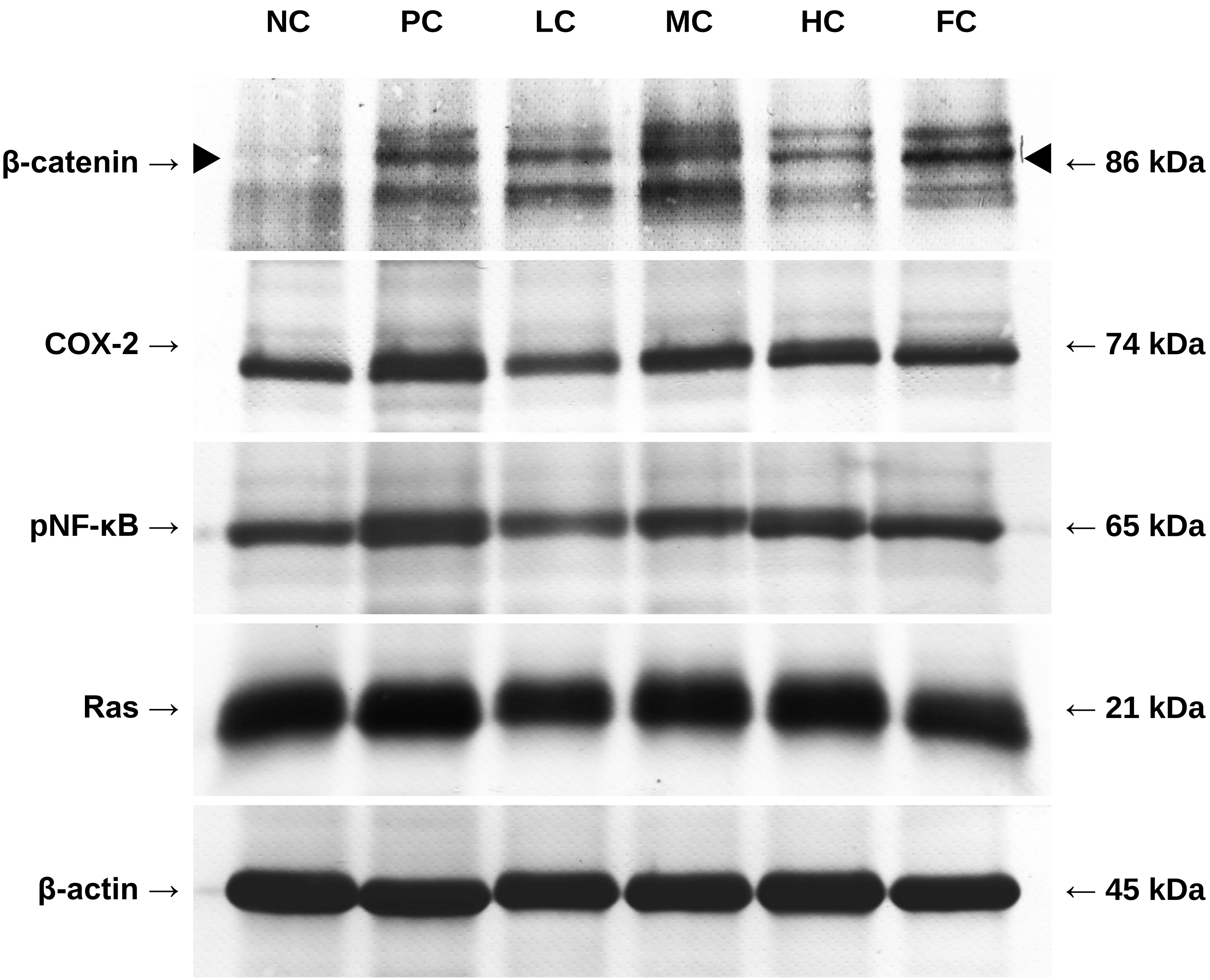
NC
PC
LC
MC
HC
FC
β-catenin →
← 86 kDa
COX-2 →
← 74 kDa
pNF-κB →
← 65 kDa
Ras →
← 21 kDa
β-actin →
← 45 kDa
