# Supplementary material for: Cold-pressed extraction of perilla seed oil enriched with alpha-linolenic acid mitigates tumour progression and restores gut microbial homeostasis in the AOM/DSS mice model of colitis-associated colorectal cancer
Source: PLoS One. 2024 Dec 9;19(12):e0315172. doi: 10.1371/journal.pone.0315172 (PMC11627366; doi:10.1371/journal.pone.0315172)
Supplement: S1 File — (PDF) [file pone.0315172.s012.pdf]

2 WANG LANG ROAD
BANGKOKNOI
BANGKOK 10700 THAILAND

Tel. (662) 419-5456
FAX (662) 418-3307

Siriraj Animal Care and Use Committee (SiACUC)

Certificate of Approval

COA No.: 015/2563

Protocol Title : Investigation on the effect of perilla seed oil on microbiota and miRNA in mouse model of colorectal cancer.

SiACUP No. : 004/2563

Principal Investigator / Affiliation : Assos. Prof. Dr. Aikkarach Kettawan / Institute of Nutition, Mahidol University

Research site : Faculty of Medicine Siriraj Hospital, Mahidol University

1. ANIMAL CARE AND USE PROTOCOL No. 004/2563
2. ....
3. ....

Renewal date (1) : July 14, 2021

Expired date : July 13, 2022

This is to certify that Siriraj Animal Care and Use Committee is in full Compliance with International Guidelines for Animal Research Protection such as International Guiding Principles for Biochemical Research Involving Animals.

.... 16 Feb 2021

(Prof. Dr.Prasert Auewarakul, M.D.) Date

Chairman

.... 19 Feb 2021

(Prof. Dr.Prasit Watanapa, M.D.) Date

Dean of Faculty of Medicine Siriraj Hospital

2 ถนนวังหลัง
บางกอกน้อย
กรุงเทพฯ 10700

โทรศัพท์ (662) 419-5456
โทรสาร (662) 418-3307

คณะกรรมการกำกับดูแลการเลี้ยงและใช้สัตว์

เอกสารรับรองโครงการวิจัย

หมายเลข 015/2563

ชื่อโครงการภาษาไทย : การศึกษาผลของน้ำมันเมล็ดงาม้อนไทยต่อการเปลี่ยนแปลงของแบคทีเรียในลำไส้และการแสดงออกของไมโครอาร์เอ็นเอในหนูทดลองที่ถูกเหนี่ยวนำให้เป็นโรคมะเร็งลำไส้ใหญ่

รหัสโครงการ : 004/2563

หัวหน้าโครงการ / หน่วยงานที่สังกัด : รองศาสตราจารย์ ดร.เอกราช เกตวัลห์
สถาบันโภชนาการ มหาวิทยาลัยมหิดล

สถานที่ทำวิจัย : คณะแพทยศาสตร์ศิริราชพยาบาล มหาวิทยาลัยมหิดล

เอกสารที่รับรอง :

1. โครงร่างการวิจัยตามใบสมัคร เลขที่ 004/2563
2. ....
3. ....

ต่ออายุครั้งที่ (1) : 14 กรกฎาคม 2564

วันที่หมดอายุ : 13 กรกฎาคม 2565

คณะกรรมการกำกับดูแลการเลี้ยงและใช้สัตว์ คณะแพทยศาสตร์ศิริราชพยาบาล มหาวิทยาลัยมหิดล ดำเนินการให้การรับรองโครงการวิจัยตามแนวทางหลักจริยธรรมการวิจัยในสัตว์ที่เป็นสากล ได้แก่ International Guiding Principles for Biochemical Research Involving Animals

.... 16 ก.ค. 2564
วันที่

(ศาสตราจารย์ ดร. นายแพทย์ประเสริฐ เอื้อวรากุล)
ประธานคณะกรรมการกำกับดูแลการเลี้ยงและใช้สัตว์

.... 19 ก.ค. 2564
วันที่

(ศาสตราจารย์ ดร. นายแพทย์ประสิทธิ์ วัฒนาภา)
คณบดีคณะแพทยศาสตร์ศิริราชพยาบาล
